# Impact and cost-effectiveness of scaling up HCV testing and treatment strategies for achieving HCV elimination among people who inject drugs in England: a mathematical modelling study

**DOI:** 10.1016/j.lanepe.2024.101176

**Published:** 2024-12-12

**Authors:** Zoe Ward, Ruth Simmons, Hannah Fraser, Adam Trickey, Jo Kesten, Andy Gibson, Leila Reid, Sean Cox, Fiona Gordon, Stuart Mc Pherson, Stephen Ryder, Javier Vilar, Alec Miners, Jack Williams, Beatrice Emmanouil, Monica Desai, Laura Coughlan, Ross Harris, Graham R. Foster, Matthew Hickman, Sema Mandal, Peter Vickerman

**Affiliations:** a Bristol Medical School, University of Bristol, Bristol, UK; b UK Health Security Agency, London, UK; c The National Institute for Health and Care Research (NIHR) Health Protection Research Unit (HPRU) in Behavioural Science and Evaluation, University of Bristol, Bristol, UK; d The National Institute for Health and Care Research Applied Research Collaboration West (NIHR ARC West) at University Hospitals Bristol and Weston NHS Foundation Trust, Bristol, UK; e University of West of England, Bristol, UK; f The Hepatitis C Trust, London, UK; g University Hospitals Bristol, Bristol, UK; h Newcastle Upon Tyne Hospitals NHS Foundation Trust, Newcastle, UK; i Nottingham University Hospitals NHS Trust, Nottingham, UK; j Manchester University NHS Foundation Trust, Manchester, UK; k London School of Hygiene and Tropical Medicine, London, UK; l NHS England, UK

**Keywords:** Hepatitis C, People who inject drugs, Direct acting antiviral treatment, Elimination, UK

## Abstract

**Background:**

England aims to reach the World Health Organization (WHO) elimination target of decreasing HCV incidence among people who inject drugs (PWID) to <2 per 100 person-years (/100pyrs) by 2030. We assessed what testing and treatment strategies will achieve this target and whether they are cost-effective.

**Methods:**

A dynamic deterministic HCV transmission model among PWID was developed for four England regions, utilising data on the scale-up of HCV treatment among PWID in prisons, drug treatment centres (DTC, where opioid agonist therapy is provided), and any other setting (e.g., primary care). The model projected whether the elimination target will be reached with existing testing and treatment initiatives (‘status quo’ model, SQ), or whether improvements are needed from 2024. Cost data was collated through practitioners' interviews and published literature. The mean incremental cost-effectiveness ratio (ICER per quality adjusted life year (QALY) saved, 50-year time horizon; 3.5% discount rate) of SQ (assumes counterfactual of no treatment scale-up post-2015) and improved model (counterfactual: SQ model) was compared to a willingness-to-pay threshold of £20,000/QALY saved.

**Findings:**

The SQ model projects HCV incidence will decrease by 79.7–98.6% (range of medians) over 2015–2030 to 0.2–2.2/100pyrs, with an ICER of £308–1609/QALY saved across the regions. There is >80% probability of achieving the incidence target in three regions, and 40% probability in the other region. If annual testing in DTC increases to 80% (from 27%) or 75% of people get tested during their prison stay (from 55%) from 2024 in the lower impact region, then their probability increases to >65%, with both strategies being highly cost-effective.

**Interpretation:**

Many England regions could reach the WHO HCV elimination target by 2030 under existing testing and treatment pathways. Scaling up of testing in DTC or prisons will help achieve this target and is highly cost-effective.

**Funding:**

10.13039/501100000272NIHR.

**Research in context**

**Evidence before this study**

We searched PubMed for studies that modelled the impact and/or cost-effectiveness of achieving HCV elimination among people who inject drugs (PWID), published up to 3rd May 2024, using the terms ‘(hcv OR hepatitis) AND elimination AND (model∗ OR cost-effectiveness) AND (“PWID” OR “IDU” OR “IVDU” OR “injection drug” OR “injecting drug” OR “intravenous drug” OR “people who inject drugs”)’. No language restriction was applied. The search found 241 records of which we identified 39 relevant articles that modelled the impact of scaling up HCV treatment to achieve HCV elimination or a large decrease in HCV transmission/prevalence among PWID. Only 11 studies evaluated the impact of an ongoing elimination initiative, with most studies (28) modelling what future scale-up is needed to achieve their elimination target. Only 16 studies evaluated the impact of HCV testing strategies, with none evaluating the impact of multiple testing pathways. Economic analyses (8 studies) generally evaluated the cost-effectiveness of a specific or general screening/treatment strategy among PWID, with only 2 studies (both Australia) considering the potential cost-effectiveness of an ongoing elimination initiative, but without considering multiple testing and treatment pathways. The two Australian studies showed the favourable cost-effectiveness achieved with ongoing elimination strategies with one showing that testing needed to be increased to reach elimination and the other showing that current levels of treatment in prison and community are sufficient to reach elimination. Other studies that considered ongoing elimination initiatives generally suggest that most countries need to scale-up treatment (and sometimes testing) to achieve the elimination targets, with only recent analyses from Australia and Norway, and an older analysis including Slovenia and Amsterdam (from 2017), suggesting they were already on target to achieve elimination.

**Added value of this study**

Our analysis presents one of the first impact and cost-effectiveness analyses of an ongoing country-level HCV elimination initiative among PWID. To our knowledge, no previous analyses have considered the testing and treatment undertaken in different service settings and regions, and what is required in these service settings to achieve elimination. We showed that HCV testing, linkage-to-care and HCV treatment among PWID at drug treatment centres and prisons have scaled up substantially in England since 2016. We show that this scale-up has decreased HCV incidence among PWID by 56.1–85.4% over 2016–2023, and is sufficient to reach the WHO elimination target in most regions by 2030. We project that viraemic prevalence can be used to determine who is not on target, and for these settings we show that the best strategy for ensuring they achieve elimination is to increase testing in drug treatment centres (80% of attendees tested annually), which we show is highly cost-effective.

**Implications of all the available evidence**

Our findings are important for stakeholders in the UK because they show the progress that has been made and give guidance on what needs to be done to achieve HCV elimination among PWID. For other countries, we provide evidence on the impact and cost-effectiveness of scaling up interventions to achieve elimination among PWID and give pointers on the levels of testing and treatment that are needed.

## Introduction

Hepatitis C virus (HCV) infection causes substantial liver-related morbidity.^1^ In England, it is estimated that 92,900 people are chronically HCV-infected in 2021 with most (90%) HCV infections resulting from injecting drug use (IDU).^2^^,^^3^

Although evidence suggests opioid agonist therapy (OAT) and needle and syringe programmes (NSP) decrease HCV transmission among people who inject drugs (PWID),^4^ modelling suggests that HCV treatment must be scaled up to decrease HCV transmission to low levels.^5^

Direct-acting antiviral (DAA) HCV therapies have transformed the prevention and treatment landscape for HCV, leading the World Health Organization (WHO) to develop a global health strategy to eliminate HCV as a public health threat.^6^ This strategy includes a target to reduce HCV incidence among PWID to <2 per 100 person-years (/100pyrs) by 2030.^6^ Many countries have initiated HCV elimination programmes,^7^ with emerging data suggesting that the scale-up of DAA therapies has had impact in some settings.^8^^,^^9^ Although uncertain, data on HCV incidence in England suggests it is high, at >10/100pyrs in 2022.^2^

In 2019, England negotiated an HCV elimination tender with the pharmaceutical industry, including an investment to scale-up HCV treatment and case-finding strategies.^10^ This led to an expansion in testing and treatment interventions in settings where PWID can be reached, including in drug treatment centres (DTC) and prisons.^11^ Drug treatment centres provide services for PWID including information, harm reduction interventions (such as NSP, OAT, blood-borne virus testing and hepatitis B vaccination), recovery planning in conjunction with a range of pharmacological, psychosocial and structured treatment programs.

We used mathematical modelling to evaluate the impact and cost-effectiveness of HCV testing and treatment strategies among PWID in four regions of England. We assessed whether the WHO incidence target will be reached by 2030 with existing strategies and, if not, what improvements in testing and linkage-to-treatment are needed to reach them.

## Methods

This analysis involves numerous steps. We used empirical data to determine how the testing and treatment pathway through different services has evolved for PWID over 2015–2020, and collated cost data on existing testing and treatment pathways for PWID in four England regions. We developed, parameterized and calibrated an HCV transmission model to assess the impact of the existing pathways in these England regions up to 2030, and if they were improved from 2024. We undertook cost-effectiveness analyses of these existing and improved testing and treatment pathways to determine the optimal strategy for achieving the WHO elimination targets.

### Model description

**Fig. 1:**
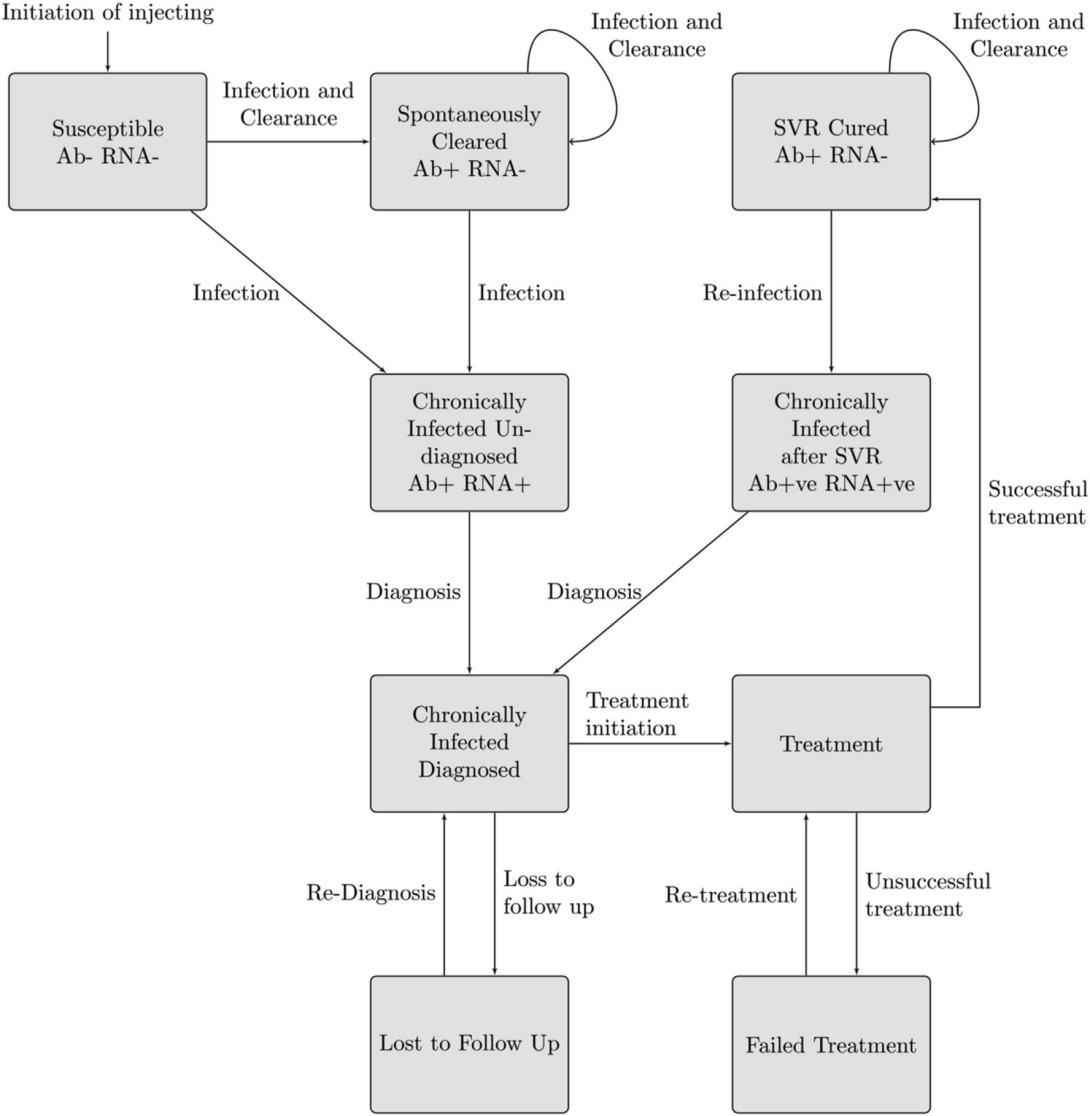
Model schematic of the infection and testing and treatment pathway, which is replicated for each testing setting (prison, drug treatment centres and other settings where testing can occur). The figure does not show cessation or mortality or other model stratifications (incarceration, homelessness, opioid agonist therapy, needle and syringe provision and HCV disease progression) which are shown Supplementary Fig. 1, Supplementary Materials pp 5–8.

We developed a dynamic deterministic compartmental model of HCV transmission for four England regions, termed operational delivery networks (ODNs). ODNs manage HCV treatment prescribing in their region.^12^ The modelled population was stratified by incarceration status, OAT and NSP uptake, homelessness status, infection and disease progression, and the testing and treatment pathway (Fig. 1 and Supplementary Fig. 1). These strata included factors that affect HCV risk and settings where HCV testing occurs. A detailed model description is in the Supplementary Materials.

Individuals enter the model through initiating IDU as susceptible PWID, with a proportion entering each incarceration and homelessness compartment, but none being on OAT or NSP when they start injecting. Individuals leave the model through permanently ceasing injecting or premature mortality. We include drug- and HCV-related mortality. We assume the initiation of new PWID balances the exit rate (except HCV-related mortality) over our evaluation period as evidence suggests a relatively stable PWID population size in UK over recent years.^13^ We do not explicitly model temporary cessation of injecting, but assume it is included in the model through modelling such things as OAT.

HCV risk is higher among PWID following recent release from prison or if currently homelessness,^14^^,^^15^ while incarcerated PWID have a different HCV risk to other PWID. There is increased mortality following prison release.^16^ Homelessness reduces retention on OAT.^17^

HCV risk is decreased among PWID using NSP or OAT.^4^ All PWID can initiate OAT, but at differing rates in prison or community, and then leave OAT at a fixed rate, with some individuals leaving OAT when they are incarcerated or released from prison. All-cause mortality is heightened when starting or stopping OAT, but decreased when on OAT.^18^ Being on OAT in the community reduces the risk of becoming incarcerated,^19^ whilst being on OAT in prison reduces the mortality upon release.^20^ All PWID in the community start and stop using NSP at fixed rates. NSP is not available in prisons.

PWID transmit HCV to PWID in their setting (community or prison) at a rate depending on the setting's HCV prevalence. Following HCV acquisition, some individuals recover whilst the remainder become chronically infected.^21^ Since 2016, a scale-up of HCV testing initiatives in prison and community DTC (through which OAT is provided) has occurred in England, which we model explicitly. HCV testing also occurs in other settings (e.g., pharmacy, primary care), but these are generally smaller scale, undertaken through local DTC, or are not PWID focused, and so are not modelled explicitly. We modelled specific HCV testing rates for PWID attending DTC or in prison, and an additional ‘other’ testing rate for PWID in the community (i.e., not in prison). Diagnosed individuals start HCV treatment at a rate dependent on where they were diagnosed, with the remainder being lost-to-care. Following HCV treatment, a proportion are cured (sustained virological response; SVR), with the remainder failing treatment. The risk of reinfection incidence and primary incidence are determined by their exposure to OAT, NSP, recent incarceration and homelessness, and so can vary but will be the same for each of these exposures.

### Model parameterisation and calibration

Data from multiple sources were used to parameterise and calibrate the model for four ODN regions in England: Greater Manchester, Northeast and Cumbria, Nottingham, and Bristol and Severn, which make up 20% of the overall population and 26% of the PWID population of England.^22^ Table 1 summarises characteristics of these regions. The main data source was the unlinked anonymous monitoring survey (UAM) for 2011–2019.^24^ This is a yearly cross-sectional survey undertaken among PWID attending harm reduction services in England, Wales and Northern Ireland (n = 5457 over our four ODN for 2011–2019). The survey includes questions on drug use behaviours, intervention uptake, homelessness and incarceration, with dried blood spots being used for testing of HCV antibodies and HCV RNA. Population size estimates came from the UK Health Security Agency (UKHSA)^22^ with ±50% bounds being included because estimates are uncertain.^25^

**Table 1:** Summary of main characteristics of each modelled region (showing point estimate or range across estimates from different years).

Baseline characteristic	Bristol and Severn ODN	Northeast and Cumbria ODN	Greater Manchester ODN	Nottingham ODN	Source
Overall population size	1,996,800	3,050,390	3,034,110	2,504,740	^22^
PWID population size	6180	13,310	8900	8840	^22^
Baseline HCV antibody prevalence among PWID^a^	36.0–57.3%	36.3–40.6%	55.7–57.8%	47.5–55.4%	UAM survey; analyses for this study
Baseline HCV RNA prevalence among HCV antibody positive PWID^a^	57.0–59.6%	45.9–47.1%	52.3–63.5%	51.6–57.8%	UAM survey; analyses for this study. Also use higher estimate from sentinel surveillance^23^
OAT coverage (currently)	59.7–69.3%	60.9–71.5%	79.3–88.9%	65.7–78.2%	UAM survey; analyses for this study
NSP coverage (% PWID used NSP in last year)^c^	88.2–94.3%	90.4–94.6%	83.6–90.1%	88.9–94.0%	UAM survey; analyses for this study
% ever incarcerated	66.6–71.8%	65.6–73.9%	70.6–78.7%	74.3–78.7%	UAM survey; analyses for this study
% currently homeless	19.8–37.6%	17.4–32.0%	20.0–29.8%	22.2–25.0%	UAM survey; analyses for this study
% ever homeless	80.0–88.6%	73.9–77.8%	75.0–80.6%	75.7–81.8%	UAM survey; analyses for this study
Total number of treatments among PWID 2016–2022^b^	1451–1531	3355–3429	2422–2767	1603–1962	Treatment and prescription database; analyses for this study

These values are not used in the model parameterisation and calibration, with the estimates used being in the Supplementary Tables 15–18.

a Baseline refers to estimates before 2016 whereas other estimates come from 2011 to 2019 for UAM data and 2016–2022 for treatment data.

b Includes adjustment for prescription data and range based on uncertainty in how many of prison treatments are among PWID.

c Note that coverage of NSP in terms of % of PWID that obtain enough needles or syringes for all their injections is lower at between 42.9 and 61.3% across ODNs.

UAM data for multiple years were combined for the periods 2011–2013, 2014/2015, 2016/2017, and 2018/2019 to calculate most parameter and calibration estimates for each ODN (sample size range n = 126–720 for each time-period and ODN). Additional data for Bristol came from previous surveys (2004–2009; Supplementary Table 15b). To maximise precision, data across all available years of the UAM survey were used to estimate various odds ratios (OR) used in the model calibration (Supplementary Materials). A national study undertaken among people attending harm reduction services in 2022 provided additional HCV prevalence estimates (the ‘needs assessment study’; Supplementary Materials).

Data on treatment numbers since 2016 came from the National Health Service (NHS) treatment database,^26^ including information on referral setting and IDU status. To estimate treatments among current PWID, imputed data was used because this variable was sometimes missing (∼20%; Supplementary Materials). Multiple imputation chained equations were used, using 10 imputed datasets. All prediction equations included all other variables (age, sex, ethnicity, country of birth, source of referral, treatment setting and disease stage) as predictors, plus year and ODN region. The resulting imputed datasets were then aggregated and estimates averaged across the 10 imputed datasets. However, because injecting risk status is also likely to be under-reported in the treatment database, we assumed all treatments in DTC were among individuals that have current or existing injecting risk (i.e., are defined as a PWID in our model) and allowed uncertainty in the number of treatments in prisons that were among PWID, varying it between the imputed number and the total number undertaken in prison. Unfortunately, because completion rates for the treatment database reduced from 2020, it was supplemented with treatment prescription data for 2020–2022.

UKHSA sentinel surveillance collates data on HCV testing in England, covering ∼40% of all tests in England and most testing undertaken in DTC.^23^^,^^27^ Through linking this surveillance dataset with the treatment database, we estimated the proportion of diagnosed individuals (RNA-positive test result) who initiated treatment, the proportion that achieved SVR, and the time from diagnosis to initiating treatment. Because of small numbers in each ODN, these measures were estimated across all ODN regions for three time periods (2015–2016, 2017–2018 and 2019–2020) and referral from DTC, prison, and other settings. Adjustment factors were used to account for ODN differences (Supplementary Table 17b–17e). Unfortunately, we could not estimate testing rates for PWID from the sentinel surveillance dataset because it has partial coverage and does not record IDU status. Testing rates for each ODN and referral setting were estimated through model calibration to give the observed number of treatments, with testing rates changing in 2016 and 2017.

Treatment estimates were not available pre-2015, but are assumed to be low. Testing rates pre-2015 were estimated for prisons and DTC through estimating a relative decrease in testing pre-2015 compared to post-2015 based on sentinel surveillance.^23^ To estimate numbers being treated pre-2015, we then used historical estimates (2005–2014) for the proportion of diagnosed individuals that initiate treatment for prisons and DTC.^23^

We calibrated the model for each ODN using an approximate Bayesian computation sequential Monte Carlo scheme (ABC SMC) routine.^28^ This iterative algorithm randomly samples parameter sets (5000) from their prior distributions and, through selecting those parameter sets that have the best goodness-of-fit to specified calibration data, it successively narrows the prior parameter distributions and improves the model's goodness-of-fit until a specific tolerance is reached. Data used to calibrate the model include multiple data measures over 2011–2019 and 2022 (just RNA prevalence) including: PWID population size; HCV seroprevalence in community, RNA positivity among those testing antibody-positive in community; incarceration and homelessness measures; OAT and NSP coverage; ORs for the association of homelessness or incarceration on HCV seroprevalence and other measures; and cumulative number of PWID HCV treated over 2015–2022 in prison, DTC and other settings. The 5000 parameter sets obtained through the model calibration were used as the baseline model fits for each ODN, which were used to produce the median and 95% credibility intervals (95% CrI; 2.5th–97.5th percentile range) for all model projections. Additional HCV antibody and RNA prevalence data from the UAM survey for 2020–2021 and antibody prevalence data from the needs assessment study (2022) were used for model comparison.

A detailed description of calibration algorithm, data sources, all model parameter priors and posteriors (Supplementary Tables 16 and 17) and calibration data measures (Supplementary Tables 15a–15h) for each region are given in the Supplementary Materials.

### Impact analysis

The baseline model was used to project the impact of the existing scale-up in testing and levels of linkage-to-treatment (termed the status quo or SQ scenario) on HCV incidence in each ODN from 2015 to end of 2023, and until 2030 if these same levels are maintained. The historical projections are important for determining the impact achieved with existing investment. Additional scenarios considered the impact of improving the HCV treatment pathway in settings that specifically target PWID from 2024, in line with guidelines^6^^,^^29^^,^^30^:

1. **Status quo**: Baseline model with testing rates and levels of linkage-to-treatment remaining unchanged after 2023;
2. **Increase testing in DTC**: From 2024, 80% of PWID attending DTC are tested annually^29^;
3. **Increase testing in prison**: From 2024, 75% of inmates are tested during prison stay^30^;
4. **Increase linkage in DTC**: From 2024, 80% of those diagnosed in DTC initiate treatment^6^;
5. **Increase linkage in prison**: From 2024, 80% of those diagnosed in prison initiate treatment.^6^

The treatment pathway was not improved in other settings, which do not specifically target PWID, because we wanted to focus on strategies that focus on PWID. The primary outcome was whether the overall HCV incidence among PWID in 2030 was <2/100pyrs. Other outcomes included the relative decrease in HCV incidence and RNA prevalence among PWID testing antibody-positive over 2015–2030.

### Cost-effectiveness analysis

We adopted a service provider perspective (providers of health care and harm reduction interventions for PWID, including testing and treatment) to evaluate the cost-effectiveness of existing and expanded HCV testing and treatment for PWID (2022 British pounds). Health care costs and utility weights for HCV disease, IDU and homelessness came from the literature (Supplementary Table 19).

We estimated the costs for different strategies of scaling up testing and treatment, including in DTC and prisons. This involved questionnaire-based interviews with people providing HCV testing in DTC and prisons to gain information on the activities and resources involved. Uncertainty bounds were developed for different costs based on variation in responses or applying ±20% bounds. Costs from the literature filled gaps in our estimations. Although the cost of HCV treatment is confidential, we have been advised that a cost between £3000 and £10,000 per person treated is realistic, so we assumed a cost of £10,000 per person treated in the status quo projections and £3000 in a sensitivity analysis.

The model estimated the incremental cost-effectiveness ratio (ICER) for continuing the SQ testing and treatment scenario in each region over 2016–2065 compared to a counterfactual scenario of no scale-up in testing and treatment from 2016. ICERs were estimated across the baseline model fits as the incremental costs divided by the incremental quality adjusted life years (QALYs) saved. Costs and utilities were discounted 3.5% annually. The mean ICER was compared to a willingness-to-pay threshold of £20,000 per QALY saved as recommended by the UK National Institute for Health and Care Excellence (NICE).^31^ We also estimated the ICER of any improved scenario that reached the incidence target, compared to continuing the SQ scenario over 2016–2065.

The costing and cost-effectiveness methods are described further in the Supplementary Materials pp 22–45.

### Sensitivity analyses

We undertook sensitivity analyses to assess the robustness of our SQ impact scenario. Because the SVR rates from the treatment database seem low (∼85%), we modelled a scenario with higher SVR rate (95%) from 2016.

A linear regression analysis of covariance (ANCOVA) was undertaken to determine which parameter uncertainties contributed most to variation in the projected HCV incidence in 2030 for our SQ scenario. The proportion of the model outcome's sum-of-squares contributed by each parameter was used to estimate the importance of individual parameters to the uncertainty.

We performed univariate sensitivity analyses to assess the robustness of the ICER for the SQ scenario. These included changing: time horizon to 25 years (baseline 50 years); stopping the SQ intervention in 2030 (SQ continues to 2065); changing annual discount rate to 0/5% (baseline 3.5%); lower (£3000) HCV treatment cost (baseline £10,000); and taking the lower utility value when combining utilities across domains^32^ instead of taking the product.

### Role of the funding source

The study sponsors had no involvement in the study design; in the collection, analysis, and interpretation of data; in the writing of the report; and in the decision to submit the paper for publication. The views expressed are those of the author and not necessarily those of the NIHR, the Department of Health and Social Care, or UKHSA.

## Results

### Treatment pathway data

**Fig. 2:**
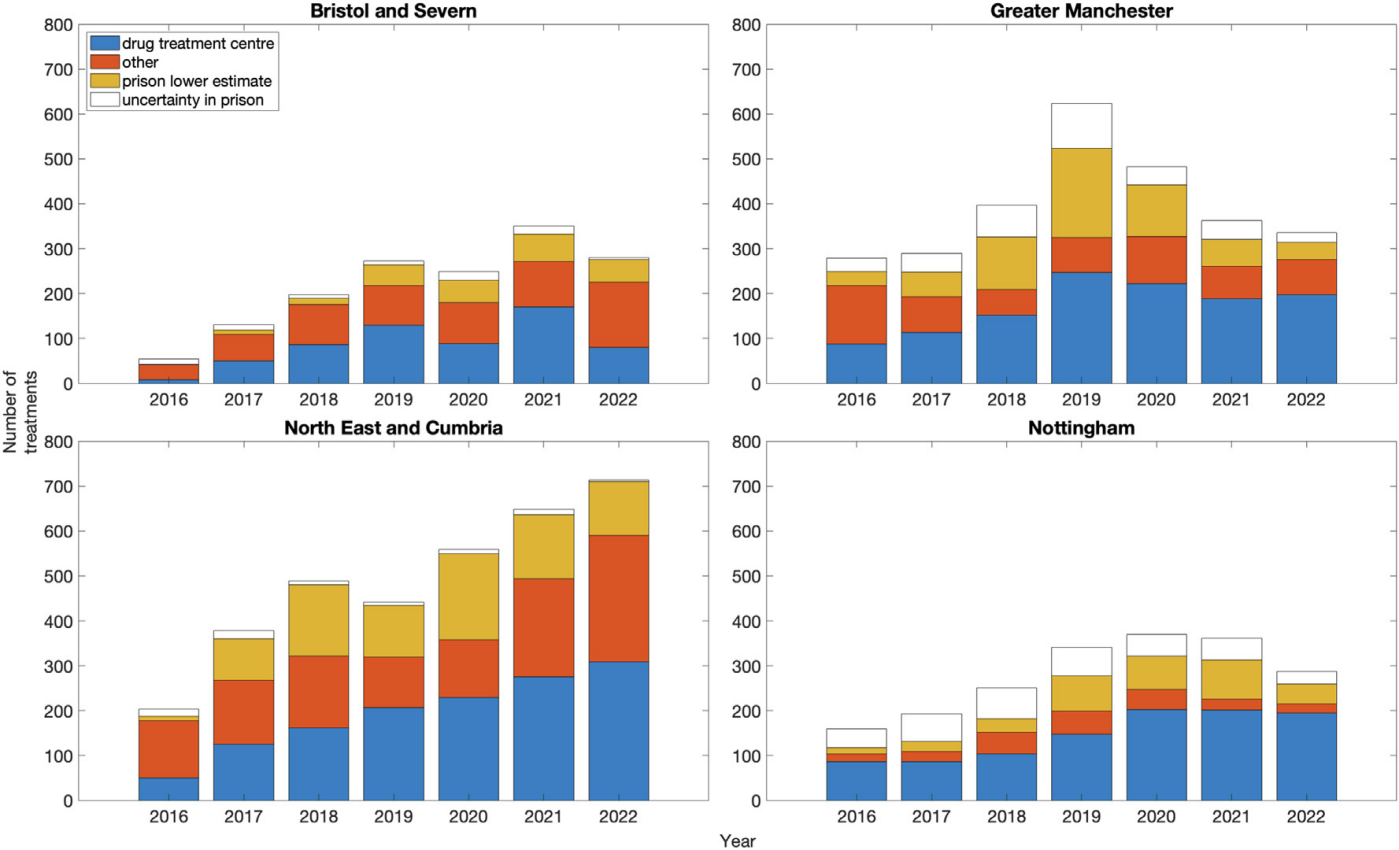
Estimated annual number of treatments among PWID referred through different settings for each ODN region over 2016–2022. All treatments referred from drug treatment centres are assumed to be among PWID, while we allow uncertainty in the number of prison-referred treatments that are among PWID, with the lower bound being those reported among PWID and upper bound being the total referred from prison.

Fig. 2 shows the scale-up in treatment among PWID for each ODN, with the overall annual number treated increasing 2.6-times over 2016–2021. Across our regions, greater scale-up in HCV treatment occurred in DTC (3.6-times) and prisons (7.8-times) than in other settings (1.7-times).

Table 2 shows that levels of linkage-to-treatment in prisons and DTC increased in 2015, but remained stable over 2015–2020 at 50–60%. Conversely, the time-to-treatment shortened in all settings from >1 year in 2015–2016 to <4 months in 2019–2020. The SVR rate increased from ∼50% to >80% between 2015 and 2016 due to the switch to using DAA therapies.

**Table 2:** Data on the cascade of care over time for different testing settings; estimated through the linkage of data from the HCV testing sentinel surveillance with the treatment database over 2016–2020.

	Pre 2015	2015–2016	2017–2018	2019–2020
**Prison**				
Percentage individuals linked to treatment following diagnosis	11.0% (9.9–12.2%)	52.1% (49.1–55.1%)	57.6% (55.7–59.5%)	58.2% (56.5–59.9%)
Time from diagnosis to starting treatment (days)	No data	870 (424–1349)	223 (80–535)	71 (32–212)
SVR rate	41.8% (36.3–46.4%)	83.8% (79.9–86.4%)	78.4% (76.2–80.6%)	73.8% (71.6–76.0%)
**Drug treatment Centers**				
Percentage individuals linked to treatment following diagnosis	10.4% (9.5–11.1%)	54.8% (52.7–56.8%)	58.9% (56.7–61.1)	52.3% (50.7–53.9%)
Time from diagnosis to starting treatment (days)	No data	776 (368–1271)	273 (111–575)	121 (51–290)
SVR rate	52.9% (48.6–57.2%)	81.3% (79.0–83.6%)	80.7% (78.3–83.1%)	74.1% (71.9–76.3%)
**Other testing settings**				
Percentage individuals linked to treatment following diagnosis	25.3% (24.7–25.8%)	52.4% (51.3–53.4%)	60.1% (58.9–61.3%)	55.0% (53.7–56.2%)
Time from diagnosis to starting treatment (days)	No data	525 (231–931)	174 (69–442)	83 (35–194)
SVR rate	49.6% (48.3–50.9%)	92.3% (91.5–93.1%)	89.0% (88.0–90.0%)	81.0% (79.5–82.5%)

We give the percentage (95% confidence interval) of people diagnosed with chronic HCV infection (test RNA-positive) that are linked to treatment, the median time in days (interquartile range) to start treatment for those linked to treatment, and the percentage (95% confidence interval) that achieve a sustained virological response (SVR; effective cure) for those that are treated and have an outcome recorded for SVR. We note that these estimates only included people with identifiers that allowed us to link between the databases. Note, follow up for monitoring linkage-to-treatment was truncated at the end of 2022, which may mean estimates for 2019–2020 are censored, and so prior ranges for % linked to treatment were extended to 90–95% - see Supplementary Tables 17a–17e.

### Status quo impact projections

The model calibration process improved the degree to which the model agreed with data (Supplementary Fig. 2) and narrowed most of the prior parameter ranges, as shown in Supplementary Fig. 3 and discussed in Supplementary Materials pp 47. However, some prior ranges are not narrowed as much, with this uncertainty being propagated to our model projections. This includes such things as the time to treatment, which has relatively narrow priors and some of the linkage-to-treatment parameters, which can take different values depending on the diagnosis rate.

**Fig. 3:**
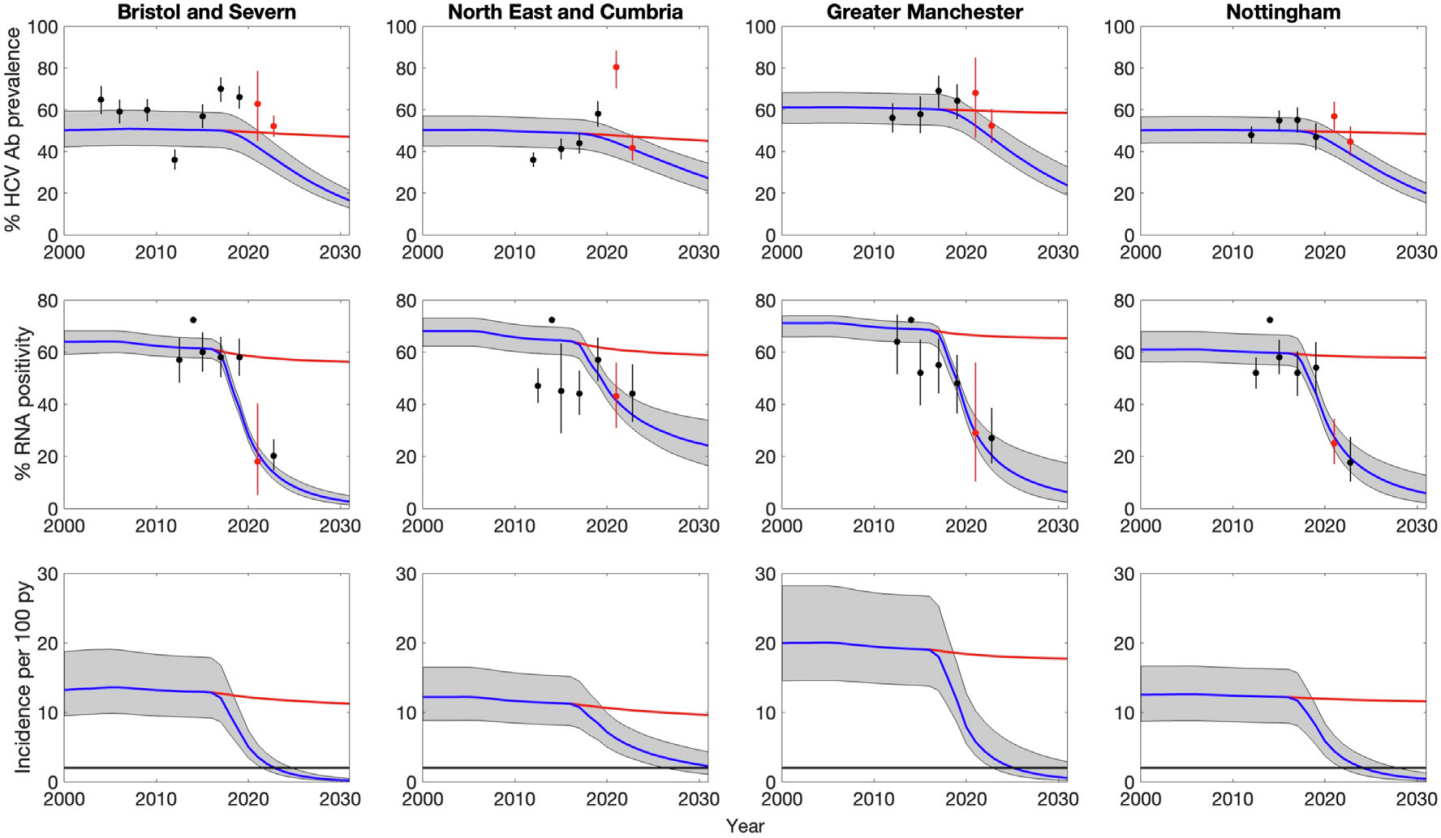
Comparison of model projections among community PWID over time for each ODN against available data for the HCV antibody prevalence (top row), HCV viraemia (HCV RNA) prevalence amongst those with a positive antibody response (middle row), and HCV incidence (bottom row). Projections are shown for the SQ scenario (blue line) and the counterfactual scenario (red line) where there was no scale up in treatment from 2016. Black points are antibody and RNA prevalence estimates that were used in the calibration, which come from the UAM survey (2011–2019), the needs assessment (2022) and sentinel surveillance (only RNA prevalence in 2014). Additional antibody prevalence estimates for Bristol come from three community surveys of PWID (2004–2009). Red points are HCV RNA and antibody prevalence estimates from the UAM survey for 2020–2021 and antibody prevalence estimates from the needs assessment (2022) that were not used in the model calibration.

Fig. 3 and Supplementary Figs. 4 and 5 show that the SQ model generally agrees well with available calibration data for each ODN. Exceptions include the RNA prevalence among those testing antibody-positive in Northeast and Cumbria before 2017, which is lower than other ODNs before the scale-up of DAAs. Additionally, the model agrees well with data from the UAM and needs assessment that was not fit to (red points; Fig. 3).

To reproduce the scale-up in treatment, our model projects that HCV testing increased 2.0–19.5 times in DTC from pre-2015 to 2017 (to 27–65% tested per year), 2.8–13.1 times in prison (to 53–70% tested per year) and 0.2–1.1 times in other settings (to 3–20% tested per year). The SQ model projects that the scale-up in treatment in each ODN resulted in smaller decreases in chronic HCV prevalence (RNA prevalence among PWID testing antibody-positive) than HCV incidence (Table 3). By end-2023, the SQ model projects that HCV incidence had decreased by 56.1% (95% CrI 42.4–68.5%) in Northeast and Cumbria up to 85.4% (95% CrI 78.7–90.5%) in Bristol and Severn, with the absolute HCV incidence ranging from 1.9/100pyrs (95% CrI 0.9–4.0) in Bristol and Severn to 4.9/100pyrs (95% CrI 2.5–8.8) in Northeast and Cumbria.

**Table 3:** Summary of status quo impact projections for each region over 2016–2023.

ODN region	RNA prevalence in PWID testing antibody positive			HCV incidence per 100pyrs		
	2016	2023	% change	2016	2023	% change
Bristol and Severn	61.4% (55.9–67.3%)	12.9% (8.9–17.6%)	79.0% (71.0–85.3%)	12.9 (8.0–21.1)	1.9 (0.9–4.0)	85.4% (78.7–90.5%)
Northeast and Cumbria	64.5% (57.1–71.0%)	35.3% (27.3–45.3%)	44.6% (32.0–57.3%)	11.3 (7.0–16.9)	4.9 (2.5–8.8)	56.1% (42.4–68.5%)
Greater Manchester	68.8% (59.4–72.1%)	19.5% (12.1–33.3%)	71.2% (51.4–82.2%)	19.1 (11.8–31.9)	3.3 (1.6–10.8)	81.7% (63.3–89.6%)
Nottingham	59.7% (52.8–70.1%)	18.6% (11.1–28.5%)	69.0% (53.6–81.6%)	12.2 (7.1–18.3)	2.6 (1.0–5.6)	78.4% (64.7–88.4%)

Ranges in brackets are 95% Credibility Intervals.

### Future impact projections

In the SQ scenario, the smallest relative decrease in HCV incidence over 2016–2030 is projected in Northeast and Cumbria (79.7%; 95% CrI 60.1–91.4%) and the greatest in Bristol and Severn (98.6%; 95% CrI 95.8–99.6%). HCV incidence is projected to be <2/100pyrs with >80% probability in Bristol and Severn (0.2/100pyrs; 95% CrI 0.0–0.8), Greater Manchester (0.5/100pyrs; 95% CrI 0.1–5.6) and Nottingham (0.4/100pyrs; 95% CrI 0.1–2.0), but is less likely (40.4% probability) in Northeast and Cumbria (2.2/100pyrs; 95% CrI 0.7–5.9; Table 4).

**Table 4:** Summary of impact projections for different intervention scenarios over 2016–2030.

ODN	Scenario from 2024	HCV incidence per 100pyrs		% of model runs reach threshold of 2 per 100pyrs by 2030
		2030	% change	
Bristol and Severn	Status quo	0.2 (0.0–0.8)	98.6% (95.8–99.6%)	100
	Increase testing in DTC	0.1 (0.0–0.4)	99.2% (97.5–99.8%)	100
	Increase testing in prison	0.1 (0.0–0.3)	99.4% (98.1–99.8%)	100
	Increase linkage in DTC	0.1 (0.0–0.6)	98.9% (96.8–99.6%)	100
	Increase linkage in prison	0.1 (0.0–0.6)	98.9% (96.7–99.7%)	100
Northeast and Cumbria	Status quo	2.2 (0.7–5.9)	79.7% (60.1–91.4%)	40.4
	Increase testing in DTC	0.7 (0.2–2.2)	93.3% (85.2–97.3%)	95.9
	Increase testing in prison	1.6 (0.5–4.6)	85.3% (69.8–94.2%)	66.2
	Increase linkage in DTC	1.8 (0.6–5.1)	83.2% (66.2–93.4%)	55.8
	Increase linkage in prison	2.0 (0.6–5.2)	82.3% (64.6–92.9%)	51.8
Greater Manchester	Status quo	0.5 (0.1–5.6)	97.0% (80.4–99.4%)	83.8
	Increase testing in DTC	0.1 (0.0–0.6)	99.7% (98.0–99.9%)	100
	Increase testing in prison	0.5 (0.1–4.8)	97.3% (82.3–99.4%)	86.4
	Increase linkage in DTC	0.6 (0.1–5.8)	96.7% (80.2–99.3%)	82.8
	Increase linkage in prison	0.7 (0.1–6.7)	96.1% (78.0–99.1%)	80.2
Nottingham	Status quo	0.4 (0.1–2.0)	96.6% (87.6–99.4%)	97.8
	Increase testing in DTC	0.1 (0.0–0.6)	99.1% (96.0–99.8%)	100
	Increase testing in prison	0.2 (0.0–1.2)	98.0% (92.2–99.6%)	99.9
	Increase linkage in DTC	0.3 (0.0–1.7)	97.3% (89.3–99.5%)	98.9
	Increase linkage in prison	0.4 (0.1–2.0)	96.3% (87.1–99.3%)	97.5

Range in brackets are 95% Credibility Intervals.

**Fig. 4:**
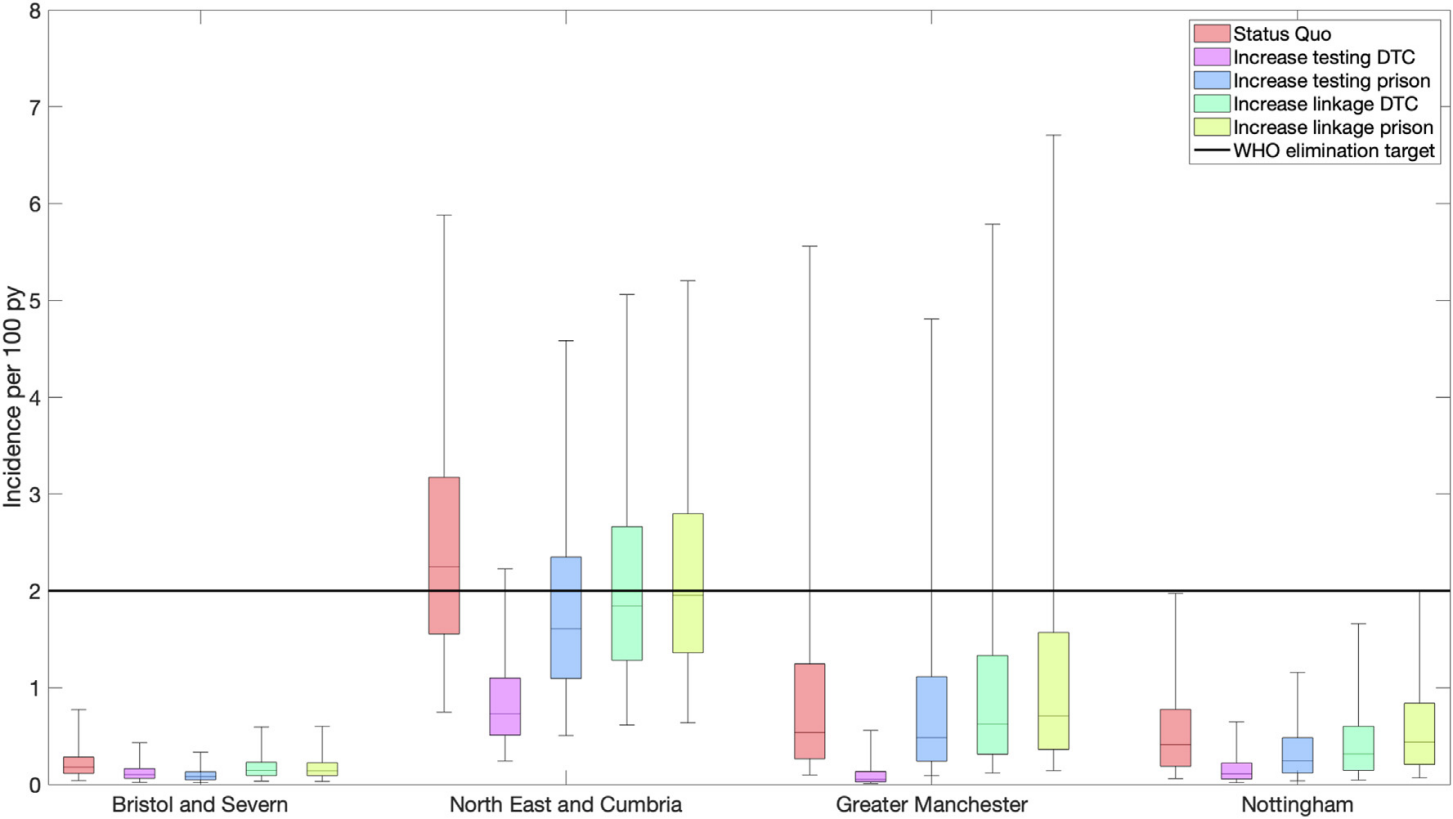
Model projections for incidence of HCV in 2030 for different intervention scenarios. Box plot whiskers are 2.5th and 97.5th percentiles, boxes are 25th–75th percentiles and horizontal line in each box shows the median. Black line is the 2 per 100 person-year incidence target for PWID populations set by the World Health Organization.

In our future scale-up scenarios, the proportion tested in a year in DTC increased from 27 to 65% (range in medians across regions) before 2024 to 80% from 2024 (scenario 2), while it increased from 53–70% to 78–97% in prisons (yearly proportion to ensure 75% tested during a prison sentence, scenario 3). The proportion linked-to-treatment in DTC increased from 70–76% before 2024 to 80% from 2024 (scenario 4), while it changed from 68–85% to 80% in prison (proportion linked-to-treatment did not always increase, scenario 5). Across these scenarios, our projections suggest the greatest decrease in incidence can be achieved through increasing testing in DTC (Fig. 4; Table 4). This increases the probability that HCV incidence becomes <2/100pyrs by 2030 to over 95% in Northeast and Cumbria and 100% in the other ODN. Other strategies (increasing testing in prisons or linkage-to-treatment in DTC and prisons) have less impact, but still increase the probability that HCV incidence becomes <2/100pyrs by 2030 to over 50% in Northeast and Cumbria. We did not consider the impact of combined intervention scenarios, with or without a reduced scale-up of each, because the single scenarios achieved elimination by themselves and increasing linkage-to-treatment achieved small additional impact.

### Cost-effectiveness analysis

Compared to no scale-up in HCV testing and treatment from 2016, we estimate the SQ scenario incurred an incremental cost ranging between £5,262,965 and £22,580,571 across the regions over 2016–2065 (Table 5), with most costs resulting from HCV treatment and HCV disease care (Supplementary Table 21). Across the regions, the SQ scenario will save 10,691–18,664 QALYs over 2016–2065, resulting in a mean ICER ranging between £308 and 1609/QALY saved, with all setting having a 100% chance of being cost-effective (Table 5) compared to the willingness-to-pay threshold (£20,000/QALY). Increasing testing in DTC from 2024 is cost-effective (£3918–17,549/QALY compared to SQ scenario) in all regions except Bristol and Severn (£77,014/QALY), while increasing testing in prisons is cost-effective in all regions (ranges from cost-saving to £1939/QALY saved). Seeing linkage-to-treatment rates were already high in the SQ scenarios, we only evaluated the cost-effectiveness of increasing linkage-to-treatment in Northeast and Cumbria, where it is shown to be highly cost-effective (£28–758/QALY).

**Table 5:** Cost-effectiveness of the status quo scenario (compared to the counterfactual) and intervention scenarios that either scale-up testing in DTC or prison from 2024 (compared to the status quo scenario) or improve linkage-to-treatment in DTC or prison from 2024 (compared to the status quo scenario; only Northeast and Cumbria because they only had demonstrable impact in that ODN region).

Scenario	Costs	Incremental cost compared to counterfactual	Incremental cost compared to Status quo	QALYs	Incremental QALYs compared to counterfactual	Incremental QALYs compared to Status quo	Mean ICER compared to Counterfactual	Mean ICER compared to Status quo	% of runs CE	% of runs cost-saving
**Bristol**										
Counterfactual	87,615,918			261,973						
Status quo	94,860,242	7,244,324		272,574	10,601		683		100^a^	16%^a^
Scaleup DTC testing from 2024	97,078,108		2,217,865	272,603		29		77,014	1	0%
Scaleup prison testing from 2024	95,252,895		392,653	272,777		203		1939	98	20%
**Northeast and Cumbria**										
Counterfactual	271,816,735			852,203						
Status quo	282,390,028	10,573,292		870,867	18,664		567		100^a^	30%^a^
Scaleup DTC testing from 2024	292,774,724		10,384,697	873,518		2651		3918	97	3%
Scaleup prison testing from 2024	282,303,767		−86,261	872,262		1395		−62	100	44%
Scaleup linkage in DTC from 2024	282,414,006		23,979	871,736		869		28	99	40%
Scaleup linkage in prison from 2024	282,847,983		457,955	871,471		604		758	98	36%
**Greater Manchester**										
Counterfactual	187,199,028			391,044						
Status quo	192,461,993	5,262,965		408,115	17,071		308		100%^a^	27%^a^
Scaleup DTC testing from 2024	196,690,230		4,228,237	408,590		475		8900	45%	8%
Scaleup prison testing from 2024	192,120,100		−341,893	408,202		86		−3953	98%	88%
**Nottingham**										
Counterfactual	94,936,261			351,173						
Status quo	117,516,832	22,580,571		365,204	14,031		1609		100%^a^	5%^a^
Scaleup DTC testing from 2024	121,214,848		3,698,017	365,415		211		17,549	43%	0%
Scaleup prison testing from 2024	117,540,601		23,769	365,618		414		57	100%	53%

All costs, QALYs, incremental costs and incremental QALY are mean values. Mean ICERs are the mean incremental costs divided by the mean incremental QALYs, although dividing numbers in table may not give the ICER in the table due to rounding errors.

a Percentage of runs cost-effective or cost-saving compared to the counterfactual; otherwise compared to status quo scenario.

### Sensitivity analyses

Assuming a higher SVR rate (95%) increases the probability of reaching the incidence target by 0–10 percentage points, with Northeast and Cumbria now having a 50.1% probability of decreasing HCV incidence to <2/100pyrs in the SQ scenario.

Analyses of covariance (Supplementary Table 20) indicate that uncertainty in the transmission risk when in community, homeless or prison and on OAT and NSP contributed most (35–58%) to the variability in the HCV incidence projections in 2030 for the SQ scenario in each ODN. Otherwise, only the diagnosis rate in DTC after 2017 plays an important role, especially in Greater Manchester where uncertainty in that parameter contributes 38% of the variability. Additionally, across the model fits for the four regions, we found that there was a strong association between the model projected RNA prevalence among those testing antibody positive in 2023 and the HCV incidence in 2030. This association can be seen in Supplementary Fig. 6, which shows that the probability of achieving an HCV incidence of <2/100pyrs is <22% if the RNA prevalence is >35% in 2023, 55% if the prevalence is 30–35%, and >86% if the prevalence is <30%.

In all sensitivity analyses, the ICER for the SQ scenario remained cost-effective, and could become cost-saving if there was no discount rate (42–100% probability across 4 regions) or if the HCV treatment cost was £3000 (22–59% probability; Supplementary Table 22).

## Discussion

HCV testing, linkage-to-care and HCV treatment among PWID at drug treatment centres (DTC) and prisons have all scaled up substantially since 2016, with testing increasing by up to 20-times, linkage-to-treatment increasing up to 5-times and time-to-treatment reducing by up to 90%. This scale-up in HCV testing and treatment is projected to have decreased HCV incidence among PWID by 56.1–85.4% over 2016–2023, with continuing current levels of testing and treatment being sufficient to achieve the WHO elimination target of decreasing HCV incidence to <2/100pyrs by 2030 in three regions (Bristol and Severn, Greater Manchester and Nottingham) but not Northeast and Cumbria. In this last region, a further scale-up of testing in DTC (80% of attendees tested annually) is likely to be the best strategy from 2024 to ensure that the elimination target is reached (with >95% probability). This strategy had more impact than increasing linkage-to-treatment because it resulted in a greater relative improvement in the treatment pathway, while it had more impact than increasing testing in prison because more PWID are in contact with DTC than are currently in prison. Economic analyses show that current levels of HCV testing and treatment are highly cost-effective as is increasing testing in DTC or prisons in the region that needs it to reach the elimination target.

A key strength of our analysis is that we calibrated the model in a Bayesian framework using detailed context-specific data and a tailor-made HCV transmission model that considers the cascade-of-care and inter-relationships between homelessness, prison and OAT. People with experience of HCV testing/treatment were involved in the work and in prioritizing our modelled strategies (Supplementary Materials pp 45).

There are, however, several limitations. First, some model parameters remain uncertain. For instance, the annual UAM survey had low power for each of our ODN regions, and so we had to pool data across multiple years to estimate parameters. Additionally, the UAM survey only sampled from some harm reduction settings in each region, and so it may not be generalisable to the whole region or all PWID. Nonetheless, other studies have found the UAM to be fairly comparable to other surveys using more representative sampling methods or larger samples.^33^

Second, there was uncertainty in our PWID population size estimates and therefore the proportion of PWID treated for HCV. As a result, we incorporated (±50%) into these size estimates which was propagated into our impact projections, but was not found to be important for determining uncertainty.

Third, HCV treatments were under-reported in the treatment database from 2020 and some treatments in PWID may have been missed or misclassified. We had to use prescription data for estimating numbers of treatments in 2020–2022, which we apportioned by injecting status and referral source using earlier data (2016–2020). This may have introduced bias. Because injecting drug use status is also likely to be under-reported, we assumed that all treatments in DTC were among PWID and allowed uncertainty in the number of treatments in prison that were among PWID. This was done following consultation with various stake-holders and because it resulted in the model generating a better fit with observed prevalence trends. In addition, data on the linkage-to-treatment for 2019–2020 had less follow-up time (2–3 years up to end of 2022) than estimates for earlier time periods (Table 2). Because this may mean we under-estimated the percentage linked-to-treatment, we allowed for higher values in our model calibration by increasing the upper bound of the prior distributions for this parameter (up to 90–95% linked-to-treatment) in 2019–2020. We also allowed large uncertainty bounds around the rate from diagnosis to treatment to account for any potential censoring of the data. Importantly, the posterior ranges for these parameters suggest that extending the bounds further would not have affected our model fits (Supplementary Fig. 3).

Fourth, our analysis assumed a steady coverage of harm reduction services (OAT and NSP) as coverage is already relatively high in England. Increasing and maintaining the coverage of harm reduction services will be important for achieving and maintaining elimination in other settings and could minimize re-infection in the UK.^5^

Lastly, our analysis evaluated 4/22 ODN regions in England,^12^ and so we cannot determine whether England and UK as a whole will reach the WHO incidence target. However, considering only 1 of our 4 modelled regions did not reach the target with existing levels of testing and treatment, we think that most England regions may be on target. A comparison of our regions with other ODN regions in England shows that they are fairly comparable (Supplementary Table 23) suggesting our results may be generalizable. If so, then our results suggest that if a region's RNA prevalence (among PWID testing antibody positive) in 2023 is <35% then that region is likely to be on target, whereas it is unlikely to be on target if the RNA prevalence is >35%. If not on target, then HCV testing in DTC should be scaled-up.

This analysis presents one of the first impact and cost-effectiveness analyses of an ongoing country-level HCV elimination initiative among PWID. Other models in the UK have projected that an achievable scale-up in HCV treatment could dramatically reduce HCV transmission,^34^ and that scaling up testing in DTC is cost-effective.^35^ Our analysis builds on these studies by using empirical data on the scale-up in treatment to assess impact and cost-effectiveness. Epidemiological analyses have also shown decreases in HCV chronic prevalence have occurred among PWID in England following the scale-up in HCV treatment^9^; consistent with our findings. Our analysis adds to these by evaluating the impact on HCV incidence, which is the key WHO elimination target. Elsewhere, models have projected that scaling up HCV treatment in harm reduction settings or prisons can considerably reduce HCV transmission among PWID,^35^^,^^36^ and some have estimated the testing needed to achieve this.^37^ However, to our knowledge, none have considered the testing and treatment required in different service settings and few have undertaken interim impact analyses^36^^,^^37^ or assessed the cost-effectiveness of achieving elimination.^38^

The widespread coverage of harm reduction interventions in England has enabled a large expansion of treatment. Unfortunately, most countries have much lower coverage of these services,^39^ and so will not be able to achieve the same level of treatment scale-up through these settings. Although country-level models have considered what is needed to achieve country-wide elimination,^37^ our analyses show that the impact of HCV treatment scale-up will be heterogeneous and so what is needed in different settings may vary.

Our findings suggest that existing testing and treatment strategies are likely to be sufficient to achieve the WHO HCV incidence targets in most regions of England. In regions where they are insufficient, a potential cost-effective strategy to ensure these targets are met is to increase testing in DTC (80% of attendees tested annually); something that could be determined by running a similar model in these regions. Our model projections also show that reductions in HCV incidence are tracked by reductions in viraemia, thus providing a proxy measure for assessing whether regions are on target.

Our analysis also highlights the importance of repeated bio-behavioural surveys for monitoring progress to achieving elimination, with our modelling particularly reliant on measures of RNA prevalence among people testing antibody-positive. These surveys need to be paired with detailed surveillance systems to track gaps and improvements in the cascade-of-care. Unfortunately, such datasets are unavailable in many settings, highlighting the need for elimination initiatives to invest in such data systems. These data systems will also be important for monitoring maintenance of elimination, with modelling being useful for determining what levels of interventions will keep transmission levels low.^40^

## Contributors

PV, ZW and MH conceived and designed the modelling study. ZW and HF developed the model and ZW performed all model analyses. PV and HF provided oversight on the model analyses. AT, RS, RH, LC and BE undertook data analyses for parameterising and calibrating the model. JK and AG supported the involvement of public contributors. PV and ZW wrote the initial draft of the manuscript. All authors contributed to guiding the overall analysis plan, interpreting interim and final results, and critically reviewing the final version of the manuscript.

## Data sharing statement

The model code and projections for this paper will be shared with interested parties upon reasonable request, which will be decided by Peter Vickerman and Zoe Ward.

## Declaration of interests

PV has received unrestricted research grants from Gilead not related to the submitted work. JV has received payments for chairing a Gilead sponsored meeting and sponsorship off ViiV to attend a European HIV conference. MD is a medical secretary of the UK Advisory Panel for Healthcare Workers Living with Bloodborne Viruses. GRF has received consulting fees off GSK, Gilead, BioMarin and CSL Behring; honoraria off Gilead, BioMarin and CSL Behring; and has Participated on Data Safety Monitoring Boards or Advisory Boards for GSK, Gilead, BioMarin and CSL Behring. MH is an unpaid trustee of the Society for Study of Addiction, and received support from the Viral Hepatitis Prevention Board to attend a meeting in Antwerp in 2024. This research was funded in whole, or in part, by a National Institute for Health Research Health Technology Health Assessment grant (NIHR128513) and the NIHR funded Health Protection Research Unit for Behavioural Science and Evaluation.
