# Supplementary material for: Impact and cost-effectiveness of scaling up HCV testing and treatment strategies for achieving HCV elimination among people who inject drugs in England: a mathematical modelling study
Source: Lancet Reg Health Eur. 2024 Dec 12;49:101176. doi: 10.1016/j.lanepe.2024.101176 (PMC11699733; doi:10.1016/j.lanepe.2024.101176)
Supplement: Supplementary Material [file mmc1.docx]

**Supplementary materials for paper titled:**

**Modelling the impact and cost-effectiveness of scaling up HCV case finding and treatment for achieving elimination among people who inject drugs in England**

**Authors:**

Ward Z^1^, Simmons R^2^, Fraser H^1,3^, Trickey A^1^, Kesten J^1,3,4^, Gibson A^5^, Reid L^6^, Cox S^6^, Gordon F^7^, Mc Pherson S^8^, Ryder S^9^, Vilar J^10^, Miners A^11^, Williams J^11^, Emmanouil B^12^, Desai M^2^, Coughlan L^2^, Harris R^2^, Foster GR^12^, Hickman M^1,3^, Mandal S^2^, Vickerman P^1,3^

^1^University of Bristol,

^2^UK Health Security Agency

^3^The National Institute for Health and Care Research (NIHR) Health Protection Research Unit (HPRU) in Behavioural Science and Evaluation, University of Bristol, Bristol, UK

^4^The National Institute for Health and Care Research Applied Research Collaboration West (NIHR ARC West) at University Hospitals Bristol and Weston NHS Foundation Trust, UK.

^5^ University of West of England,

^6^ The Hepatitis C Trust,

^7^University Hospitals Bristol,

^8^Newcastle Upon Tyne Hospitals NHS Foundation Trust,

^9^Nottingham University Hospitals NHS Trust,

^10^Manchester University NHS Foundation Trust.

^11^London School of Hygiene and Tropical Medicine,

^12^NHS England, UK

Table of Contents

[Detailed model description and schematics 3](#_Toc181383245)

[Model Equations 8](#_Toc181383246)

[Unlinked anonymous data used in this study 18](#_Toc181383247)

[Imputing injecting drug user status for treatments 19](#_Toc181383248)

[Sentinel Surveillance database and analyses 20](#_Toc181383249)

[Needs assessment survey 20](#_Toc181383250)

[Summary of cost-effectiveness methods 22](#_Toc181383251)

[Costing analysis of HCV testing strategies 24](#_Toc181383252)

[Methods 24](#_Toc181383253)

[Questionnaire Methods 24](#_Toc181383254)

[Identifying and inviting individuals to participate 24](#_Toc181383255)

[Questionnaire design 25](#_Toc181383256)

[Ethical approval 25](#_Toc181383257)

[How and when the questionnaire was administered 25](#_Toc181383258)

[Costing Analysis 25](#_Toc181383259)

[Literature review for costing analysis 26](#_Toc181383260)

[*Search strategy* 26](#_Toc181383261)

[*Literature review results* 26](#_Toc181383262)

[Costing results 28](#_Toc181383263)

[Questionnaire uptake 28](#_Toc181383264)

[Prison testing 28](#_Toc181383265)

[High intensity test and treat (HITT) sessions in prison 31](#_Toc181383266)

[Drug treatment centre testing 32](#_Toc181383267)

[Other Setting 34](#_Toc181383268)

[Test costs 34](#_Toc181383269)

[Healthcare staff costs 35](#_Toc181383270)

[Copy of Questionnaire 37](#_Toc181383271)

[Patient and public involvement 45](#_Toc181383272)

[Model Calibration Algorithm 47](#_Toc181383273)

[Supplementary data tables for model parameterization and calibration 49](#_Toc181383274)

[Supplementary Modelling Results 69](#_Toc181383275)

[Supplementary Calibration figures 69](#_Toc181383276)

[Ancova Analysis 78](#_Toc181383277)

[Cost breakdown 79](#_Toc181383278)

[Cost Effectiveness Sensitivity analysis 80](#_Toc181383279)

[Comparison to other ODN regions 82](#_Toc181383280)

[References 84](#_Toc181383281)

## Detailed model description and schematics

We developed a dynamic compartmental model of HCV transmission, testing and treatment among people who inject drugs (PWID) to evaluate the impact of current case finding and treatment interventions in 4 operational delivery network (ODN) regions in England. The population was stratified by

- harm reduction intervention access (none i=1, OAT i=2, NSP i=3, OAT + NSP i=4),
- homelessness status (never homeless j=1, currently homeless j=2, previously homeless j=3),
- incarceration status (never incarcerated k=1, currently incarcerated k=2, previous recent incarceration in last 6 months k=3, and previous non-recent incarceration k=4),
- disease status (metavir state F0 to F3 m=1..4, compensated cirrhosis m=5, decompensated cirrhosis m=6, hepatocellular carcinoma (HCC) m=7, transplant m=8 and post-transplant m=9),
- infection, testing and treatment pathway (susceptible $S_{i,j}^{k,m}$, infected antibody (Ab)+ve and RNA+ve $I_{i,j}^{k,m}$, exposed but not infected Ab+ve RNA-ve $E_{i,j}^{k,m}$, infected and diagnosed $D_{i,j}^{k,m}$, infected but lost to follow-up of HCV care $L_{i,j}^{k,m}$, on HCV treatment $T_{i,j}^{k,m}$, failed HCV treatment $F_{i,j}^{k,m}$, SVR Ab+ve RNA-ve $V_{i,j}^{k,m}$; with each variable representing the number of people in each state).
- Ex-injectors are included for the cost-effectiveness modelling as a stratification of the harm reduction intervention with i=0.

**Entry and exit of model**

The model is open, such that individuals continually enter through initiation of injecting drug use as susceptible (uninfected) PWID not on OAT or NSP, with a proportion of new PWID entering each incarceration ${(s}^{k})$ and homelessness compartment $\left( q_{j} \right)$, with the entry rate, $\vartheta$, set to balance the exit rate of PWID due to cessation and non-HCV deaths. In the absence of HCV, the size of the PWID population is assumed to be stable. Individuals leave the model either through non-HCV related death $\mu_{i}$, while chronically HCV-infected individuals in advanced stages of disease (decompensated cirrhosis onwards) experience an additional exit rate due to HCV related death $d_{m}$. Those starting or leaving OAT $\left( \epsilon,\varepsilon\right)$ and also those leaving prison $\xi_{i}$ have increased drug related mortality for a period of 4 weeks. PWID cease injecting at a rate $\nu$.

**Incarceration description**

Based on findings from previous modeling, we assume that individuals become incarcerated and re-incarcerated at different rates^1,2^, $\psi_{1}$and $\psi_{2}$, respectively, with re-incarceration for ex-injectors the same as incarceration rate and OAT lowering re-incarceration rates by a factor $r$. Release from prison is at a rate, $\rho$, independent of the number of previous incarcerations. Recent incarceration is defined as within the last 6 months. There is increased mortality within 4 weeks of release from prison $\xi_{i}$, dependent on OAT status within prison.

**Homelessness description**

Individuals in the model are assumed to become homeless for the first time at rate $\chi_{1}$ and the period of homelessness lasts $1/\kappa$ years. A proportion of individuals are assumed to be homeless upon release from prison, which depends on their homelessness status before being incarcerated. Upon leaving homelessness an individual enters the ever-homeless category. Re-entering current homeless category is at an increased rate $\chi_{2}$ compared to those who have never been homeless. Homelessness impacts on the duration of OAT with an increased leaving rate among those who are homeless $\gamma_{j}$. We assumed no effect of homelessness on NSP use.

**Harm reduction description**

PWID are enrolled onto OAT at a fixed rate $\beta^{k}$, which depends on their incarceration status (community (k=1,3,4) or prison (k=2)). A proportion $a,$ of community PWID are maintained on OAT when incarcerated or when released from prison. PWID who are enrolled onto OAT in the community or prison are assumed to leave OAT at a rate $\gamma_{j}$. OAT impacts on drug related mortality in multiple ways. Firstly, the first four weeks either on or off OAT is associated with an increased mortality risk $\varepsilon and \epsilon$, respectively, however the remaining time on OAT is associated with a decreased mortality risk ($u_{2}<\mu_{1})$. OAT is also associated with a reduced risk of incarceration or re-incarceration $(r<1)$. Being on OAT whilst incarcerated also confers lower mortality risk upon release $\xi_{i}$, regardless of whether they are retained on OAT after release.

NSP is only available in the community, so PWID are enrolled onto NSP at a fixed rate $\pi$ and stop using NSP at fixed rate $\varpi$. Upon incarceration all PWID on NSP cease using NSP. Upon release from prison a proportion will immediately access NSP $(b)$.

**Infection description**

All PWID can acquire and transmit HCV in their given setting, either community or in prison, with incarcerated PWID only being able to transmit HCV to other incarcerated PWID. Susceptible PWID are infected via injecting with a force of infection ${\Lambda_{i}A_{j}Z_{k}\phi}^{k}$, which is proportional to a settings’ dependent HCV transmission rate and the number of PWID and prevalence in each setting, where $\Lambda_{i}$, $A_{j}$ and $Z_{k}$ are the relative risks of transmission associated with harm reduction, homelessness and incarceration, respectively, and $\phi^{k}$is the baseline force of infection in community or prison. We assume that never incarcerated community PWID have an injecting transmission risk of $\lambda$, which is assumed to be the same for community PWID that have ever been incarcerated but not recently. We then assume that community PWID that have recently been incarcerated have an increased risk of HCV transmission $(\lambda\Omega)$ compared to those who have never been incarcerated. We also assume that currently incarcerated PWID have a different transmission risk $\left( \lambda\Psi\right)$ than never or previously incarcerated community PWID, but do not make any assumption that this is greater or less than for community PWID. Similar assumptions on transmission risk are made for the harm reduction interventions OAT $(\lambda B)$, NSP$(\lambda\Pi)$ or both OAT and NSP $(\lambda M)$ which decrease transmission risk compared to no intervention. We also assume current homelessness is associated with increased HCV transmission $(\lambda\Gamma)$ compared to never or ever homeless. We assume PWID sub-groups in the community mix proportionately relative to their overall transmission/acquisition risk (product of transmission risk and size of sub-group), and similarly for incarcerated PWID.

Following primary infection, a proportion of individuals, $\delta_{0}$, recover whilst the remainder become chronically infected. The proportion of individuals clearing subsequent infections, $\delta_{1},$ is higher than for primary infection^3,4^.

**HCV Testing and treatment description**

Following infection, individuals can be HCV tested at a rate ($\tau_{i}^{k}$), which is dependent upon the setting they are in, and following this they enter the diagnosed category. Each setting (drug treatment centre, prison, and testing in other settings) has a testing rate associated with it and these are summed for each individual. For example, if someone was accessing OAT (i.e., drug treatment centre), the testing rates for two settings (drug treatment centres and background) would be added together. Initiation on HCV treatment also occurs at a rate dependent on setting. The treatment rate depends on the proportion who commence treatment once diagnosed $\eta_{i}^{k}$ and the time to treatment for a setting $1/\omega_{i}^{k}$. Those who do not commence treatment within the time to treatment move to the lost to follow up category. Re-engagement with the treatment pathway occurs at a rate dependent on setting and is the same as the diagnosis rate post 2016. Treatment is assumed to be 12 weeks post-2016 (24 weeks pre-2016). Following HCV treatment, a proportion achieve a cure (sustained virological response; SVR), whilst the remainder enter the failed treatment category. We assume retreatment occurs at the same rate as initial treatment.

Re-infection of those who have attained SVR is possible with the same force of infection as primary infection.

**Disease progression description**

Upon primary infection, liver disease progression occurs as in Figure 1, with HCV-related death occurring from any stage after compensated cirrhosis. Disease progression stage does not impact on HCV transmission or treatment rate although most individuals with HCC and DC will be ex-injectors. Successful treatment is associated with a decrease in disease progression rates for those in F3 and compensated cirrhosis stages and cessation of progression in earlier disease stages; disease progression for stages after compensated cirrhosis is unaffected by successful treatment.

**Supplementary Figure 1** Model schematic

1. Incarceration and Homelessness (does not show cessation, mortality or initiation of injecting)


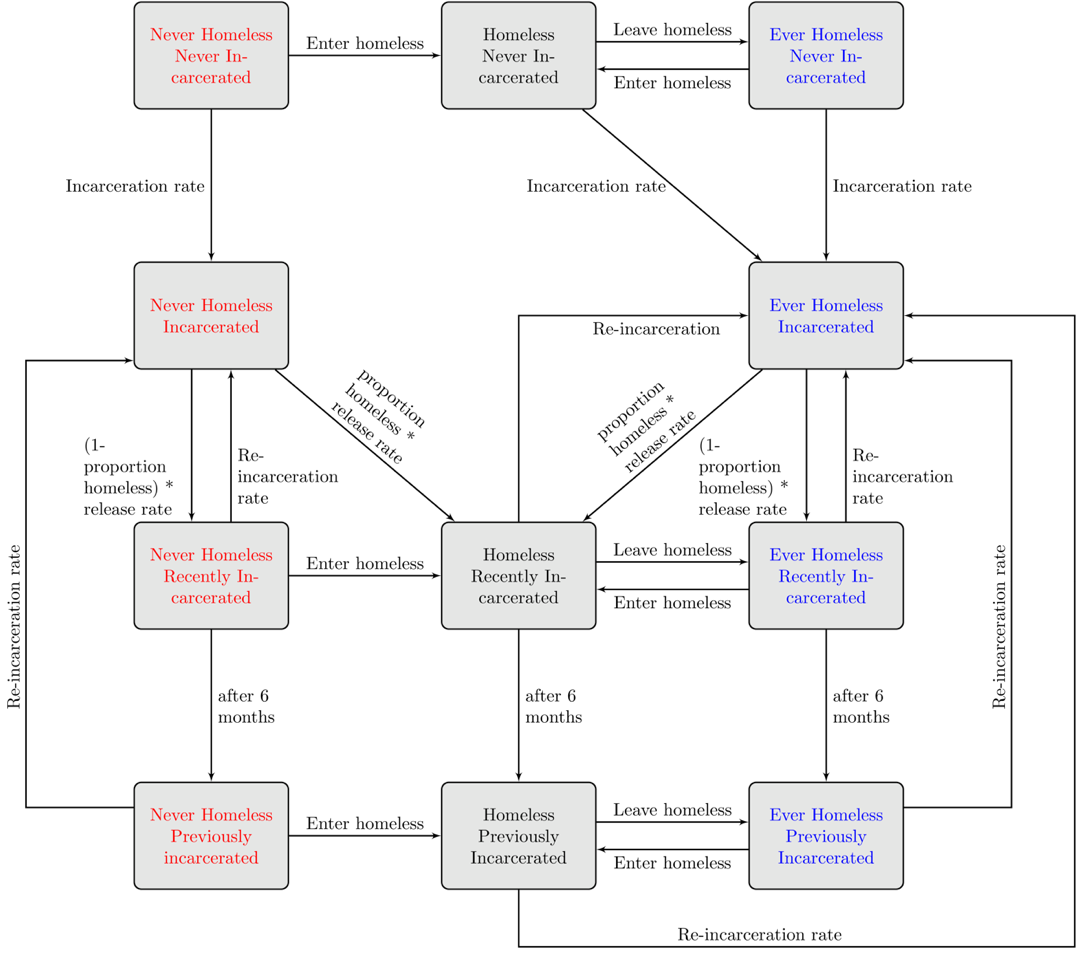


1. Harm Reduction status (does not show cessation, OAT impact on mortality and incarceration, mortality or initiation of injecting)


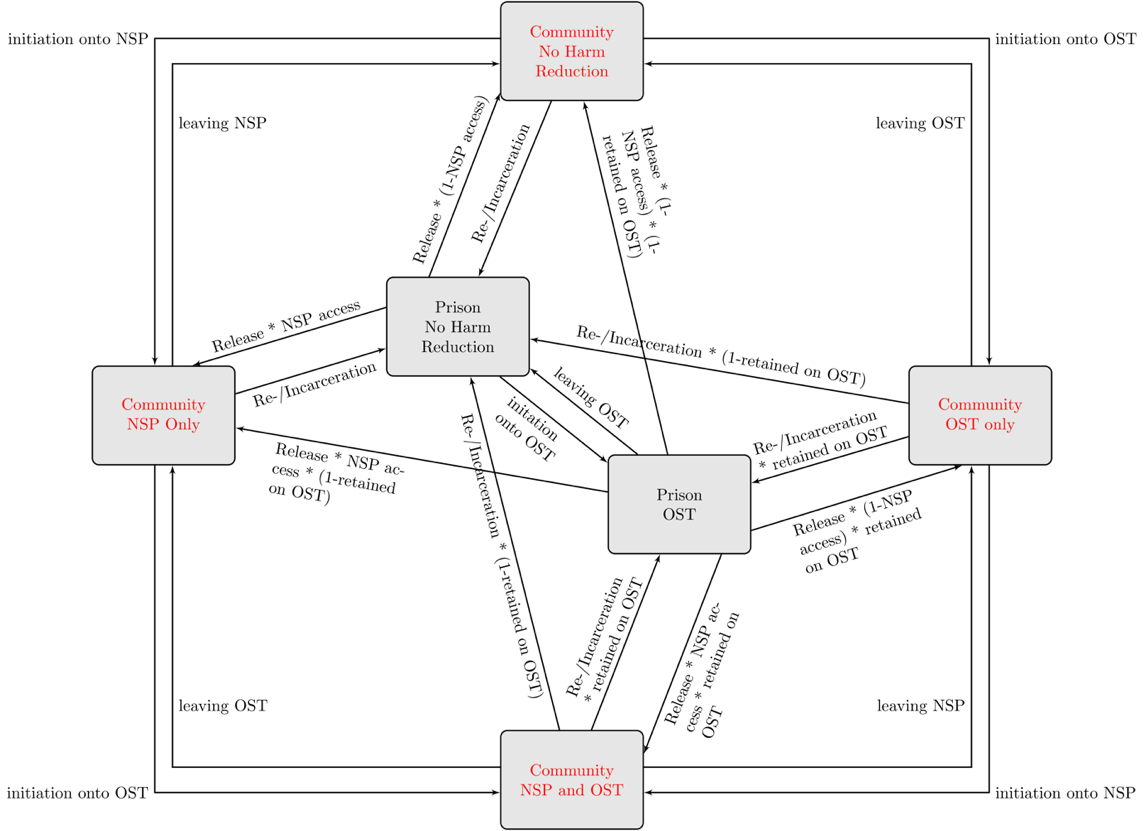


1. Infection and testing and treatment pathway (does not show cessation or mortality)


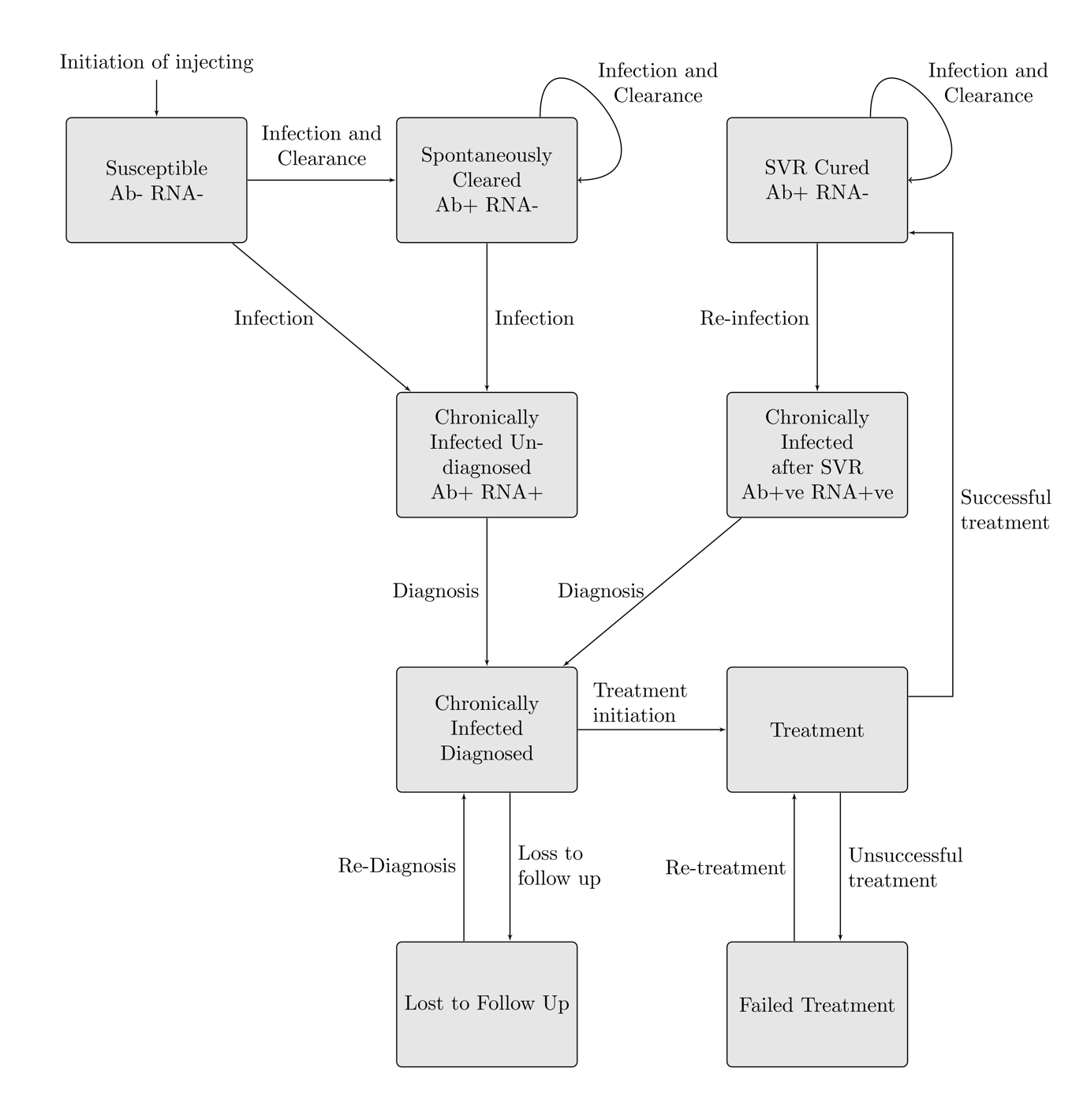


1. Disease progression in chronically infected individuals


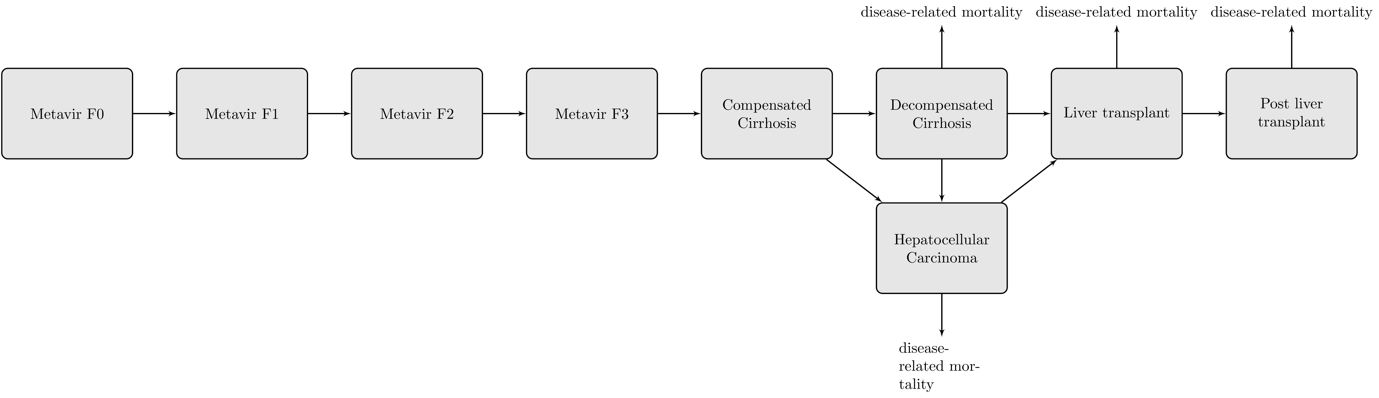


1. Disease progression in previously infected individuals with SVR


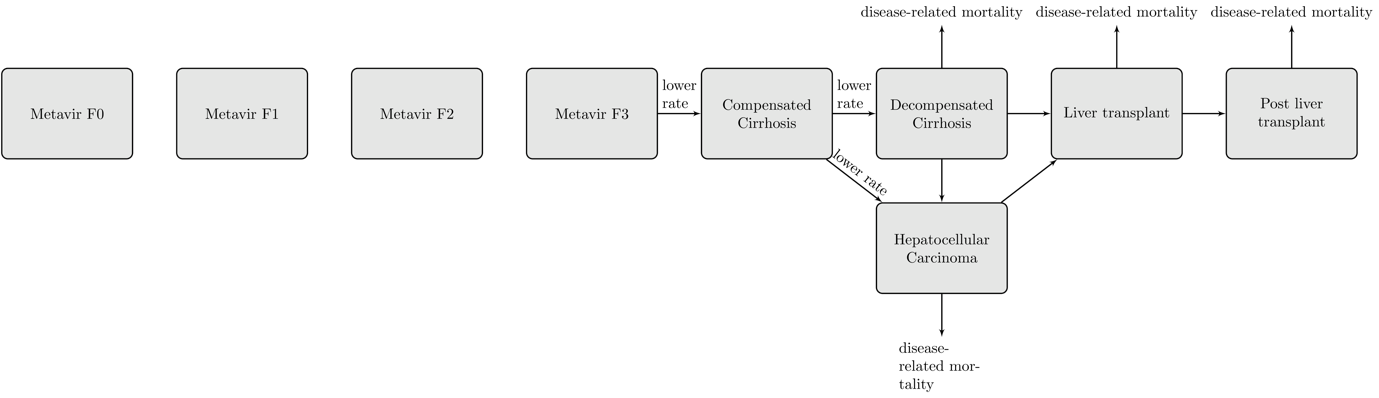


## Model Equations

The model is stratified by infection status, harm reduction status (i), homeless status (j), disease progression status (m) and incarceration status (k). The model equations are described in sub-models for each stratification.

**Infection and treatment sub-model**

This sub-model stands alone and can be used to investigate the impact of treatment on the prevalence of HCV in a population of people who inject drugs.

| Variable | Symbols |
| --- | --- |
| Susceptible individuals | $S_{i,j}^{k,m}$ |
| Exposed individuals (Ab+, RNA-) | $E_{i,j}^{k,m}$ |
| Chronically infected individuals (Ab+, RNA+) | $C_{i,j}^{k,m}$ |
| Diagnosed individuals | $D_{i,j}^{k,m}$ |
| Lost to follow up individuals | $L_{i,j}^{k,m}$ |
| Individuals undergoing Treatment | $T_{i,j}^{k,m}$ |
| Individuals who have attained SVR | $V_{i,j}^{k,m}$ |
| Individuals who have Failed treatment | $F_{i,j}^{k,m}$ |
| Individuals who have become re-infected after attaining SVR | $K_{i,j}^{k,m}$ |

| Parameters | Symbols | Units |
| --- | --- | --- |
| Infection rate | $\lambda$ | per year |
| Relative risk of transmission/acquisition of HCV when on OAT | $B$ | None |
| Relative risk of transmission/acquisition of HCV when homeless | $\Gamma$ | None |
| Relative risk of transmission/acquisition of HCV when using NSP | $\Pi$ | None |
| Relative risk of transmission/acquisition of HCV when recently released from prison | $\Omega$ | None |
| Relative risk of transmission/acquisition when in prison | $\Psi$ | None |
| Proportion of infections that spontaneously clear | $\delta_{p}$ | None, depends on whether individual is Ab-ve (p=0) or Ab+ve at infection (p=1) |
| Testing rate | $\tau_{i}^{k}(t)$ | Per year, depends on testing location (prison, OAT, other) and time |
| Proportion of diagnosed individuals treated within the current time to treatment | $\eta_{i}^{k}(t)$ | None, depends on location (prison, OAT, other) and time |
| Rate at which these individuals initiate treatment | $\omega_{i}^{k}(t)$ | Per year, depends on location (prison, OAT, other) and year |
| Length of time on treatment | $\sigma(t)$ | Years , depends on the year |
| Proportion of treatments that attain SVR | $\alpha_{i}^{k}\left( t \right)$ | None, depends on location (prison, OAT, other) and year |
| Non-HCV related death rate in PWID | $\mu_{i}$ | Per year, depends on OAT status |
| Rate of initiation of new injectors | $\theta_{j}^{k}$ | People per year  $\theta_{j}^{k}={\vartheta q}_{j}s^{k}$  Where $q_{j}$and $s^{k}$are proportions entering each homeless or incarceration state and $\sum_{j} q_{j}=1$ and $\sum_{k} s^{k}=1$ |

Notes: The testing rate depends on contact with drug treatment services (OAT), we assume that those on OAT $\left( i=2,4 \right),$ have an additional rate at which they are tested compared to those not on OAT. The diagnosis rate from Lost to follow up depends on time. At baseline we assume that $\tau_{i,k}=0$ for $t<2015$ for those in lost to follow up. The treatment rate does not depend on the current disease progression state.

The force of Infection

Define:

$$I_{i,j}^{k,m}=\sum C_{i,j}^{k,m}+D_{i,j}^{k,m}+L_{i,j}^{k,m}+K_{i,j}^{k,m}+F_{i,j}^{k,m}$$

which gathers together all of the infectious individuals in the population within the same harm reduction, incarceration and homeless status.

Define:

$$P_{i,j}^{k,m}=\sum S_{i,j}^{k,m}+E_{i,j}^{k,m}+C_{i,j}^{k,m}+D_{i,j}^{k,m}+L_{i,j}^{k,m}+K_{i,j}^{k,m}+T_{i,j}^{k,m}+V_{i,j}^{k,m}+F_{i,j}^{k,m}$$

Which gathers together all individuals in the population within the same harm reduction, incarceration and homeless status.

Define relative risk multipliers which depend on harm reduction, homeless and incarceration status where

$$\Lambda_{i}:\Lambda_{1}=1,\Lambda_{2}=B,\Lambda_{3}=\Pi,\Lambda_{4}=M,$$

$$A_{j}: A_{1}=1,A_{2}=\Gamma,A_{3}=1,$$

$$Z_{k}: Z_{1}=1,Z_{2}=\Psi,Z_{3}=\Omega,Z_{4}=1$$

The base force of infection in the community (k=1,3,4) is given by

$$\phi^{1,3,4}=\lambda\frac{\sum_{i,j,k=1,3,4,m} \Lambda_{i}A_{j}Z_{k}I_{i,j}^{k,m}}{\sum_{i,j,k=1,3,4,m} \Lambda_{i}A_{j}Z_{k}P_{i,j}^{k,m}}$$

The force of infection in prison (k=2) is given by

$$\phi^{2}=\lambda\frac{\sum_{i=1,2,j,m} {\Lambda_{i}I}_{i,j}^{2,m}}{\sum_{i=1,2,j,m} {\Lambda_{i}P}_{i,j}^{2,m}}$$

This allows the following system of equations for the infection part of the model (the dot above the letters denotes that these are differential equations with respect to time):

$$\dot{S}_{i,j}^{k,m}=\theta_{j}^{k}- \left( \mu_{i}+\nu+\Lambda_{i}A_{j}Z_{k}\phi^{k} \right)S_{i,j}^{k,m}$$

$$\dot{E}_{i,j}^{k,m}= \delta_{0}\Lambda_{i}A_{j}Z_{k}\phi^{k}S_{i,j}^{k,m}-\left( \mu_{i}+\nu+\left( 1-\delta_{1} \right)\Lambda_{i}A_{j}Z_{k}\phi^{k} \right)E_{i,j}^{k,m}$$

$$\dot{C}_{i,j}^{k,m}={\left( 1-\delta_{0} \right)\Lambda_{i}A_{j}Z_{k}\phi^{k}S}_{i,j}^{k,m}+\left( 1-\delta_{1} \right)\Lambda_{i}A_{j}Z_{k}\phi^{k}E_{i,j}^{k,m}-\left( \mu_{i}+\nu+\tau_{i}^{k} \right)C_{i,j}^{k,m}$$

$$\dot{D}_{i,j}^{k,m}=\tau_{i}^{k}\left( C_{i,j}^{k,m}+K_{i,j}^{k,m}+L_{i,j}^{k,m} \right)-\left( \mu_{i}+\nu+\omega_{i}^{k} \right)D_{i,j}^{k,m}$$

$$\dot{L}_{i,j}^{k,m}=(1-\eta_{i}^{t}){\omega_{i}^{k}D}_{i,j}^{k,m}-\left( \mu_{i}+\nu+\tau_{i}^{k} \right)L_{i,j}^{k,m}$$

$$\dot{T}_{i,j}^{k,m}={\eta_{i}^{t}\omega}_{i}^{k}\left( D_{i,j}^{k,m}+F_{i,j}^{k,m} \right)-\left( \mu_{i}+\nu+\sigma^{-1} \right)T_{i,j}^{k,m}$$

$$\dot{V}_{i,j}^{k,m}=\alpha_{i}^{k}{\sigma^{-1}T}_{i,j}^{k,m}-\left( \mu_{i}+\nu+\left( 1-\delta_{0} \right)\Lambda_{i}A_{j}Z_{k}\phi^{k} \right)V_{i,j}^{k,m}$$

$$\dot{F}_{i,j}^{k,m}=\left( 1-\alpha_{i}^{k} \right)\sigma^{-1}T_{i,j}^{k,m}-\left( \mu_{i}+\nu+{\eta_{i}^{t}\omega}_{i}^{k} \right)F_{i,j}^{k,m}$$

$$\dot{K}_{i,j}^{k,m}=\left( 1-\delta_{0} \right)\Lambda_{i}A_{j}Z_{k}\phi^{k}V_{i,j}^{k,m}-\left( \mu_{i}+\nu+\tau_{i}^{k} \right)K_{i,j}^{k,m}$$

When ex-injectors are included in the model $(k=1)$, there are no new infections in ex-injectors, but they can be diagnosed and treated as for current injectors (in prison or in other settings but not via drug treatment centres (OAT)).

**Demographics sub-model**

The letter $S_{i,j}^{k,m}$ denotes a susceptible individual. The subscript i is from 1,2,...4 and denotes harm reduction status (none, OAT only, NSP only, OAT and NSP). The subscript j is either 1,2, or 3 and denotes homelessness status (never, current, ever). The superscript k is from 1,2,...4 denoting incarceration status (never, incarcerated, recently incarcerated, ever). The superscript m is from 1,2,..9 denoting the disease progression state (more on this sub-model below).

| Parameter | Symbol | Units |
| --- | --- | --- |
| Transition rate from off OAT to on OAT | $\beta^{k}$ | Per year, different rate for community (k=1,3,4) and prison (k=2) |
| Transition rate from on OAT to off OAT | $\gamma_{j}^{k}$ | Per year, different rates for community (k=1,3,4), prison (k=2) and homeless (j=2, overides the community rate) |
| Transition rate from off NSP to on NSP | $\pi$ | Per year |
| Transition rate from on NSP to off NSP | $\varpi$ | Per year |
| Transition rate from never homeless to homeless | $\chi_{1}$ | Per year |
| Transition rate from homeless to ever homeless | $\kappa$ | Per year |
| Transition rate from ever homeless to homeless | $\chi_{2}$ | Per year |
| Incarceration rate from never to current incarceration | $\psi_{1}$ | Per year |
| Release rate from current incarceration to recent incarceration | $\rho$ | Per year |
| Re-incarceration rate from recent or ever incarcerated to current incarceration | $\psi_{2}$ | Per year |
| Proportion retained on OAT on incarceration or upon release from prison | $a$ | None |
| Proportion in contact with NSP upon release from prison | $b$ | None |
| Proportion of those never homeless before who are not homeless upon release from prison | $c$ | None |
| Proportion of those ever homeless before who are not homeless upon release from prison | $f$ | None |
| Factor reduction in incarceration rates when on OAT | $r$ | None |
| Mortality associated with release from prison | $\xi_{i}$ | Per year |
| Mortality associated with first four weeks after starting OAT | $\varepsilon$ | Per year |
| Mortality associated with first four weeks after discontinuing OAT | $\epsilon$ | Per year |
| Injecting cessation rate | $\nu$ | Per year |

The terms for the differential equations for this part of the model are given by the following sub-model where the terms concerning movement between OAT, NSP homeless and incarceration states for variable $S_{i,j}^{k,m}$and are valid for all infection sub-model states (different variable letters) and disease progression states (superscript m).

**For those off OAT and NSP**

$$\dot{S}_{1,1}^{1,m}=\theta_{1}^{1}+{\left( 1-\epsilon\right)\gamma}_{1}^{1}S_{2,1}^{1,m}+\varpi S_{3,1}^{1,m}-\left( \mu_{1}+\nu+\beta^{1}+\pi+\chi_{1}+\psi_{1} \right)S_{1,1}^{1,m}$$

$$\dot{S}_{1,2}^{1,m}=\theta_{2}^{1}+{\left( 1-\epsilon\right)\gamma}_{2}^{1}S_{2,2}^{1,m}+\varpi S_{3,2}^{1,m}+\chi_{1}S_{1,1}^{1,m}+\chi_{2}S_{1,3}^{1,m}-\left( \mu_{1}+\nu+\beta^{1}+{\pi+\psi}_{1}+\kappa\right)S_{1,2}^{1,m}$$

$$\dot{S}_{1,3}^{1,m}=\theta_{3}^{1}+{\left( 1-\epsilon\right)\gamma}_{3}^{1}S_{2,3}^{1,m}+\varpi S_{3,3}^{1,m}+\kappa S_{1,2}^{1,m}-\left( \mu_{1}+\nu+\chi_{2}+\psi_{1}+\beta^{1}+\pi\right)S_{1,3}^{1,m}$$

$$\dot{S}_{1,1}^{3,m}=\theta_{1}^{3}+{\left( 1-\epsilon\right)\gamma}_{1}^{3}S_{2,1}^{3,m}+\varpi S_{3,1}^{3,m}+\rho\left( 1-b \right)\left[ \left( 1-\xi_{0} \right)c\left( S_{1,1}^{2,m}+S_{3,1}^{2,m} \right)+c\left( 1-\xi_{1} \right)\left( 1-a \right)\left( S_{2,1}^{2,m}+S_{4,1}^{2,m} \right) \right]-\left( \mu_{1}+\nu+\chi_{1}+\beta^{1}+\pi+\psi_{2}+2 \right)S_{1,1}^{3,m}$$

$$\dot{S}_{1,2}^{3,m}=\theta_{2}^{3}+{\left( 1-\epsilon\right)\gamma}_{2}^{3}S_{2,2}^{3,m}+\varpi S_{3,2}^{3,m}+\chi_{1}S_{1,1}^{3,m}+\chi_{2}S_{1,3}^{3,m}+\rho\left( 1-b \right)\left( 1-\xi_{0} \right)\left[ \left( 1-c \right)\left( S_{1,1}^{2,m}+S_{3,1}^{2,m} \right)+\left( 1-f \right)\left( S_{1,3}^{2,m}+S_{3,3}^{2,m} \right) \right]+\rho\left( 1-b \right)\left( 1-a \right)\left( 1-\xi_{1} \right)\left[ \left( 1-c \right)\left( S_{2,1}^{2,m}+S_{4,1}^{2,m} \right)+\left( 1-f \right)\left( S_{2,3}^{2,m}+S_{4,3}^{2,m} \right) \right]-\left( \mu_{1}+\nu+\beta^{1}+\psi_{2}+\kappa+2+\pi\right)S_{1,2}^{3,m}$$

$$\dot{S}_{1,3}^{3,m}=\theta_{3}^{3}+{\left( 1-\epsilon\right)\gamma}_{3}^{3}S_{2,3}^{3,m}+\varpi S_{3,3}^{3,m}+\kappa S_{1,2}^{3,m}+\rho\left( 1-b \right)f\left( 1-\xi_{0} \right)\left( S_{1,3}^{2,m}+S_{3,3}^{2,m} \right)+\rho\left( 1-a \right)\left( 1-b \right)f\left( 1-\xi_{1} \right)\left( S_{2,3}^{2,m}+S_{4,3}^{2,m} \right)-\left( \mu_{1}+\nu+\beta^{1}+\psi_{2}+2+\pi+\chi_{2} \right)S_{1,3}^{3,m}$$

$$\dot{S}_{1,1}^{4,m}=\theta_{1}^{4}+{\left( 1-\epsilon\right)\gamma}_{1}^{4}S_{2,1}^{4,m}+\varpi S_{3,1}^{4,m}+2S_{1,1}^{3,m}-\left( \mu_{1}+\nu+\beta^{1}+\pi+\chi_{1}+\psi_{2} \right)S_{1,1}^{4,m}$$

$$\dot{S}_{1,2}^{4,m}=\theta_{2}^{4}+{\left( 1-\epsilon\right)\gamma}_{2}^{4}S_{2,2}^{4,m}+\varpi S_{3,2}^{4,m}+2S_{1,2}^{3,m}+\chi_{1}S_{1,1}^{4,m}+\chi_{2}S_{1,3}^{4,m}-\left( \mu_{1}+\nu+\beta^{1}+{\pi+\psi}_{2}+\kappa\right)S_{1,2}^{4,m}$$

$$\dot{S}_{1,3}^{4,m}=\theta_{3}^{4}+{\left( 1-\epsilon\right)\gamma}_{3}^{4}S_{2,3}^{4,m}+\varpi S_{3,3}^{4,m}+2S_{1,3}^{3,m}+\kappa S_{1,2}^{4,m}-\left( \mu_{1}+\nu+\chi_{2}+\psi_{2}+\beta^{1}+\pi\right)S_{1,3}^{4,m}$$

**For those on OAT but not NSP**

$$\dot{S}_{2,1}^{1,m}=\left( 1-\varepsilon\right)\beta^{1}S_{1,1}^{1,m}+\varpi S_{4,1}^{1,m}-\left( \mu_{2}+\nu+\gamma_{1}^{1}+\pi+\chi_{1}+{r\psi}_{1} \right)S_{2,1}^{1,m}$$

$$\dot{S}_{2,2}^{1,m}=\left( 1-\varepsilon\right)\beta^{1}S_{1,2}^{1,m}+\varpi S_{4,2}^{1,m}+\chi_{1}S_{2,1}^{1,m}+\chi_{2}S_{2,3}^{1,m}-\left( \mu_{2}+\nu+\gamma_{2}^{1}+{\pi+r\psi}_{1}+\kappa\right)S_{2,2}^{1,m}$$

$$\dot{S}_{2,3}^{1,m}=\left( 1-\varepsilon\right)\beta^{1}S_{1,3}^{1,m}+\varpi S_{4,3}^{1,m}+\kappa S_{2,2}^{1,m}-\left( \mu_{2}+\nu+\chi_{2}+{r\psi}_{1}+\gamma_{3}^{1}+\pi\right)S_{2,3}^{1,m}$$

$$\dot{S}_{2,1}^{3,m}=\left( 1-\varepsilon\right)\beta^{1}S_{1,1}^{3,m}+\varpi S_{4,1}^{3,m}+\rho\left( 1-b \right)\left[ ac\left( 1-\xi_{1} \right)\left( S_{2,1}^{2,m}+S_{4,1}^{2,m} \right) \right]-\left( \mu_{2}+\nu+\chi_{1}+\gamma_{1}^{3}+\pi+{r\psi}_{2}+2 \right)S_{2,1}^{3,m}$$

$$\dot{S}_{2,2}^{3,m}=\left( 1-\varepsilon\right)\beta^{1}S_{1,2}^{3,m}+\varpi S_{4,2}^{3,m}+\chi_{1}S_{2,1}^{3,m}+\chi_{2}S_{2,3}^{3,m}+a\rho\left( 1-b \right)\left( 1-\xi_{1} \right)\left[ \left( 1-c \right)\left( S_{2,1}^{2,m}+S_{4,1}^{2,m} \right)+\left( 1-f \right)\left( S_{2,3}^{2,m}+S_{4,3}^{2,m} \right) \right]-\left( \mu_{2}+\nu+\gamma_{2}^{3}+{r\psi}_{2}+\kappa+2+\pi\right)S_{2,2}^{3,m}$$

$$\dot{S}_{2,3}^{3,m}=\left( 1-\varepsilon\right)\beta^{1}S_{1,3}^{3,m}+\varpi S_{4,3}^{3,m}+\kappa S_{2,2}^{3,m}+a\rho\left( 1-b \right)f\left( 1-\xi_{1} \right)\left( S_{2,3}^{2,m}+S_{4,3}^{2,m} \right)-\left( \mu_{2}+\nu+\gamma_{3}^{3}+{r\psi}_{2}+2+\pi+\chi_{2} \right)S_{2,3}^{3,m}$$

$$\dot{S}_{2,1}^{4,m}=\left( 1-\varepsilon\right)\beta^{1}S_{1,1}^{4,m}+\varpi S_{4,1}^{4,m}+2S_{2,1}^{3,m}-\left( \mu_{2}+\nu+\beta^{1}+\pi+\chi_{1}+{r\psi}_{2} \right)S_{2,1}^{4,m}$$

$$\dot{S}_{2,2}^{4,m}=\left( 1-\varepsilon\right)\beta^{1}S_{1,2}^{4,m}+\varpi S_{4,2}^{4,m}+2S_{2,2}^{3,m}+\chi_{1}S_{2,1}^{4,m}+\chi_{2}S_{2,3}^{4,m}-\left( \mu_{2}+\nu+\gamma_{2}^{4}+{\pi+r\psi}_{2}+\kappa\right)S_{2,2}^{4,m}$$

$$\dot{S}_{2,3}^{4,m}=\left( 1-\varepsilon\right)\beta^{1}S_{1,3}^{4,m}+\varpi S_{4,3}^{4,m}+2S_{2,3}^{3,m}+\kappa S_{2,2}^{4,m}-\left( \mu_{2}+\nu+\chi_{2}+{r\psi}_{2}+\gamma_{3}^{4}+\pi\right)S_{2,3}^{4,m}$$

**For those on NSP but not OAT**

$$\dot{S}_{3,1}^{1,m}={\left( 1-\epsilon\right)\gamma}_{1}^{1}S_{4,1}^{1,m}+\pi S_{1,1}^{1,m}-\left( \mu_{1}+\nu+\beta^{1}+\varpi+\chi_{1}+\psi_{1} \right)S_{3,1}^{1,m}$$

$$\dot{S}_{3,2}^{1,m}={\left( 1-\epsilon\right)\gamma}_{2}^{1}S_{4,2}^{1,m}+\pi S_{1,2}^{1,m}+\chi_{1}S_{3,1}^{1,m}+\chi_{2}S_{3,3}^{1,m}-\left( \mu_{1}+\nu+\beta^{1}+{\varpi+\psi}_{1}+\kappa\right)S_{3,2}^{1,m}$$

$$\dot{S}_{3,3}^{1,m}={\left( 1-\epsilon\right)\gamma}_{3}^{1}S_{4,3}^{1,m}+\pi S_{1,3}^{1,m}+\kappa S_{3,2}^{1,m}-\left( \mu_{1}+\nu+\chi_{2}+\psi_{1}+\beta^{1}+\varpi\right)S_{3,3}^{1,m}$$

$$\dot{S}_{3,1}^{3,m}={\left( 1-\epsilon\right)\gamma}_{1}^{3}S_{4,1}^{3,m}+\pi S_{1,1}^{3,m}+\rho b\left[ \left( 1-\xi_{0} \right)c\left( S_{1,1}^{2,m}+S_{3,1}^{2,m} \right)+c\left( 1-\xi_{1} \right)\left( 1-a \right)\left( S_{2,1}^{2,m}+S_{4,1}^{2,m} \right) \right]-\left( \mu_{1}+\nu+\chi_{1}+\beta^{1}+\varpi+\psi_{2}+2 \right)S_{3,1}^{3,m}$$

$$\dot{S}_{3,2}^{3,m}={\left( 1-\epsilon\right)\gamma}_{2}^{3}S_{4,2}^{3,m}+\pi S_{1,2}^{3,m}+\chi_{1}S_{3,1}^{3,m}+\chi_{2}S_{3,3}^{3,m}+\rho b\left( 1-\xi_{0} \right)\left[ \left( 1-c \right)\left( S_{1,1}^{2,m}+S_{3,1}^{2,m} \right)+\left( 1-f \right)\left( S_{1,3}^{2,m}+S_{3,3}^{2,m} \right) \right]+\rho b\left( 1-a \right)\left( 1-\xi_{1} \right)\left[ \left( 1-c \right)\left( S_{2,1}^{2,m}+S_{4,1}^{2,m} \right)+\left( 1-f \right)\left( S_{2,3}^{2,m}+S_{4,3}^{2,m} \right) \right]-\left( \mu_{1}+\nu+\beta^{1}+\psi_{2}+\kappa+2+\varpi\right)S_{3,2}^{3,m}$$

$$\dot{S}_{3,3}^{3,m}={\left( 1-\epsilon\right)\gamma}_{3}^{3}S_{4,3}^{3,m}+\pi S_{1,3}^{3,m}+\kappa S_{3,2}^{3,m}+\rho\left( 1-b \right)f\left( 1-\xi_{0} \right)\left( S_{1,3}^{2,m}+S_{3,3}^{2,m} \right)+\rho b\left( 1-a \right)f\left( 1-\xi_{1} \right)\left( S_{2,3}^{2,m}+S_{4,3}^{2,m} \right)-\left( \mu_{1}+\nu+\beta^{1}+\psi_{2}+2+\varpi+\chi_{2} \right)S_{3,3}^{3,m}$$

$$\dot{S}_{3,1}^{4,m}={\left( 1-\epsilon\right)\gamma}_{1}^{4}S_{4,1}^{4,m}+\pi S_{1,1}^{4,m}+2S_{3,1}^{3,m}-\left( \mu_{1}+\nu+\beta^{1}+\varpi+\chi_{1}+\psi_{2} \right)S_{3,1}^{4,m}$$

$$\dot{S}_{3,2}^{4,m}={\left( 1-\epsilon\right)\gamma}_{2}^{4}S_{4,2}^{4,m}+\pi S_{1,2}^{4,m}+2S_{3,2}^{3,m}+\chi_{1}S_{3,1}^{4,m}+\chi_{2}S_{3,3}^{4,m}-\left( \mu_{1}+\nu+\beta^{1}+{\varpi+\psi}_{2}+\kappa\right)S_{3,2}^{4,m}$$

$$\dot{S}_{3,3}^{4,m}={\left( 1-\epsilon\right)\gamma}_{3}^{4}S_{4,3}^{4,m}+\pi S_{1,3}^{4,m}+2S_{3,3}^{3,m}+\kappa S_{3,2}^{4,m}-\left( \mu_{1}+\nu+\chi_{2}+\psi_{2}+\beta^{1}+\varpi\right)S_{3,3}^{4,m}$$

**For those on OAT and NSP**

$$\dot{S}_{4,1}^{1,m}=\left( 1-\varepsilon\right)\beta^{1}S_{3,1}^{1,m}+\pi S_{2,1}^{1,m}-\left( \mu_{2}+\nu+\gamma_{1}^{1}+\varpi+\chi_{1}+{r\psi}_{1} \right)S_{4,1}^{1,m}$$

$$\dot{S}_{4,2}^{1,m}=\left( 1-\varepsilon\right)\beta^{1}S_{3,2}^{1,m}+\pi S_{2,2}^{1,m}+\chi_{1}S_{4,1}^{1,m}+\chi_{2}S_{4,3}^{1,m}-\left( \mu_{2}+\nu+\gamma_{2}^{1}+{\varpi+r\psi}_{1}+\kappa\right)S_{4,2}^{1,m}$$

$$\dot{S}_{4,3}^{1,m}=\left( 1-\varepsilon\right)\beta^{1}S_{3,3}^{1,m}+\pi S_{2,3}^{1,m}+\kappa S_{4,2}^{1,m}-\left( \mu_{2}+\nu+\chi_{2}+{r\psi}_{1}+\gamma_{3}^{1}+\varpi\right)S_{4,3}^{1,m}$$

$$\dot{S}_{4,1}^{3,m}=\left( 1-\varepsilon\right)\beta^{1}S_{3,1}^{3,m}+\pi S_{2,1}^{3,m}+\rho abc\left( 1-\xi_{1} \right)\left( S_{2,1}^{2,m}+S_{4,1}^{2,m} \right)-\left( \mu_{2}+\nu+\chi_{1}+\gamma_{1}^{3}+\varpi+{r\psi}_{2}+2 \right)S_{4,1}^{3,m}$$

$$\dot{S}_{4,2}^{3,m}=\left( 1-\varepsilon\right)\beta^{1}S_{3,2}^{3,m}+\pi S_{2,2}^{3,m}+\chi_{1}S_{4,1}^{3,m}+\chi_{2}S_{4,3}^{3,m}+\rho ab\left( 1-\xi_{1} \right)\left[ \left( 1-c \right)\left( S_{2,1}^{2,m}+S_{4,1}^{2,m} \right)+\left( 1-f \right)\left( S_{2,3}^{2,m}+S_{4,3}^{2,m} \right) \right]-\left( \mu_{2}+\nu+\gamma_{2}^{3}+r\psi_{2}+\kappa+2+\varpi\right)S_{4,2}^{3,m}$$

$$\dot{S}_{4,3}^{3,m}=\left( 1-\varepsilon\right)\beta^{1}S_{3,3}^{3,m}+\pi S_{2,3}^{3,m}+\kappa S_{4,2}^{3,m}+a\rho bf\left( 1-\xi_{1} \right)\left( S_{2,3}^{2,m}+S_{4,3}^{2,m} \right)-\left( \mu_{2}+\nu+\gamma_{3}^{3}+{r\psi}_{2}+2+\varpi+\chi_{2} \right)S_{4,3}^{3,m}$$

$$\dot{S}_{4,1}^{4,m}=\left( 1-\varepsilon\right)\beta^{1}S_{3,1}^{4,m}+\pi S_{2,1}^{4,m}+2S_{4,1}^{3,m}-\left( \mu_{1}+\nu+\gamma_{1}^{4}+\varpi+\chi_{1}+{r\psi}_{2} \right)S_{4,1}^{4,m}$$

$$\dot{S}_{4,2}^{4,m}=\left( 1-\varepsilon\right)\beta^{1}S_{3,2}^{4,m}+\pi S_{2,2}^{4,m}+2S_{4,2}^{3,m}+\chi_{1}S_{4,1}^{4,m}+\chi_{2}S_{4,3}^{4,m}-\left( \mu_{2}+\nu+\gamma_{2}^{4}+{\varpi+r\psi}_{2}+\kappa\right)S_{4,2}^{4,m}$$

$$\dot{S}_{4,3}^{4,m}=\left( 1-\varepsilon\right)\beta^{1}S_{3,3}^{4,m}+\pi S_{2,3}^{4,m}+2S_{4,3}^{3,m}+\kappa S_{4,2}^{4,m}-\left( \mu_{2}+\nu+\chi_{2}+{r\psi}_{2}+\gamma_{3}^{4}+\varpi\right)S_{4,3}^{4,m}$$

**Prison**

$$\dot{S}_{1,1}^{2,m}=\theta_{1,1}^{2}+\left( 1-\epsilon\right)\gamma_{1}^{2}S_{2,1}^{2,m}+\psi_{1}S_{1,1}^{1,m}+\psi_{2}S_{1,1}^{3,m}+r\left( 1-a \right)\left( 1-\epsilon\right)\left( \psi_{1}S_{2,1}^{1,m}+\psi_{2}S_{2,1}^{3,m} \right)-\left( \mu_{2}+\nu+\beta^{2}+\rho\right)S_{1,1}^{2,m}$$

$$\dot{S}_{1,3}^{2,m}=\theta_{1,1}^{2}+\left( 1-\epsilon\right)\gamma_{1}^{2}S_{2,3}^{2,m}+\psi_{1}\left( S_{1,3}^{1,m}+S_{1,2}^{1,m} \right)+\psi_{2}\left( S_{1,3}^{3,m}+S_{1,2}^{3,m} \right)+\left( 1-\epsilon\right)\left( 1-a \right)r\psi_{1}\left( S_{2,3}^{1,m}+S_{2,2}^{1,m} \right)+{\left( 1-\epsilon\right)\left( 1-a \right)r\psi}_{2}\left( S_{2,3}^{3,m}+S_{2,2}^{3,m} \right)-\left( \mu_{2}+\nu+\beta^{2}+\rho\right)S_{1,3}^{2,m}$$

$$\dot{S}_{2,1}^{2,m}=\left( 1-\varepsilon\right)\beta^{2}S_{1,1}^{2,m}+a{r\psi}_{1}S_{2,1}^{1,m}+{ar\psi}_{2}S_{2,1}^{3,m}-\left( \mu_{1}+\nu+\beta^{2}+\rho\right)S_{2,1}^{2,m}$$

$$\dot{S}_{2,3}^{2,m}=\left( 1-\varepsilon\right)\beta^{2}S_{1,3}^{2,m}+ar\psi_{1}\left( S_{2,3}^{1,m}+S_{2,2}^{1,m} \right)+{ar\psi}_{2}\left( S_{2,3}^{3,m}+S_{2,2}^{3,m} \right)-\left( \mu_{2}+\nu+\beta^{2}+\rho\right)S_{2,3}^{2,m}$$

$$\dot{S}_{3,1}^{2,m}=\left( 1-\epsilon\right)\gamma_{1}^{2}S_{4,1}^{2,m}+\psi_{1}S_{3,1}^{1,m}+\psi_{2}S_{3,1}^{3,m}+r\left( 1-a \right)\left( 1-\epsilon\right)\left( \psi_{1}S_{4,1}^{1,m}+\psi_{2}S_{4,1}^{3,m} \right)-\left( \mu_{2}+\nu+\beta^{2}+\rho\right)S_{3,1}^{2,m}$$

$$\dot{S}_{3,3}^{2,m}=\left( 1-\epsilon\right)\gamma_{1}^{2}S_{4,3}^{2,m}+\psi_{1}\left( S_{3,3}^{1,m}+S_{3,2}^{1,m} \right)+\psi_{2}\left( S_{3,3}^{3,m}+S_{3,2}^{3,m} \right)+\left( 1-\epsilon\right)\left( 1-a \right)r\psi_{1}\left( S_{4,3}^{1,m}+S_{4,2}^{1,m} \right)+{\left( 1-\epsilon\right)\left( 1-a \right)r\psi}_{2}\left( S_{4,3}^{3,m}+S_{4,2}^{3,m} \right)-\left( \mu_{2}+\nu+\beta^{2}+\rho\right)S_{3,3}^{2,m}$$

$$\dot{S}_{4,1}^{2,m}=\left( 1-\varepsilon\right)\beta^{2}S_{3,1}^{2,m}+a{r\psi}_{1}S_{4,1}^{1,m}+{ar\psi}_{2}S_{4,1}^{3,m}-\left( \mu_{2}+\nu+\beta^{2}+\rho\right)S_{4,1}^{2,m}$$

$$\dot{S}_{4,3}^{2,m}=\left( 1-\varepsilon\right)\beta^{2}S_{3,3}^{2,m}+ar\psi_{1}\left( S_{4,3}^{1,m}+S_{4,2}^{1,m} \right)+{ar\psi}_{2}\left( S_{4,3}^{3,m}+S_{4,2}^{3,m} \right)-\left( \mu_{2}+\nu+\beta^{2}+\rho\right)S_{4,3}^{2,m}$$

**Ex-injector equations**

The testing and treatment equations are as above except that there are no new infections. Ex-injectors in our model have permanently ceased injecting and therefore no longer need OAT or NSP. They can be homeless or become incarcerated. Although we keep track of ever homeless and recent/ever incarceration it is not necessary for the treatment cascade. Ex-injector have i=0 to distinguish between current PWID not accessing harm reduction and ex-injectors who no longer need it.

| Parameter | Symbol | Units |
| --- | --- | --- |
| Transition rate from not homeless to homeless | $\chi_{1}$ | Per year |
| Transition rate from homeless to ever homeless | $\kappa$ | Per year |
| Incarceration rate | $\psi_{1}$ | Per year |
| Release rate from current incarceration to recent incarceration | $\rho$ | Per year |
| Proportion of those never homeless before who are not homeless upon release from prison | $c$ | None |
| Proportion of those ever homeless before who are not homeless upon release from prison | $f$ | None |
| Injecting cessation rate | $\nu$ | Per year |
| Mortality rate | $\mu_{j}^{*}$ | Depends on homeless status, if homeless have lower life expectancy |

$$\dot{S}_{0,1}^{1,m}=\nu\left( S_{1,1}^{1,m}+S_{2,1}^{1,m}+S_{3,1}^{1,m}+S_{4,1}^{1,m} \right)-\left( \mu_{1}^{*}+\chi_{1}+\psi_{1} \right)S_{0,1}^{1,m}$$

$$\dot{S}_{0,2}^{1,m}=\nu\left( S_{1,2}^{1,m}+S_{2,2}^{1,m}+S_{3,2}^{1,m}+S_{4,2}^{1,m} \right)+\chi_{1}S_{0,1}^{1,m}+\chi_{1}S_{0,3}^{1,m}-\left( \mu_{2}^{*}{+\psi}_{1}+\kappa\right)S_{0,2}^{1,m}$$

$$\dot{S}_{0,3}^{1,m}=\nu\left( S_{1,3}^{1,m}+S_{2,3}^{1,m}+S_{3,3}^{1,m}+S_{4,3}^{1,m} \right)+\kappa S_{0,2}^{1,m}-\left( \mu_{1}^{*}+\chi_{1}+\psi_{1} \right)S_{0,3}^{1,m}$$

$$\dot{S}_{0,1}^{2,m}=\nu\left( S_{1,1}^{2,m}+S_{2,1}^{2,m}+S_{3,1}^{2,m}+S_{4,1}^{2,m} \right)+\psi_{1}S_{0,1}^{1,m}+\psi_{1}S_{0,1}^{3,m}-\left( \mu_{1}^{*}+\rho\right)S_{0,1}^{2,m}$$

$$\dot{S}_{0,3}^{2,m}=\nu\left( S_{1,3}^{2,m}+S_{2,3}^{2,m}+S_{3,3}^{2,m}+S_{4,3}^{2,m} \right)+\psi_{1}\left( S_{0,3}^{1,m}+S_{0,2}^{1,m} \right)+\psi_{1}\left( S_{0,3}^{3,m}+S_{0,2}^{3,m} \right)-\left( \mu_{1}^{*}+\rho\right)S_{0,3}^{2,m}$$

$$\dot{S}_{0,1}^{3,m}=\nu\left( S_{1,1}^{3,m}+S_{2,1}^{3,m}+S_{3,1}^{3,m}+S_{4,1}^{3,m} \right)+\rho cS_{0,1}^{2,m}-\left( \mu_{1}^{*}+\chi_{1}+\psi_{1}+2 \right)S_{0,1}^{3,m}$$

$$\dot{S}_{0,2}^{3,m}=\nu\left( S_{1,2}^{3,m}+S_{2,2}^{3,m}+S_{3,2}^{3,m}+S_{4,2}^{3,m} \right)+{\chi_{1}S}_{0,1}^{3,m}+\chi_{1}S_{0,3}^{3,m}+\rho\left[ \left( 1-c \right)S_{0,1}^{2,m}+\left( 1-f \right)S_{0,3}^{2,m} \right]-\left( \mu_{2}^{*}+\psi_{1}+\kappa+2 \right)S_{0,2}^{3,m}$$

$$\dot{S}_{0,3}^{3,m}=\nu\left( S_{1,3}^{3,m}+S_{2,3}^{3,m}+S_{3,3}^{3,m}+S_{4,3}^{3,m} \right)+\kappa S_{0,2}^{3,m}+\rho fS_{0,3}^{2,m}-\left( \mu_{1}^{*}+\psi_{1}+2+\chi_{1} \right)S_{0,3}^{3,m}$$

$$\dot{S}_{0,1}^{4,m}=\nu\left( S_{1,1}^{4,m}+S_{2,1}^{4,m}+S_{3,1}^{4,m}+S_{4,1}^{4,m} \right)+2S_{0,1}^{3,m}-\left( \mu_{1}^{*}+\chi_{1}+\psi_{1} \right)S_{0,1}^{4,m}$$

$$\dot{S}_{0,2}^{4,m}=\nu\left( S_{1,2}^{4,m}+S_{2,2}^{4,m}+S_{3,2}^{4,m}+S_{4,2}^{4,m} \right)+2S_{0,2}^{3,m}+\chi_{1}S_{0,1}^{4,m}+\chi_{1}S_{0,3}^{4,m}-\left( \mu_{2}^{*}+\psi_{1}+\kappa\right)S_{0,2}^{4,m}$$

$$\dot{S}_{0,3}^{4,m}=\nu\left( S_{1,3}^{4,m}+S_{2,3}^{4,m}+S_{3,3}^{4,m}+S_{4,3}^{4,m} \right)+2S_{0,3}^{3,m}+\kappa S_{0,2}^{4,m}-\left( \mu_{1}^{*}+\chi_{1}+\psi_{1} \right)S_{0,3}^{4,m}$$

**Disease Progression states**

| State | Symbol Example |
| --- | --- |
| Metavir F0 | $C_{i,j}^{k,1}$ |
| Metavir F1 | $C_{i,j}^{k,2}$ |
| Metavir F2 | $C_{i,j}^{k,3}$ |
| Metavir F3 | $C_{i,j}^{k,4}$ |
| Metavir F4 (compensated cirrhosis) | $C_{i,j}^{k,5}$ |
| Decompensated cirrhosis | $C_{i,j}^{k,6}$ |
| Hepatocellular Carcinoma | $C_{i,j}^{k,7}$ |
| Liver Transplant | $C_{i,j}^{k,8}$ |
| Post Liver Transplant | $C_{i,j}^{k,9}$ |

| Parameter | Symbol |
| --- | --- |
| Yearly progression rate from f0 to f1 | $\zeta_{1}$ |
| Yearly progression rate from f1 to f2 | $\zeta_{2}$ |
| Yearly progression rate from f2 to f3 | $\zeta_{3}$ |
| Yearly progression rate from f3 to compensated cirrhosis | $\zeta_{4}$ |
| Yearly progression rate from compensated cirrhosis to decompensated cirrhosis | $\zeta_{5}$ |
| Yearly progression rate from compensated cirrhosis or decompensated cirrhosis to hepatocellular carcinoma | $\zeta_{6}$ |
| Yearly progression rate from decompensated cirrhosis or HCC to liver transplant | $\zeta_{7}$ |
| Yearly progression rate from liver transplant to post liver transplant | $\zeta_{8}$ |
| Decompensated cirrhosis related death rate per year | $\zeta_{6}$ |
| Hepatocellular carcinoma related death rate per year | $d_{7}$ |
| Liver transplant related death rate per year | $d_{8}$ |
| Post liver transplant related death rate per year | $d_{9}$ |
| Relative risk for progression rate from compensated to decompensated cirrhosis following SVR | $e_{5}$ |
| Relative risk for progression rate from compensated cirrhosis to HCC following SVR | $e_{6}$ |

These terms for the ordinary differential equations are concerned with movement through the disease states. Infection and treatment and demographics are described separately above. $DY_{i,j}^{k,m}$ denotes the terms in the ordinary differential equation of disease category $m$ for susceptible individuals who have previously been treated and $DC_{i,j}^{k,m}$ for infected individuals. These terms can be found in the equations for all values of $i,j,k$ and $m$.

$$\left( \begin{matrix} {DC}_{i,j}^{k,1} \\ {DC}_{i,j}^{k,2} \\ {DC}_{i,j}^{k,3} \\ {DC}_{i,j}^{k,4} \\ {DC}_{i,j}^{k,5} \\ {DC}_{i,j}^{k,6} \\ {DC}_{i,j}^{k,7} \\ {DC}_{i,j}^{k,8} \\ {DC}_{i,j}^{k,9} \end{matrix} \right)=\left( \begin{matrix} -\zeta_{1} & 0 & 0 & 0 & 0 & 0 & 0 & 0 & 0 \\ \zeta_{1} & -\zeta_{2} & 0 & 0 & 0 & 0 & 0 & 0 & 0 \\ 0 & \zeta_{2} & -\zeta_{3} & 0 & 0 & 0 & 0 & 0 & 0 \\ 0 & 0 & \zeta_{3} & -\zeta_{4} & 0 & 0 & 0 & 0 & 0 \\ 0 & 0 & 0 & \zeta_{4} & -\zeta_{5}-\zeta_{6} & 0 & 0 & 0 & 0 \\ 0 & 0 & 0 & 0 & \zeta_{5} & -\zeta_{6}-\zeta_{7}-d_{6} & 0 & 0 & 0 \\ 0 & 0 & 0 & 0 & \zeta_{6} & \zeta_{6} & -\zeta_{7}-d_{7} & 0 & 0 \\ 0 & 0 & 0 & 0 & 0 & \zeta_{7} & \zeta_{7} & -\zeta_{8}-d_{8} & 0 \\ 0 & 0 & 0 & 0 & 0 & 0 & 0 & \zeta_{8} & -d_{9} \end{matrix} \right)\left( \begin{matrix} C_{i,j}^{k,1} \\ C_{i,j}^{k,2} \\ C_{i,j}^{k,3} \\ C_{i,j}^{k,4} \\ C_{i,j}^{k,5} \\ C_{i,j}^{k,6} \\ C_{i,j}^{k,7} \\ C_{i,j}^{k,8} \\ C_{i,j}^{k,9} \end{matrix} \right)$$

$$\left( \begin{matrix} {DY}_{i,j}^{k,1} \\ {DY}_{i,j}^{k,2} \\ {DY}_{i,j}^{k,3} \\ {DY}_{i,j}^{k,4} \\ {DY}_{i,j}^{k,5} \\ {DY}_{i,j}^{k,6} \\ {DY}_{i,j}^{k,7} \\ {DY}_{i,j}^{k,8} \\ {DY}_{i,j}^{k,9} \end{matrix} \right)=\left( \begin{matrix} 0 & 0 & 0 & 0 & 0 & 0 & 0 & 0 & 0 \\ 0 & 0 & 0 & 0 & 0 & 0 & 0 & 0 & 0 \\ 0 & 0 & 0 & 0 & 0 & 0 & 0 & 0 & 0 \\ 0 & 0 & 0 & 0 & 0 & 0 & 0 & 0 & 0 \\ 0 & 0 & 0 & 0 & -e_{5}\zeta_{5}-{e_{6}\zeta}_{6} & 0 & 0 & 0 & 0 \\ 0 & 0 & 0 & 0 & e_{5}\zeta_{5} & -\zeta_{6}-\zeta_{7}-d_{6} & 0 & 0 & 0 \\ 0 & 0 & 0 & 0 & e_{6}\zeta_{6} & \zeta_{6} & -\zeta_{7}-d_{7} & 0 & 0 \\ 0 & 0 & 0 & 0 & 0 & \zeta_{7} & \zeta_{7} & -\zeta_{8}-d_{8} & 0 \\ 0 & 0 & 0 & 0 & 0 & 0 & 0 & \zeta_{8} & -d_{9} \end{matrix} \right)\left( \begin{matrix} V_{i,j}^{k,1} \\ V_{i,j}^{k,2} \\ V_{i,j}^{k,3} \\ V_{i,j}^{k,4} \\ V_{i,j}^{k,5} \\ V_{i,j}^{k,6} \\ V_{i,j}^{k,7} \\ V_{i,j}^{k,8} \\ V_{i,j}^{k,9} \end{matrix} \right)$$

**Incarceration sub-model used for calibration**

The incarceration sub-model (schematic below) was used to calculate the percentage ever incarcerated in the community and the average number of times that incarcerated or community PWID have been incarcerated by injecting duration to compare with data. This sub-model tracked each incarceration episode so that over time we can calculate the number of times prisoners have been incarcerated (schematic below). A cohort of 1000 injectors start in the model in one of the first 4 states (community never incarcerated, incarcerated, recently incarcerated and previously incarcerated once) and the model is run for 50 years.

To allow for the impact of OAT on re-/incarceration these rates in this model are attenuated using the following formula where $O$ is the coverage of OAT and r is the impact of OAT on incarceration rates:

$$\Psi_{i}^{*}=\left( Or+(1-O) \right)\Psi_{i}$$

Using the output for each year of injecting, the average number of incarcerations is calculated for those currently incarcerated or the community. The mean number of incarcerations for the injecting duration intervals required is calculated from this to compare to the data.

Community PWID are denoted by a variable $C_{n}^{p}$, where n is the number of times a person has been incarcerated and p is either 0,1 or 2 denoting never, recent or ever incarceration. PWID in prison are denoted by the variable $P_{n}$. The initial conditions for the cohort sub-model are influenced by parameters from the full model and an additional parameter which is the maximum number of incarcerations someone can have had before initiating injecting $n_{max}$. Where $s^{k}$is the proportion in each type of incarceration compartment $\sum_{k} s^{k}=1$, and the number that start in $C_{0}^{0}=1000s^{1}$. The number $1000s^{2}$ who start injecting in prison are shared among the first $n_{max}$ prison compartments. Similarly, those in recent and ever incarcerated categories are shared among the first $n_{max}$ community compartments. The ordinary differential equations for this sub-model are:

$$\dot{C_{0}^{0}}=-\left( \mu_{1}+\nu+\Psi_{1}^{*} \right)C_{0}^{0}$$

$$\dot{P_{1}=\Psi_{1}^{*}C_{0}-\left( \mu_{1}+\nu+\rho\right)P_{1}}$$

$$\dot{C_{1}^{1}}=\rho P_{1}-\left( \mu_{1}+\nu+\Psi_{2}^{*}+2 \right)C_{1}^{1}$$

$$\dot{C_{1}^{2}=2C_{1}^{1}-\left( \mu_{1}+\nu+\Psi_{2}^{*} \right)C_{1}^{2}}$$

$$\dot{P_{2}}=\Psi_{2}^{*}\left( C_{1}^{1}+C_{1}^{2} \right)-\left( \mu_{1}+\nu+\rho\right)P_{2}$$

$$\dot{C_{n}^{1}}=\rho P_{n}-\left( \mu_{1}+\nu+\Psi_{2}^{*}+2 \right)C_{n}^{1}$$

$$\dot{C_{n}^{2}}=2C_{n}^{1}-\left( \mu_{1}+\nu+\Psi_{2}^{*} \right)C_{n}^{2}$$

$$\dot{P_{n}}=\Psi_{2}^{*}\left( C_{(n-1)}^{1}+C_{(n-1)}^{2} \right)-\left( \mu_{1}+\nu+\rho\right)P_{n}$$

This continues up to n=29.

For n=30

$$\dot{C_{30}^{1}}=\rho P_{30}-\left( \mu_{1}+\nu+\Psi_{2}^{*}+2 \right)C_{30}^{1}$$

$$\dot{C_{30}^{2}}=2C_{30}^{1}-\left( \mu_{1}+\nu+\Psi_{2}^{*} \right)C_{30}^{2}$$

$$\dot{P_{30}}=\Psi_{2}^{*}\left( C_{29}^{1}+C_{29}^{2}+C_{30}^{1}+C_{30}^{2} \right)-\left( \mu_{1}+\nu+\rho\right)P_{30}$$

**Figure 2** Schematic **of** Incarceration sub-model


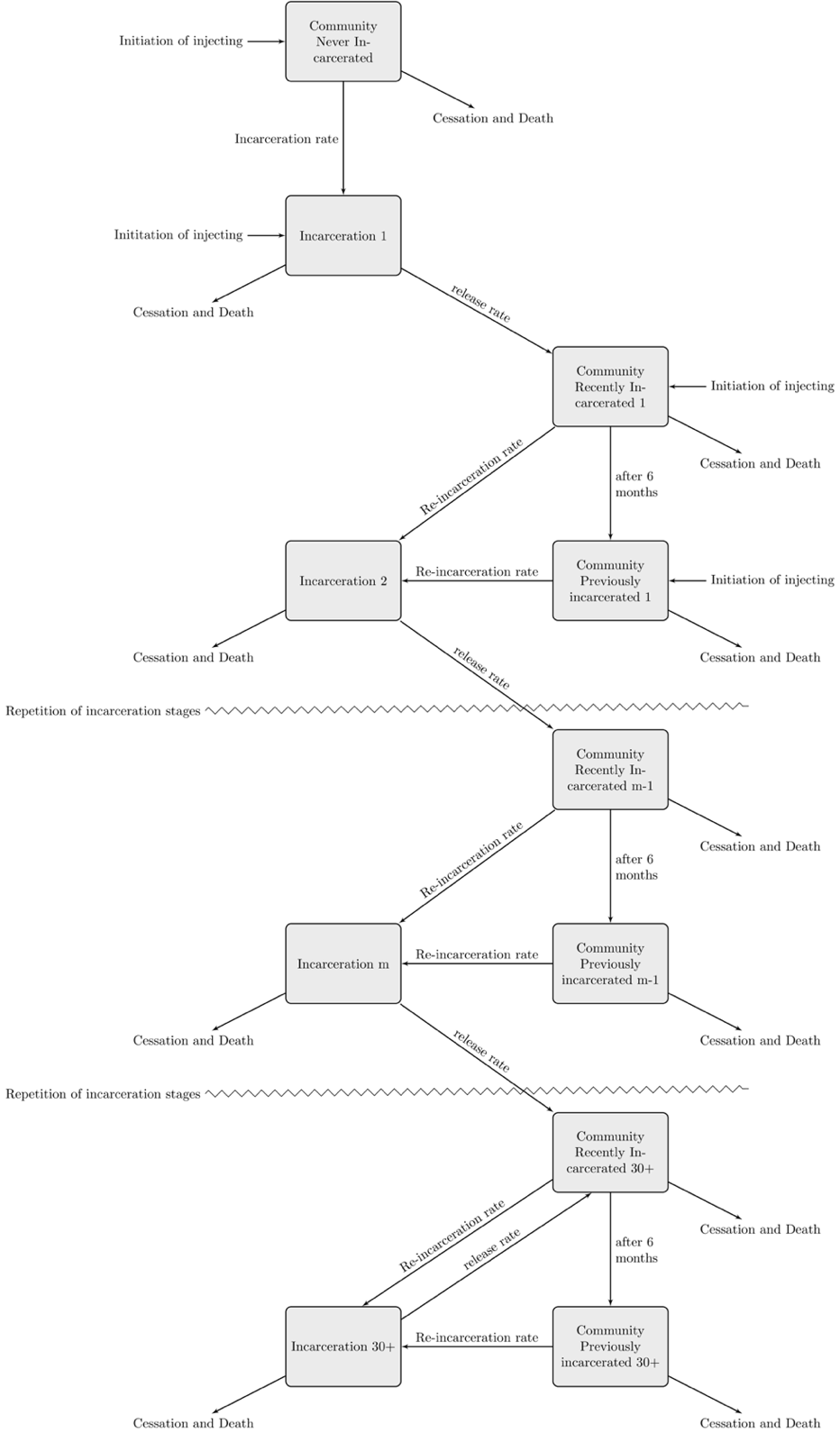


## Unlinked anonymous data used in this study

UAM data for multiple years was combined for the periods 2011-2013, 2014/2015, 2016/2017, and 2018/2019 to calculate most parameter and calibration estimates for each ODN. See sample sizes for each ODN region and time period in the table. To maximise precision, data across all available years of the UAM survey for each ODN were used to estimate various odds ratios (OR) used in the model calibration. These included: OR of being HCV antibody positive if ever incarcerated versus never incarcerated (2011-2019); OR of being HCV antibody positive if ever homeless versus never homeless PWID (2011-2019); OR of being homeless if recently incarcerated versus non-recently incarcerated (only 2017-2019; question on recent incarceration not included in earlier years); and OR of being on OAT if currently homelessness versus not currently homeless (2011-2019). Data for 2020/2021 was only used to produce prevalence estimates to compare to the model projections because of their small sample size.

**Table** Summary of sample sizes in the unlinked anonymous survey for each ODN region in our analyses and the overall sample used across these ODNs

|  | 2011-2013 | 2014/2015 | 2016/2017 | 2018/2019 | 2020/2021* | Overall |
| --- | --- | --- | --- | --- | --- | --- |
| Bristol and Severn | 383 | 305 | 254 | 300 | 107 | 1349 |
| Northeast and Cumbria | 720 | 393 | 507 | 289 | 166 | 2075 |
| Greater Manchester | 203 | 126 | 169 | 135 | 29 | 662 |
| Nottingham | 633 | 423 | 423 | 300 | 196 | 1869 |
| Overall | 1939 | 1247 | 1247 | 1024 | 498 | 5955** |

*These years had a smaller sample size because of the COVID-19 pandemic restricting the survey; these years were only used to produce estimates of antibody and RNA prevalence for model comparison and comparison with the needs assessment survey that had a larger sample size in 2022

**Overall sample size for 2011-2019 survey rounds was 5457 which are the survey rounds used to parameterize the model.

## Imputing injecting drug user status for treatments

Data on treatment in PWID were obtained from the Hepatitis C patient registry and treatment outcome system ([HCV treatment monitoring report 2018 (publishing.service.gov.uk)](https://assets.publishing.service.gov.uk/media/5bedb331e5274a084abc8474/HCV_treatment_monitoring_report_2018.pdf)) – the HCV treatment database. This includes data on age, sex, ethnicity, country of birth, source of referral and treatment setting, disease stage and injecting status (current, past, never). Injecting status has a high proportion of missing data (~20%), which was handled via an imputation procedure as there were comparatively small amounts of missing data in other variables (see table below). Multiple imputation chained equations were used, implemented in Stata’s mi: framework, using 10 imputed datasets. Age was treated as a continuous variable and imputed using linear regression; all other variables were categorical and imputed under logistic or multinomial logit models. All prediction equations included all other variables as predictors, plus year and ODN area. Data were augmented using a small number of pseudo-observations with low weights to avoid issues around perfect prediction ([[MI] Multiple Imputation (stata.com)](https://www.stata.com/manuals/mi.pdf)). The resulting imputed datasets were then aggregated to the level required for modelling inputs, and numbers averaged across the 10 imputed datasets.

**Table**: Summary of missing data for different variables in the treatment database

| **Variable** | **N missing** | **% missing** |
| --- | --- | --- |
| Age | 280 | 0.4% |
| Sex | 167 | 0.3% |
| Ethnicity | 4888 | 7.8% |
| COB | 8757 | 13.9% |
| Source of referral | 780 | 1.2% |
| Treatment setting | 673 | 1.1% |
| Disease stage | 1210 | 1.9% |
| Injecting drug use | 12655 | 20.1% |

One of the key assumptions to using MI is that data should be missing at random. However, it is sometimes thought that non-response to risk behaviours, such as injecting drug use status, is more likely if someone does not have this risk behaviour. We found injecting status to be most strongly associated with treatment setting and source of referral, which are largely complete (Table), and so we think this potential bias is largely avoided through the use of strong predictors of injecting status. For example, the odds ratio for current injecting vs. past/never injecting in those treated in drug treatment centres vs. secondary care is OR=7.4. Given the strength of these associations, and that only 20% are missing injecting status, it is likely that any unobserved covariates associated with injecting status that are not included in the imputation model (i.e., missing not at random effects) are unlikely to lead to any substantial differences in the derived total numbers of treated PWID.

## Sentinel Surveillance database and analyses

The UKHSA sentinel surveillance of blood borne virus testing (SSBBV) was used to estimate other parameters relating to the cascade-of-care for HCV. This surveillance dataset collates data on venous and dried blood spot testing for HCV in England through direct extracts from UKHSA/NHS laboratories, covering an estimated 40% of all tests undertaken among the GP registered population ^5,6^. The laboratories participating in SSBBV cover 50% of all prisons across England and includes two laboratories which are commissioned to undertake testing for the majority of drug services. The dataset collects demographic and testing data for all individuals tested for hepatitis C specific antibody (anti-HCV), indicative of current or past infection (“ever infected”), and HCV-RNA (indicative of viraemia, i.e., current infection) are extracted from participating laboratory information systems. Individuals are de-duplicated, and test results for each individual are linked over time using a combination of soundex code (a coding based on the person’s surname), first initial, date of birth and NHS number.

HCV testing data between 2015 and 2021 for persons ≥1 year old, were collated with subsequent HCV-RNA tests where available. Tests among persons aged <1 year were excluded due to the possibility of false-positive results from maternal antibodies, as were tests conducted through renal services and all reference laboratories as these tests are unlikely to reflect normal testing practises.

Through linking tests reported to SSBBV and treatment notification reported through the NHSE Arden & GEM patient registry of hepatitis C treatment, using NHS number and/or Name, date of birth and hospital number, we estimated the care pathway for a person. We describe the patient pathway as the proportion of diagnosed individuals (RNA-positive test result) who initiated treatment, the proportion of these who completed treatment and achieved SVR, and the time from diagnosis (RNA-positive test result) to initiating treatment (median and interquartile range). Due to small numbers in each ODN, this was estimated across all 22 ODN for three different time periods (2015-2016, 2017-2018 and 2019-2020) and three categories of referral settings (drug treatment centres, prisons, and all other settings).

## Needs assessment survey

In 2022 to 2024, NHS England organised and led a project called the “Needs Assessment”, which undertook prospective retesting of people at high risk of HCV in England, to estimate the HCV incidence amongst people who inject drugs in and out of drug treatment services in England. In 2022, 11,023 people were tested for HCV antibodies and RNA in more than 200 locations in 22 operational delivery network (ODN) regions, over a period of 6 weeks, between October and November 2022. Testing in each ODN was undertaken in drug treatment services and needle exchange services in an ad hoc manner. Follow up of all people with a valid HCV test in the first year begun in September 2023 and is currently underway as of April 2024. Testing implementation and results reporting was done in a close collaboration between NHS Trusts, the Hep C Trust, and local Drug treatment Providers. Informed consent was obtained for every patient tested.

## Summary of cost-effectiveness methods

We adopted a combined service provider perspective to evaluate the cost-effectiveness of expanded HCV testing and treatment for PWID, including the costs of HCV disease care for those with cirrhosis or more progressed disease. All costs are in 2022 British pounds. Costs from previous years were inflated to 2022 costs using the Health and Community Hospital Service pay and prices index. Health care costs and utility weights relating to HCV disease stages, injecting drug use and homelessness were taken from previous economic analyses and studies of quality of life^7-11^ - Supplementary Table 19. Utility weights were combined across different domains by taking the product with a sensitivity analysis using the minimum value ^12^.

We sought to estimate the financial and economic costs for the various strategies used to scale up testing in each ODN region. The cost estimation was done in three stages given in detail in the next section. We firstly mapped out current testing settings across each ODN through speaking to ODN leads and administrative staff and attending ODN meetings. Testing settings included drug treatment centres, prisons (including both routine testing and high-intensity test and treat sessions) and outreach services. We then used a questionnaire to gain information on the activities and resources involved, which was distributed to people involved in HCV testing in different testing settings in each region. The questionnaire was used to understand the roles of individuals involved in providing testing, the types of diagnostic tests and the unit costs of these, the time taken for testing, and the equipment involved in the testing appointment. Where HCV treatment was also provided, questions asked about how treatment was provided, who was involved, and details of the treatment regimen provided. Where possible, we obtained responses from more than one person for each type of testing setting, in order to identify differences in testing approaches between providers and across ODN regions. The responses to the questionnaire directly informed the costing analysis, with the resource use (i.e. frequency and length of appointments or testing services) being multiplied by staff salaries, using suitable NHS salary bands to get an average cost for different healthcare staff. Higher and lower bound estimates were developed for different costs based on variation in a participant’s response or across participants. If this was not possible, +/- 20% bounds were applied.

We also undertook a literature review (search done 20^th^ April 2022 on EMBASE, MEDLINE and Econlit databases) to identify additional data on resource use or costs associated with HCV testing interventions for PWID in the UK. This review was performed to identify cost data to fill gaps and supplement the resource use and cost data captured through the questionnaire. Specifically, it was used to obtain costs for undertaking fibroscan and some of the blood tests done in the workup when someone is RNA+ve.

When we were modelling no scale-up in testing from 2016, we assumed that pre-2015 testing rates and linkage to treatment continued, with the costs for this testing and treatment being estimated using cost estimates from post 2015. From 2024, we also estimated the costs of further improvements in the testing and treatment pathway using the same per person testing and treatment costs as estimated for 2016 to 2022. The cost of a course of HCV treatment is confidential so we assumed a treatment cost of £10,000 per person and varied this in the sensitivity analysis (£3,000). For each HCV case-finding scenario considered in the cost-effectiveness analysis, the transmission model was combined with relevant cost and utility data to assess the costs and QALYs associated with that scenario.

**Cost-effectiveness analysis**

The model estimated the cost-effectiveness of the existing scale-up in testing and treatment in each region by comparing the baseline model up to 2065 with a counterfactual scenario in which there was no scale-up in testing and treatment in 2016. All costs and utilities were discounted 3.5% annually. For each region, incremental cost-effectiveness ratios (ICERs) were estimated for 1,000 of the 5,000 baseline model fits as the incremental costs divided by the incremental QALYs saved over 2016-2065. The mean ICER was compared to a willingness-to-pay threshold of GBP20,000 per QALY saved as recommended by NICE ^13^. We also estimated the cost-effectiveness of any ‘improved’ strategy that managed to reach the elimination target, compared to a counterfactual of the baseline strategy over 2016-2065.

We performed sensitivity analyses to test the effect of assumptions on the ICER. These included: changing time horizon to 25 years (Baseline 50 years); changing annual discount rate to 0/5% (Baseline: 3.5%); assuming a lower (£3,000) HCV treatment cost (Baseline £10,000); assuming a higher SVR (95% vs baseline of ~85%); and taking the minimum utility value when combining utilities across domains^12^, instead of the product.

## Costing analysis of HCV testing strategies

This chapter seeks to understand how HCV testing is provided in different settings, and the resource use and costs associated with this. This includes the type of staff involved in testing, which types of tests are used and the costs of those, and costs of other associated tests that may be performed. This costing analysis considered the various HCV testing services that exist across four ODNs: Northeast and Cumbria, Nottingham, Bristol and Severn, and Greater Manchester. Understanding how testing is performed, and how patients are linked to, and monitored, during their treatment is important for estimating the costs associated with each type of testing service, since the approaches and pathways differ.

### Methods

We firstly gained an understanding of the current testing settings across each ODN by speaking to ODN leads and/or ODN administration staff, attending monthly ODN meetings where individuals meet to discuss HCV testing and treatment levels, and accessing routine UK Health Security Agency (UKHSA) reports, which also report data on testing numbers.

We then developed a questionnaire to gain more information on the testing and treatment pathway in different settings. Whilst those undertaking the questionnaire provided responses relating to their tasks, there were some aspects in which they were not able to answer, either due to it not being a primary part of their role, or due to commercial sensitivities around purchase prices. Because of these data gaps following undertaking the questionnaire, a subsequent literature review was also performed to supplement the questionnaire. The aim was to identify other relevant costing analyses of HCV testing in the UK.

Lastly, the Hepatitis C Trust played a large part in both testing and treatment support in England since the introduction of DAAs. Costing data was obtained directly from the Hepatitis C Trust (mostly from the Bristol and Severn ODN region, through co-authors Leila Reid and Sean Cox) and included in the cost analysis.

### Questionnaire Methods

A questionnaire was developed to understand how HCV testing and treatment is provided in different testing settings, and to understand the costs and resource use associated with testing and treatment. All individuals contacted worked in at least one of the ODN’s included in the study (Northeast and Cumbria, Bristol and Severn, Nottinghamshire and Greater Manchester). The methods used for the invitation of participants, administration of the questionnaire, and how the data were analysed, are provided in the sections below.

### Identifying and inviting individuals to participate

Firstly, ODN clinical leads and administrators were contacted in order to identify people in their ODN who were involved in HCV testing and who may be willing to participate in the research. Once names and contact details were shared, an email was sent asking whether individuals may be willing to participate in the questionnaire. For those providing a positive response, an information sheet about the study was provided, including an explanation of why the research was being done, and how participants could withdraw at any point if they wished. Those who agreed to participate were required to sign an informed consent sheet prior to the questionnaire being administered.

Across all ODNs, a variety of settings were considered, including drug treatment centres, prisons (including both routine testing and high-intensity test and treat sessions), and outreach service.

However, we were not able to consider every setting in which HCV testing for PWID could occur. We did not consider general practice settings, as these are relatively well understood pathways, and costs are already available ^14^. There are also other settings in which PWID could receive an HCV test, such as sexual health services, maternity of antenatal care services, hospital liver services, emergency departments, or occupational health services. However, these services are not predominantly aimed towards testing PWID, and therefore were not the focus of the costing analysis. This was a pragmatic decision to reduce the amount of interventions needing costing.

### Questionnaire design

The questionnaire was used to understand the roles of those individuals involved in organising, accompanying or providing testing, the types of diagnostic tests and the unit costs of these, the time taken for testing, and the equipment involved in the testing appointment. Where HCV treatment is also provided by the service, there were also questions about how treatment is provided, who is involved in providing and monitoring treatment, as well as details of the treatment regimen provided. The questionnaire is available at the end of the costing section (page 34). The questions were used as a guide, and it should be noted that some questions were not appropriate for certain settings. Moreover, in other instances, it was possible that the questions were too simplistic and therefore additional details were recorded during the discussions.

### Ethical approval

The questionnaire received ethical approval from the London School of Hygiene and Tropical Medicine Research Ethics Committee (Reference: 22831). This included the questionnaire itself, as well as the informed consent and information sheets.

### How and when the questionnaire was administered

The questionnaire was administered during online video calls. This was deemed the most convenient and reliable approach during the COVID-19 pandemic. Responses from participants were recorded as written notes by the researchers during the call. Those involved in the questionnaire participated between May 2021 and October 2021. Following the questionnaire, some individuals provided additional responses or costs, if additional data on costs or resource use were not available immediately during the call, or if participants had to check whether these could be shared.

### Costing Analysis

The questionnaire was administered to individuals in each of the testing settings identified. This included drug treatment services, prisons and community based or peer outreach services (from the Hepatitis C Trust). Where possible, we tried to retrieve responses from more than one person for each testing setting, in order to identify differences in testing approaches and pathways from different providers or across different ODN regions.

The responses to the questionnaire directly informed the costing analysis, with the resource use (i.e. frequency and length of appointments or testing services) being multiplied with staff costs. The salaries for different staff were derived predominantly from Personal Social Services Research Unit costings for the UK (<https://www.pssru.ac.uk/unitcostsreport/>), in order to get an average cost of different healthcare staff. Where healthcare staff were working for private companies, we tried to identify the equivalent qualification or banding for staff in the NHS (i.e. a healthcare assistant or qualified nurse).

We report the results specific to each setting without outlining the ODN from which the participants were involved in testing, to ensure confidentiality. Whilst there are differences between ODNs in terms of testing, there are also differences in the testing approaches across different providers (e.g. for prisons and drug and alcohol testing settings), some of which operate in multiple ODNs.

For cost parameter distributions, we used higher and lower values from either participant’s responses (i.e. when asking them to provide a range of how long an appointment may take), or by taking different values from different participants and using the higher and lower values for the upper and lower limits of the distribution. Where this was not possible, i.e. where there was only one participant, then the distributions were estimated using 20% upper and lower bounds of the point estimate. However, due to the very small samples sizes of questionnaire respondents, it is important to recognise that the resource use associated with each setting may not be reflective of all settings, or reflective of other ODNs.

## **Literature review for costing analysis**

A literature review was performed to identify any additional resource use or costs associated with hepatitis C testing interventions for PWID in the UK. This review was performed to identify cost data that may help to fill gaps in the resource use which were not captured through the questionnaire (e.g. the fixed costs associated with implementing testing), or to supplement the resource use or cost data derived from the questionnaire responses.

### *Search strategy*

The following databases were searched: EMBASE, MEDLINE and Econlit, and the full search terms are provided at the end of the costing section. Searches were performed on the 20^th^ April 2022, with no date restrictions. The search included both full papers and abstracts. There were no language restrictions applied. The literature review and data extraction was performed by one reviewer (Jack Williams).

### *Literature review results*

A total of 272 hits were obtained from the review, reducing to 222 following de-duplication. Of these, the hits were from Medline (63) Embase (196) and EconLit (13). A total of 30 papers were identified for full text review after screening the title and abstracts. Of these, 12 were excluded upon full text review, either due to not having cost data (3) or the cost data not being relevant (2), only an abstract being available with insufficient cost data to use for the analysis (3), or an abstract with a subsequent full paper available (4).

The 18 papers with relevant cost data are listed below. These papers did not necessarily contribute to the analysis, and instead were available to fill any gaps within the costing analysis. Furthermore, where multiple papers reported costs for a particular testing pathway, more recent papers (within the previous 10 years) were prioritised over those more than 10 years old, since the costs are likely to be more relevant. All costs from the literature review were inflated to 2022 costs.

1. Batra, N. (2001). "Hepatitis C screening and treatment versus liver transplantation: A financial option appraisal and commissioning model for purchasers." Disease Management and Health Outcomes 9(7): 371-384.
2. Buchanan, R., et al. (2020). "The testing of people with any risk factor for hepatitis C in community pharmacies is cost-effective." Journal of Viral Hepatitis 27(1): 36-44.
3. Castelnuovo, E., et al. (2006). "The cost-effectiveness of testing for hepatitis C in former injecting drug users." Health Technology Assessment (Winchester, England) 10(32): iii-iv, ix-xii, 1-93.
4. Connolly, S. P., et al. (2021). "HepCare Ireland-a service innovation project." Irish Journal of Medical Science 190(2): 587-595.
5. Darke, J., et al. (2016). "Hepatitis C in a prison in the North East of England: what is the economic impact of the universal offer of testing and emergent medications?" Journal of Public Health 38(4): e554-e562.
6. Donnan, P. T., et al. (2009). "Development of a decision support tool for primary care management of patients with abnormal liver function tests without clinically apparent liver disease: A record-linkage population cohort study and decision analysis (ALFIE)." Health Technology Assessment 13(25): ix-156.
7. Manca, F., et al. (2020). "Eradicating hepatitis C: Are novel screening strategies for people who inject drugs cost-effective?" International Journal of Drug Policy 82: 102811.
8. Martin, N. K., et al. (2013). "Cost-effectiveness of HCV case-finding for people who inject drugs via dried blood spot testing in specialist addiction services and prisons." BMJ Open 3(8): 13.
9. Martin, N. K., et al. (2016). "Is increased hepatitis C virus case-finding combined with current or 8-week to 12-week direct-acting antiviral therapy cost-effective in UK prisons? A prevention benefit analysis." Hepatology 63(6): 1796-1808.
10. Mohamed, Z., et al. (2020). "Cost-effectiveness of strategies to improve HCV screening, linkage-to-care and treatment in remand prison settings in England." Liver International 40(12): 2950-2960.
11. O'Sullivan, M., et al. (2020). "ITTREAT (Integrated Community Test - Stage - TREAT) Hepatitis C service for people who use drugs: Real-world outcomes." Liver International 40(5): 1021-1031.
12. Stein, K., et al. (2003). "Screening for hepatitis C in genito-urinary medicine clinics: a cost utility analysis." Journal of Hepatology 39(5): 814-825.
13. Stein, K., et al. (2004). "Screening for Hepatitis C in injecting drug users: a cost utility analysis." Journal of Public Health 26(1): 61-71.
14. Sutton, A. J., et al. (2006). "Estimating the cost-effectiveness of detecting cases of chronic hepatitis C infection on reception into prison." BMC Public Health 6: 170.
15. Sutton, A. J., et al. (2008). "The cost-effectiveness of screening and treatment for hepatitis C in prisons in England and Wales: a cost-utility analysis." Journal of Viral Hepatitis 15(11): 797-808.
16. Ward, Z., et al. (2019). "The cost-effectiveness of an HCV outreach intervention for at-risk populations in London, UK." Journal of Antimicrobial Chemotherapy 74(Suppl 5): v5-v16.
17. Ward, Z., et al. (2021). "Cost-effectiveness of mass screening for Hepatitis C virus among all inmates in an Irish prison." International Journal of Drug Policy 96: 103394.
18. Ward, Z., et al. (2020). "Cost-effectiveness of the HepCATT intervention in specialist drug clinics to improve case-finding and engagement with HCV treatment for people who inject drugs in England." Addiction 115(8): 1509-1521.

## Costing results

### Questionnaire uptake

A total of 11 participants were interviewed. They were represented across all four ODNs, and across a range of testing settings (Table 1). In addition to the 11 questionnaires administered, budgets for HITT testing in prisons was available from two ODNs, and budgets for testing and educational activities provided by The Hepatitis C Trust were available for two ODNs.

**Supplementary Table 1**: Participants, and the settings in which they are involved in hepatitis C testing

| **Settings** | **Number of participants** |
| --- | --- |
| Prison | 2 |
| HITT – Prison | 2* |
| Hep C Trust | 2* |
| Drug and Alcohol | 5 |
| Pharmacy | 1 |
| Probation | 1 |
| **Total** | **11** |

* Hepatitis C Trust staff were involved in HITT prison testing. The costing analysis was performed by using testing budgets from local ODN managers. Hepatitis C trust costs were estimated using local budgets, although questionnaires were used to understand procedures.

The following sections cover each testing setting included in the modelling study. We have not reported on probation or pharmacy testing because these had low coverage in our ODNs. Additionally, pharmacy testing was not preferred by our PPI group.

### Prison testing

The questionnaire was administered to two people involved in HCV testing in prisons, in two different ODNs. The testing approaches differed in each prison. One prison used DBS testing, which could occur on entry into the prison, or at a later health check. Each DBS test took an estimated 2 minutes to administer, by either a nurse or a healthcare assistant (HCA) (Supplementary Table 2). We assumed reflex RNA testing occurred because this is the standard of care in England. Following a positive test, the blood borne virus (BBV) lead nurse, or another nurse, would discuss the results and do a treatment workup. Treatment follow up is performed over the phone, in which there would be a call every week, of approximately 5 minutes. These calls were performed by the BBV lead nurse at the prison. Any adverse events or issues during monitoring would result in an additional visit (not included in this analysis). For the SVR, a venous sample would be taken by a nurse, taking approximately 5-10 minutes.

The second interviewee from the prison setting had oversight of multiple prisons across the ODN. There were different approaches to testing in each prison. The majority involved a venous blood sample taken for antibody testing, with an estimated 5 minutes required to administer by a nurse or an HCA (Supplementary Table 2). One prison had a cepheid gene Xpert machine, which uses a fingerprick blood sample and performs a rapid diagnostic test, but this was funded externally by a commercial deal with the NHS, and was not included in the current costing analysis. Those prisons taking a venous blood sample were hoping to switch to rapid diagnostics, using a fingerprick test, at some point in the future.

The local BBV lead was involved in pre-treatment visits, estimated to be 25 minutes, which included blood tests and scans performed prior to the initiation of treatment (see Supplementary Table 2 for cost and resource use details). There were also 2-3 treatment monitoring visits, depending on treatment duration. These were assumed to be approximately 10 minutes and performed by either the BBV lead nurse or another nurse.

**Supplementary Table 2**: Resource use associated with HCV testing and treatment follow-up in prisons (excludes high-intensity prison testing) used in our modelling

| **Resource use** | **Mean** | **Distribution** | **Staff member** | **Source** |
| --- | --- | --- | --- | --- |
| Time taken to perform testing - DBS (minutes) | 2 | Uniform (1.5 - 2.5) ^§^ | Nurse / HCA | Questionnaire |
| Time taken to perform testing - Venous (minutes) | 5 | Uniform (3.75 - 6.25) ^§^ | Nurse / HCA | Questionnaire |
| Proportion receiving DBS test | 0.5^†^ |  |  | TBC |
| Proportion receiving venous blood sample | 0.5^†^ |  |  | TBC |
| Appointment following positive test (minutes) | 22.5 | Uniform (20 - 25) ^‡^ | Nurse / BBV Lead Nurse | Questionnaire |
| Treatment follow up (minutes) | 7.5 | Uniform (5 - 10) ^‡^ | BBV Lead Nurse | Questionnaire |
| Proportion receiving weekly treatment follow up | 0.25 |  | BBV Lead Nurse | Questionnaire |
| Proportion receiving treatment follow up every 4 weeks | 0.75 |  | BBV Lead Nurse | Questionnaire |
| SVR appointment (minutes) | 7.5 | Uniform (5 - 10) ^‡^ | Nurse / BBV Lead Nurse | Questionnaire |

^†^One prison used DBS, one used venous samples.

^‡^Distribution based on participant responses (i.e. upper and lower limits)

^§^Distribution assumed to be 25% higher and lower than mean value

*Hepatitis C Trust prison program for opt Out Testing at reception*

Cost data from the Hepatitis C trust for Opt out testing in prisons for the Bristol ODN for 2022 is included in Supplementary Table 3.

**Supplementary Table 3**: Hepatitis C Trust prison program costs

| **Area** | **All prison peer work - Bristol ODN** |
| --- | --- |
| Salaries & Employment - 0.6 FTE Coordinator | £ 24,500.00 |
| Salaries & Employment - 0.3 FTE Manager | £ 6,045.00 |
| Management | £ 6,910.00 |
| Volunteer Costs | £ 319.50 |
| Staff development, support & Training | £ 876.00 |
| Admin / Office costs | £ 5,410.00 |
| Travel, Accommodation & subsistence | £ 6,675.00 |
| **Total** | **£ 50,735.50** |

Costs were apportioned across the three testing and treatment categories (testing, treatment and SVR) according to the percentage of time that staff took undertaking activities in each category as estimated by Hepatitis C Trust staff, see table 4 below.

**Supplementary Table 4**: Time allocation for Hepatitis C Trust for prison opt out testing

| **Category** | **What we’ve included** | **Total %** |
| --- | --- | --- |
| Testing | - Training of staff (officers, nurses, and drug services)   = 5% - Training Prisoners (prisoners in raising awareness of hep c) = 13% - Training and supporting prisoner peers (peer training, supervision) 10% - Outreach work (brief interventions, leaflet drops, 1-1 work, clinics, follow me process, in cell phone calls, supporting through treatment) = 20% - Testing (Not including HITTs) = 10% - Administration (updating records, uploading data, online training, staff supervision ) = 4% - Stakeholder engagement (meetings, 1-1 meetings) = 2% | 64% |
| Treatment support | - Outreach work (brief interventions, leaflet drops, 1-1 work, clinics, follow me process, in cell phone calls, supporting through treatment) = 20% - Training and supporting prisoner peers (peer training, supervision) = 5% - Admin (updating records, uploading data, online training, staff supervision) = 4% - Stakeholder engagement (meetings, 1-1 meetings) = 2% | 31% |
| SVR testing | - Administration (updating records, uploading data, online training, staff supervision ) = 2% - Stakeholder engagement (meetings, 1-1 meetings) = 1% | 3% |

Testing costs for the Hepatitis C Trust are allocated as fixed costs per year as they are not necessarily associated with individual tests, more with creating a willingness amongst inmates to be tested.

However, the Hepatitis C Trust treatment costs can be shared between those treated as the individuals treated are directly supported. In 2022 the Hepatitis C trust staff recorded how many people they supported through treatment each month, the sum of which was 150 person months (Bristol). Assuming treatment takes 3 months, this suggests that they helped 50 people. This is very close to the estimate of 51 treatments in prison in 2022 in the Bristol and Severn ODN region.

**Supplementary Table 5**: Mean cost of Opt-out Testing in Prison showing split between prison and healthcare and Hepatitis C Trust (Bristol and Severn)

| **Pathway category** | **Prison and Healthcare costs** | **Hepatitis C Trust costs** | **Overall costs** |
| --- | --- | --- | --- |
| Testing | £11.89 per person tested for antibody | £32,471 fixed cost per year | £11.81 per person tested for antibody  £32,471 fixed cost for each ODN per year |
| Treatment | £78.22 per person | £314.56 per person | £392.78 per person treated (not including drug costs) |
| SVR | £66.90 per person (includes test costs) | £40.59 per person | £107.49 per person (includes test costs) |

For the estimates in supplementary table 5, we assumed that 50% of tests are DBS and 50% are venous tests and that 25% of treatments have weekly follow ups and 75% have 4 weekly follow ups. The prison and healthcare costs come from applying staff salary costs and test costs to the resource use estimates in Supplementary Table 2.

The testing costs were applied to all PWID in prison according to the prison testing rate in the model and additional RNA tests costs included for those who are antibody positive. We also calculated the number in prison in each ODN per year from national prison data and applied the test cost to the proportion of the prison population who are not PWID and are therefore not included in the transmission model. In the table below we give the ranges used per person tested for the modelling.

**Supplementary Table 6: Unit costs in prison testing and treatment****

| **Item** | **Mean Cost per person Baseline (range)** | **Mean Cost per person Counterfactual (range)** |
| --- | --- | --- |
| Testing (Ab-ve) | £11.80 (£11.17-£12.42) | Same as Baseline |
| Testing (Ab +ve) | £26.80 (£23.25-£30.74) | Same as Baseline |
| Testing (previous svr) | £17.38 (£14.32-£20.79) | Same as Baseline |
| Treatment* | £394.55 (£369.29-£418.86) | £79.99 (£54.73-£104.30) |
| SVR | £106.39 (£94.25-£119.25) | £65.80 (£53.65-78.65) |

*Treatment includes baseline blood tests but not DAA cost

**Does not include fixed cost of Hepatitis C Trust involvement in testing £32,471 per year for Baseline scenario.

### High intensity test and treat (HITT) sessions in prison

HITT prison testing data was available from two ODNs. Testing in both involved a collaboration between the prison and their staff, the ODN, and the Hepatitis C Trust. The Hepatitis C Trust has given us a breakdown of the costs for the Bristol HITTs (for all staff including prison officers) and the dates and results of all the HITTs in the four ODNs. As we are modelling PWID rather than the entire prison population it does not make sense to calculate a cost per person tested to apply to the model, instead a fixed cost of HITTs carried out each year was applied in each ODN. However, we used estimates of the cost per person tested in the Bristol HITTs to estimate the HITT costs for other ODN (Supplementary Table 7). We sampled the cost per person tested from the Hepatitis C Trust data of between £25.88 and £46.24, across all the Bristol ODN HITT and applied this to the number of people who were tested in HITTs in each ODN. The cost per person tested varied depended on type of test used in each HITT (dried blood spot or matrix antibody).

We assumed the treatment and SVR costs after being diagnosed through testing in a prison session are the same as for opt-out testing in prison.

**Supplementary Table 7:** Average costs of HITTs testing interventions in each ODN (range in brackets). Costs do not include treatment and SVR costs.**

| ODN | 2020 | 2021 | 2022 | 2023 |
| --- | --- | --- | --- | --- |
| Bristol and Severn* |  | £40,810 | £17,809 |  |
| Greater Manchester |  |  |  | £14,807  (£11134 – £18560) |
| Nottingham | £32,994  (£26,090 - £40,049) | £38,749  (£30,640 -£47,035) |  | £73,954  (£55,610 -£92,700) |
| Northeast and Cumbria | £14,157  (£11,096 – £17285) |  | £50,779  (£39,801 - £61,998) | £102,210  (£80,111 - £124,790) |

*used actual costs so no uncertainty in Bristol and Severn HITT costs

** We assumed no HITTs in counterfactual scenario.

### Drug treatment centre testing

A total of 5 participants completed the questionnaire from drug treatment centres, three participants were from one provider (across multiple ODNs) and two were from a separate provider. Those participating varied considerably in terms of their role, and whether or not they were directly involved in testing and treatment. One individual was working in a co-ordination role, managing multiple services across a large geographical area. Others were involved in one or more specific services, and therefore were directly involved in offering testing and discussions with drug treatment service clients.

The time taken to perform testing (including doing paperwork and discussing risks with clients) ranged considerably across settings, from 5 minutes to 25 minutes depending on the needs of the individual. We assumed a weighted average of 12.5 minutes for each setting. This was assumed to be the case whether the test was a venous test or a DBS test. The testing was assumed to be performed by a nurse, an average of the salaries for these staff at band 5 or 6 was used in the analysis.

Following a positive test, a pre-treatment appointment was performed, taking an average of 30 minutes (ranging from 20-40 according to participants), assumed to be undertaken by a nurse (band 6). There was assumed to be travel involved in providing treatment to the individual, which ranged up to 30 minutes. We assumed a mean of 15 minutes travel time on average.

Across the settings, treatment follow up was generally performed by the Hepatitis C Trust, with the frequency of follow-up and the time taken for visits linked to the needs of the individual, and therefore varying considerably on a case-by-case basis. We did not estimate the treatment monitoring costs for drug treatment centres from the interviews - see Hepatitis C Trust section below for consideration of these costs.

Similar to the pre-treatment appointment, an SVR appointment was assumed to take 30 minutes on average, ranging from 20 to 40 minutes, and being performed by a nurse (band 6). Similar to the pre-treatment appointment, it was expected that there would be a mean 15 minutes of travel time for this appointment.

The resource use for testing at drug treatment centres and costs for incentives to encourage retention in the treatment pathway are given in Table 8. We assumed 80% of PWID in the testing and treatment pathway received incentives.

**Supplementary Table 8**: Cost and resource use associated with HCV testing and treatment in drug treatment centres

| **Resource use** | **Mean** | **Distribution** | **Staff member** | **Source** |
| --- | --- | --- | --- | --- |
| **Testing and pre-treatment/SVR appointments** |  |  |  |  |
| Time taken to perform testing - DBS or venous (minutes) | 12.5 | Uniform (5 - 25) ^†^ | Harm reduction worker | Questionnaire |
| Appointment following positive test (minutes) | 30 | Uniform (20 - 40) ^†^ | Nurse | Questionnaire |
| Appointment travel (minutes) | 15 | Uniform (0 - 30) ^†^ | Nurse | Questionnaire |
| SVR appointment - Nurse (minutes) | 30 | Uniform (20 -40) ^†^ | Nurse | Questionnaire |
| SVR appointment - Travel (minutes) | 15 | Uniform (0 - 30) ^†^ | Nurse | Questionnaire |
| **Incentives (for clients)** |  |  |  |  |
| –Completing Initial test | £10 | N/A | N/A | Questionnaire |
| Attend first treatment appointment | £5 | N/A | N/A | Questionnaire |
| –Starting treatment | £25 | N/A | N/A | Questionnaire |
| –Attending SVR12 appointment | £25 | N/A | N/A | Questionnaire |
| Proportion of individuals attending who receive incentive | 0.8 | (0.6 - 1)^§^ | N/A | Questionnaire |

^†^ Distribution based on participant responses (i.e. upper and lower limits)

^§^ Distribution assumed due to differences across ODNs and across services.

*Hepatitis C Trust Involvement at drug treatment centres*

Hepatitis C Trust (HCT) data for Bristol ODN community (non-prison) activities for 2022 gives a total cost of £150,573. This was apportioned out to testing, treatment and SVR activities according to an analysis of time spent undertaking activities relevant to each category by the HCT staff: 38% to testing, 56% to treatment support and 6% to testing for SVR. The time spent analysis was undertaken by HCT management staff in consultation with peer supporters, defining engagement and awareness building activities as testing activities. In 2022, the HCT carried out 820 tests in the community in Bristol And Severn ODN from which 245 persons were referred for treatment and 78 were recorded as treatment completions. These estimates were used to give the following cost per patient for testing, treatment and SVR: £69.24, £342.55 and £126.59. The testing costs per person through the HCT were added to the cost per person tested via DTC (using resource use estimates in supplementary Table 8). The HCT treatment and SVR costs were applied to both those treated through DTC but also through “other” setting as the numbers supported by HCT was greater than the number of treatments through DTC in Bristol and Severn in 2022.

There are no HCT costs associated to the counterfactual scenario costs. This is the only difference between the unit costs for baseline and counterfactual, with the HCT activities being seen as important for improving retention in the scale-up scenario.

**Supplementary Table 9**: Unit costs for drug treatment centres testing and treatment, combining the Hepatitis C Trust costs and other costs through the questionnaire (details in Supplementary Table 8)

| **Item** | **Baseline** | **Counterfactual** |
| --- | --- | --- |
| Testing (Ab-ve) | £100.10 (£91.88-£108.32) | £30.86 (£22.65-£39.08) |
| Testing (Ab+ve) | £115.10 (£106.88-£123.32) | £45.86 (£37.65-£54.08) |
| Testing (previous svr) | £104.63 (£96.42-£112.86) | £35.40 (£27.19-£43.62) |
| Treatment | £420.16 (£400.45-£440.84) | £195.70 (£187.17-£203.61) |
| SVR | £183.54 (£164.33-£203.83) | £24.76 (£16.53-£32.98) |

### Other Setting

The same assumptions were made as for drug treatment centres in terms of time for tests and appointments but no staff travel time is included and no incentives. Baseline blood tests and fibroscan included in treatment cost. Counterfactual does not include HCT costs.

**Supplementary Table 10:** Unit costs for testing and treatment in other settings

| **Item** | **Baseline** | **Counterfactual** |
| --- | --- | --- |
| Testing (Ab-ve) | £22.74 (£14.59-£31.08) | Same as Baseline |
| Testing (Ab+ve) | £37.74 (£29.59-£46.08) | Same as Baseline |
| Testing (previous SVR) | £27.28 (£19.13-£35.62) | Same as Baseline |
| Treatment | £538.25 (£529.72-£546.17) | £195.70 (£187.17-£203.62) |
| SVR | £151.34 (£143.12-£159.57) | £24.76 (£16.53-£32.98) |

### Test costs

We assumed that the costs for different diagnostic tests used in the scenarios above do not vary by ODN, or the service or provider which offers the testing. Therefore, only the type of diagnostic test differed in terms of the costs (e.g. a DBS antibody test would have a different cost from an antibody test on a venous blood sample, but antibody tests on DBS samples did not differ between services or across ODNs).

**Supplementary Table 11**: HCV diagnostic test costs

| **Test cost** | **Mean** | **Distribution** | **Source** |
| --- | --- | --- | --- |
| DBS card cost | £6.37 | Uniform (5.73 - 7.01)^†^ | Questionnaire participants (n = 2) |
| Antibody test cost on DBS sample | £4.09 | Uniform (3.68 - 4.49)^†^ | Questionnaire participants (n = 2) |
| Total DBS test cost | £10.46 |  |  |
| Venous antibody sample cost | £8.02 | Uniform (£7.25 - £8.52) ^‡^ | Literature review: ^7,15,16^ |
| PCR test | £61.42 | Uniform (£56 - £71.90)^‡^ | Literature review: ^7,15-17^ |
| Matrix Antibody test cost | £4.50 |  | Questionnaire participant (n=1) |

^†^ Uniform distribution +/- 10%

^‡^ Uniform distribution represents minimum and maximum values from literature review

^‡^ Rounded to nearest £0.50 due to individual value

Other tests performed

We discussed the types of tests required for HCV treatment to be dispensed with two ODN leads. In one ODN, there were no tests required, other than a recent HCV RNA test, to receive treatment. However, other tests were recommended if feasible. This includes:

- Liver function test
- Full blood count
- Aspartate transferase (AST) test
- Genotype test (if possible)

Blood test costs were included in those undergoing treatment, with fibroscans undertaken only in ‘other’ settings (Supplementary Table 12). There were numerous other tests that could be performed, but these were dependent on the person identified for testing, and what their healthcare needs may be, depending on their situation. These include the following:

- Hepatitis B test
- HIV test
- Urea and electrolytes (U&E) tests
- Ferritin test
- Autoimmune hepatitis test

The costs associated with these tests were not included because the benefits associated with diagnosis and subsequent were not included in the model (e.g. for HIV or hepatitis B diagnosis), and because we did not differentiate the need for these tests between settings.

**Supplementary Table 12**: Costs of additional tests and scans that may be performed for those testing positive for HCV

| **Resource use** | **Mean** | **Distribution** | **Source** |
| --- | --- | --- | --- |
| Liver function test | £4.76 | Uniform (£3.36 - £6.16)^†^ | ^7,17^ |
| Full blood count | £7.04 | Uniform (£3.36 - £10.73)^†^ | ^7,17^ |
| Genotype test (if possible) | £64.20 | Uniform (£51.36 - £77.04)^‡^ | ^7^ |
| Fibroscan | £154.00 | Uniform (£140 - £168)^†^ | ^7,17,18^ |
| Ultrasound | £84.56 | Uniform (£57.12 - £112)^†^ | ^7,17^ |

^†^Distribution from higher and lower values identified in the literature review.

^‡^Assumed uniform distribution with +/- 20% around the mean cost.

### Healthcare staff costs

Costs for healthcare staff were derived from the UK Personal Social Services Research Unit (PSSRU) 2021 report. Opt out testing in prison for diagnosis and SVR was assumed to be undertaken by a nurse (band 4-6), whereas appointments for those diagnosed positive were by band 5-6 and during treatment by a band 7 nurse. Testing and treatment in DTC and “other” settings was assumed to be undertaken by a nurse band 5-6. Average salary costs were calculated if multiple band types may undertake the tests/appointments.

**Supplementary Table 13**: Salaries of different staff used in the costing analysis

| **Staff / Band** | **Mean salary**^†^ | **Mean cost per hour** |  |
| --- | --- | --- | --- |
| **Community based staff** |  |  |  |
| Health Care Assistant | £19,471 | £23^‡^ | PSSRU 2021 |
| Nurse - band 4 | £28,697 | £33 | PSSRU 2021 |
| Nurse - band 5 | £36,534 | £44 | PSSRU 2021 |
| Nurse - band 6 | £46,007 | £55 | PSSRU 2021 |
| Nurse - band 7 | £55,767 | £66 | PSSRU 2021 |

^†^Mean salary includes oncosts, unless otherwise stated.

^‡^Mean hourly rate estimated from using nurse salary values from PSSRU 2021.

### Copy of Questionnaire

**NIHR - Costing analysis for HCV testing amongst PWID**

Researcher completing questionnaire to state to those involved:

The questions below are trying to understand what and how much HCV testing is performed for people who injected drugs (PWIDs) within each ODN, who is performing the testing, how much testing costs (in terms of test cost and testing administration), and finally what happens to people after testing positive (where and how they are referred/receive treatment).

The costs will then be used within a disease model, which will seek to estimate how best to improve HCV case-finding and prevention efforts in England in order to meet NHS-England HCV elimination targets.

**Date of questionnaire:**

**Role of individual involved in testing (e.g. involved in service provider or commissioner)**:

**Testing setting(s) / service provider**:

**ODN**:

**Description of testing setting/service:**

1. In this specific setting, is testing provided at full scale, or only being piloted?

- Testing performed at full scale
- Testing is provided but could be increased or scaled up
- Testing is still in pilot stage

1. Is there a weekly / monthly / annual budget allocated for testing? If so, how much is this and what does it include?

(*if there is no allocated budget specifically for this setting, then this question can be skipped)*

1. Who provides testing, and what is their role? Include all staff that are involved in the process, such as keyworkers, and include tasks such as undertaking testing, explaining testing processes, and subsequent counselling. (*tick all that apply, and estimate % if more than one person is involved in testing*)

- Clinician (Grade / Band = ________________)
- Nurse (Grade / Band = ________________)
- Healthcare assistant (Grade / Band = ________________)
- Peer worker
- Other healthcare worker (other)

Job title 1: ________________________

Job title 2: ________________________

Job title 3: ________________________

1. For each individual involved in testing, on average how many minutes does each member of staff spend for each test performed? (*as above, please include testing, explaining aspects of testing, and offering counselling*) Give details and time for each aspect of task.
2. What proportion of their time does each worker spend providing testing? If it is possible, please provide a percentage of time, or full time equivalent (FTE), which is spent providing HCV testing
3. How are samples for testing derived?

- Venous (wet) blood test
- Dried blood spot (DBS)
- Capillary bleed test
- Oral swab
- Cepheid / GeneXpert
- Other (state below):

1. Which type of testing strategy/sequence is provided at the lab or on site?

- Antibody / RNA (reflex test)
- Antibody / Antigen (reflex test)
- Antibody / RNA (return visit for RNA test)
- Antibody / Antigen (return visit for antigen test)
- Other testing strategy (state below)

1. Are any other tests provided as part of the original testing visit (e.g. fibroscan)? (*do not include tests performed as part of pre-treatment visits, as these are captured below)*
2. If possible, can you estimate or provide unit costs for the serology/virology tests? And are these test costs confidential?
3. Are there any overhead costs associated with testing in this setting, and if so, can you estimate or provide these costs? (this could be a contribution for space, or rent, use of a vehicle for outreach activities, partial use of equipment, e.g. payment/contribution towards Cepheid gene xpert machine or fibroscan machine, training to undertake testing or counselling on HCV, or any other fixed costs that occurs due to testing)
4. Do patients have a peer or a healthcare worker (or other professional, e.g. prison staff) that accompanies them to appointments? If so – who / what role?

- Yes (describe who accompanies, and who these individuals are employed by)
- Some patients are accompanied (describe below and estimate % that have this)
- No

1. Are patients incentivised to be tested in any way? If so, in what way, and how much does this cost per patient tested?
2. Do patients receive treatment on site (if eligible)? Or are they referred elsewhere for treatment?

(*please estimate the percentage of patients that receive treatment in each separate setting*)

- Treatment provided on site (i.e. same setting) (%: ______)
- Treatment delivered to patient (%: ______)
- Hospital (%: ______)
- GP (%: ______)
- Community pharmacy (%: ______)
- Drug treatment centre (%: ______)
- Other (state below)

_______________________________________ (%: ______)

1. For those referred elsewhere, is this due to clinical factors (e.g. cirrhosis) or another reason?

**# IF TREATMENT IS PROVIDED ON SITE, THE FOLLOWING QUESTIONS AROUND THE NUMBER OF VISITS AND WHO PROVIDES TREATMENT WILL BE ASKED #**

**Pre-treatment visits**

Once a patient is tested positive, how many pre-treatment visits do they have, and what is performed at each visit?

| **Components** | **Visit 1** | **Visit 2** | **Visit 3** |
| --- | --- | --- | --- |
| Blood tests |  |  |  |
| Fibroscan |  |  |  |
| Ultrasound |  |  |  |
| Genotype test |  |  |  |
| - |  |  |  |
| - |  |  |  |
| - |  |  |  |

Comments:

Which members of staff are involved in providing pre-treatment visits, and how much time (in minutes) is spent by each member of staff, on average, and are visits face to face or virtual (e.g. over the phone)?

| **Staff member** | **Visit 1** | **Visit 2** | **Visit 3** |
| --- | --- | --- | --- |
| **Face to face (F2F) or virtual / call?** |  |  |  |
| Specialist nurse |  |  |  |
| Hepatologist |  |  |  |
| Healthcare worker (other) |  |  |  |
| Peer worker |  |  |  |

Comments:

How many treatment visits occur, and when do these occur (e.g. weeks 0, 2, 4, 6, 8, 10, 12)?

Which staff members are involved in each treatment visit? And what tests are performed in each visit?

Please indicate approximately how much time each visit takes, in minutes.

| **Staff member** | **Visit 1** | **Visit 2** | **Visit 3** | **Visit 4** | **Visit 5** | **Visit 6** |
| --- | --- | --- | --- | --- | --- | --- |
| **Week of visit**  **(F2F / call)** |  |  |  |  |  |  |
| **Total time per visit** |  |  |  |  |  |  |
| Specialist nurse |  |  |  |  |  |  |
| Hepatologist |  |  |  |  |  |  |
| Healthcare worker (other) |  |  |  |  |  |  |

Comments:

**Post-treatment follow-up visits**

Which staff members are involved in post-treatment follow-up appointments? When are these appointments performed? And what tests are performed in each visit?

Please indicate approximately how much time each visit takes, in minutes.

| **Staff member** | **Visit 1** | **Visit 2** | **Visit 3** | **Visit 4** |
| --- | --- | --- | --- | --- |
| **Week of visit -**  **from rx ending**  **(F2F / call)** |  |  |  |  |
| **Total time per visit** |  |  |  |  |
| Specialist nurse |  |  |  |  |
| Hepatologist |  |  |  |  |
| Healthcare worker (other) |  |  |  |  |
| Peer worker |  |  |  |  |
| Prison worker |  |  |  |  |
| Other key worker  (please state who in comments) |  |  |  |  |

**Literature review search terms and databases**

Searches run on 20^th^ April 2022

**EMBASE**

1. exp hepatitis C/

2. exp Hepatitis C virus/

3. exp hepatitis C antibody/

4. exp hepatitis C antigen/

5. (hepatitis c or hcv or hepacivirus*).ti,ab.

6. or/1-5

7. exp economic evaluation/

8. exp economic aspect/

9. hidden markov model/

10. exp cost/

11. (cost* or economic*).ti,ab.

12. ((disease or decision or markov) adj3 (model* or evaluation)).ti,ab.

13. or/7-12

14. exp screening/

15. (screen* or test* or diagnos* or case finding).ti,ab.

16. or/14-15

17. Substance Abuse, Intravenous/

18. (drug* or substance*).ti,ab,kf.

19. (abuse* or depend* or use* or misus* or addict* or inject* or intravenous).ti,ab,kf.

20. (pwid or pwud or idu or ivdu).ti,ab.

21. 18 and 19

22. 17 or 20 or 21

23. Opiate substitution treatment/

24. Methadone/

25. Buprenorphine/

26. (OST or OAT or MMT).ti,ab.

27. ((opioid* or opiate* or methadone or buprenorphine) adj2 (substitut* or maint* or agonist or treat* or therapy)).ti,ab.

28. or/23-27

29. (homeless* or prison* or jail or probation or justice).ti,ab.

30. 22 or 28 or 29

31. (United Kingdom or UK or Britain or British or GB or Engl* or Wales or Welsh or Ireland or irish or scotland or scottish or NHS or national health service or NICE or national institute for health).ti,ab.

32. 6 and 13 and 16 and 30 and 31

33. limit 32 to yr="2000 -Current"

**MEDLINE**

1. exp Hepatitis C/

2. Hepacivirus/

3. (hepatitis c or hcv or hepacivirus*).ti,ab.

4. exp Hepatitis C Antigens/ or exp Hepatitis C Antibodies/

5. or/1-4

6. exp models, economic/

7. markov chains/

8. Mass Screening/ec

9. exp cost/

10. (cost* or economic*).ti,ab.

11. ((disease or decision or markov) adj3 (model* or evaluation)).ti,ab.

12. or/6-11

13. Mass Screening/

14. (screen* or test* or diagnos* or case finding).ti,ab.

15. or/13-14

16. Substance Abuse, Intravenous/

17. (drug* or substance*).ti,ab,kf.

18. (abuse* or depend* or use* or misus* or addict* or inject* or intravenous).ti,ab,kf.

19. (pwid or pwud or idu or ivdu).ti,ab.

20. 17 and 18

21. 16 or 19 or 20

22. Opiate substitution treatment/

23. Methadone/

24. Buprenorphine/

25. (OST or OAT or MMT).ti,ab.

26. ((opioid* or opiate* or methadone or buprenorphine) adj2 (substitut* or maint* or agonist or treat* or therapy)).ti,ab.

27. or/22-26

28. (homeless* or prison* or jail or probation or justice).ti,ab.

29. 21 or 27 or 28

30. (United Kingdom or UK or Britain or British or GB or Engl* or Wales or Welsh or Ireland or irish or scotland or scottish or NHS or national health service or NICE or national institute for health).ti,ab.

31. 5 and 12 and 15 and 29 and 30

**Econlit**

1. (hepatitis c or hcv or hepacivirus).mp.

2. (cost* or economic*).mp.

3. (screen* or test* or case finding).mp.

4. 1 and 2 and 3

## Patient and public involvement

**Aim**

Workshops with PWID were run to discuss the strengths, limitations, gaps and inefficiencies of current local case-finding strategies and to gather feedback on new possible strategies.

**Methods**

In total, we involved 22 people with lived experience of hepatitis C testing and / or treatment across three ODNs: Bristol and Severn; Nottingham and Northeast and Cumbria (we were unable to do a workshop in Manchester due to ODN restructuring). This included men and women and people who had experienced both homelessness and incarceration. Due to COVID restrictions, we spoke to 5 people by phone (3 in Bristol and Severn and 2 in Nottingham). When restrictions eased, we conducted two workshops in person in Lincoln (Nottingham ODN) and Middlesbrough (Northeast and Cumbria ODN) with 8 and 9 participants respectively. One telephone conversation has been held with a person with lived experience in Cumbria to explore more rural experiences. Topics covered in the workshops included:

- Knowledge of and attitude towards available strategies
- Barriers to access / engagement
- Facilitators to access / engagement
- Coverage of strategies
- Changes to services to support access

**Results of patient and public involvement input**

Feedback from the workshops across the 3 ODNs was remarkably consistent. Issues raised included:

- Testing settings, e.g community outreach and probation services were viewed positively but testing in pharmacies was viewed negatively. This was because discussions with staff might take place in reception areas which are not private and because staff in pharmacies may have negative views about PWID.
- Testing and treatment delivery and logistics, e.g blood spot test vs blood sample, self testing, result waiting times were viewed as significant
- Targeting testing, e.g reaching people who are not working and do not access services has a significant impact
- Initiatives to support increased testing and treatment and prevent reinfection were emphasised, e.g incentives

Based on the feedback we developed potential implications for the modelling work and documented the outcome and impact of the PPI findings on the modelling work. The results of the PPI meetings have also been presented to the ODN leads.

We held a final PPI workshop in Bristol to assess the acceptability and coverage of our model, i.e. have we missed certain subgroups? Given the similarity of feedback from workshops across the ODNs we decided that one workshop in Bristol would be sufficient. Five men, all with experience of hepatitis C testing and treatment attended this workshop. At the workshop we presented the findings from the modelling and the predicted reduction in cases of hepatitis C if current strategies are continued. Attendees in the workshop confirmed that in their experience it is not difficult to access hepatitis C testing in Bristol and that, if found positive, testing commences within a few weeks. They also confirmed that testing and treatment of people who are still injecting takes place, which was seen as very positive. The feedback from workshop participants suggests that the declines in hepatitis C predicted by our model are based on realistic assumptions.

**Discussion of patient and public involvement input**

Many different interventions have been piloted for improving testing and linkage to HCV treatment among PWID in different regions of England and elsewhere. However, it is uncertain whether many of these interventions were developed in discussion with the community of PWID to better understand what is needed to improve current strategies. The patient and public involvement component of this project has been important in helping gain an understanding of the weaknesses in current or planned approaches and how specific factors can affect their acceptance. This led us to not model an expansion of testing in pharmacies or sexual health clinics because these were not viewed well by the community. Conversely, there was enthusiasm for outreach testing and additional testing in prison, with both having been expanded in England and included in the model. However, we did not specifically include outreach testing among homeless because we had little data to determine the amount of testing occurring in this group. There was also enthusiasm for post-prison release testing with some ODNs having started this strategy. We did not include this as one of the potential scale-up strategies in the model because it was considered to overlap considerably with the existing high level of testing in prisons. There was also enthusiasm for testing in recovery settings and in rural areas. Rural settings were not explicitly included in our model, although through modelling outreach testing linked to drug treatment centres we should have included that. Unfortunately, we did not have any specific data available on recovery services. It is likely to overlap with OAT and the drug treatment centre population, but we don’t have information on whether they’re in recovery or not. Lastly, incentives were included in our economic evaluations, which was suggested by the community to be an initiative to support testing and treatment.

**Reflections and critical perspective**

Overall, the workshops involving PWID with lived experience of hepatitis C testing and / or treatment were a valuable addition to the study because it highlighted strategies that should or should not be expanded. However, it was difficult to undertake these workshops during the COVID-19 pandemic because in person meetings were not possible. Because of these restrictions, we attempted to do some of the workshops individually on the phone, which was particularly difficult in this group because some do not have phones or access other devices like tablets. Where people do have mobile phones, they may be on pay as you go contracts which means that participating via mobile phone may create additional cost for people who are already struggling to make ends meet. It also prevented us having workshops involving multiple people because the logistics of organising enough people at the same time for a conference call proved difficult to achieve. We followed advice from ODN staff and decided to opt for individual discussions. Once restrictions eased, we undertook in person workshops which were much more fruitful. However, this was not possible for rural individuals, and we only managed to involve one rural individual in the patient and public involvement highlighting that more needs to be done to involve people from this group.

**Conclusion**

The public involvement for this project was conducted in difficult circumstances with a group of people who in the past have not been involved in inputting into this type of research. Nevertheless, we were able to gain important insights into the realities of implementing case findings strategies based on the lived experience of PWID. The PPI workshops allowed us to test our assumptions and ground our work more firmly in the day-to-day realities of life for PWIDs.

## Model Calibration Algorithm

Data from multiple sources were used to calibrate the model for each of the four operational delivery networks: Greater Manchester, Northeast and Cumbria, Nottingham and Bristol and Severn.

**Approximate Bayesian Computation Calibration process**

For each setting, an Approximate Bayesian Computation sequential Monte Carlo scheme (ABC SMC) routine ^19^ was used to obtain a number of parameter sets that fit the calibration data (Supplementary Tables 15a-15h). The ABC SMC is an iterative algorithm that begins by sampling parameter sets from the prior distributions (5,000; priors in Supplementary Tables 16a-16e and 17a-17e and 18) and evaluating for each parameter set the goodness of fit - according to some distance function. The tolerance of the next iteration is then set as the 85% percentile of the goodness of fits of the 5,000 sampled parameter sets and the next iteration begins.

At subsequent iterations, parameter sets from the previous iteration are sampled from, with weights dependent upon the prior likelihood of the parameter set and the perturbation kernel. These sampled parameter sets are then perturbed using a uniform perturbation kernel, which can perturbate each parameter by at most +/- 20% of the prior range, so as to still be within the prior ranges for each parameter, accepting those that gave model fits whose goodness of fit, measured by the distance function, was better than the 85^th^ percentile of accepted model fits in the previous iteration (the tolerance). Sampling of parameter sets continues, until the required number are accepted. The tolerance of the next iteration is then set as the 85^th^ percentile of the goodness of fits of the accepted parameter sets and the next iteration begins. The ABC SMC routine continued iteratively until the tolerances began to stabilise. The parameter sets obtained at the end of the routine were then used to initialize the model for that setting and to project the outcomes for the baseline scenario until end 2030 or 2065 (for cost-effectiveness analysis).

In our implementation, we took the distance function between the model outputs {y} and the data {x} to be the sum of the absolute normalised error for each data point, i.e. $\frac{\left| y-x \right|}{x}.$This model calibration used the incarceration sub-model and the full model simultaneously, with details of what was calibrated to in the description for each setting below.

The data points are grouped together so that the distance function has 5 elements:

1. HCV Ab prevalence, OAT and NSP coverage in community and OAT coverage in prison, % ever and recently incarcerated in community and RNA positivity among those that are Ab-positive in the community;
2. Percent currently and ever homeless and population size;
3. Odds ratio of current homelessness in recent versus non-recent incarcerated, odds ratios of being HCV Ab+ve in ever vs never incarcerated and ever vs never homeless, and odds ratio of being on OAT if currently homelessness versus not currently homeless;
4. Cohort model of proportion ever incarcerated by injecting duration and number of times incarcerated by injecting duration;
5. Cumulative number received HCV treatment in different settings.

Within element one, for the prevalence of RNA positivity among those Ab-positive and HCV Ab prevalence, the distance function was calculated as the absolute normalised error or zero (if the model output was within the minimum and maximum data points for that element).

A comparison of the calibrated model to different data points is shown in Supplementary figures 4 and 5, which shows that the model generally agrees with the calibration data. A comparison of the prior and posterior ranges from the ABC scheme for calibrating the model to each region are included in Supplementary Tables 16a to 17e, with a comparison of the prior and posterior distributions for those parameters that did not have narrow priors being included in Supplementary Figure 3. Supplementary Figure 2 also shows that the error measures used in the ABC scheme reduce with each calibration iteration. In summary, we found that the model calibration narrowed nearly all of the prior parameter ranges, as shown in Supplementary Figure 3. However, some prior ranges are not narrowed as much ,with this uncertainty being propagated to our model projections. This includes such things as the time to treatment, which has relatively narrow priors and some of the linkage to treatment parameters, which can take different values depending on the diagnosis rate.

## Supplementary data tables for model parameterization and calibration

**Supplementary Table 15:** Data estimates use for model calibration

**Supplementary Table 15a** Data points used for all settings with sample size or 95% CI in brackets

| **Model measure** | **Estimate** | **Source** |
| --- | --- | --- |
| Percentage OAT in prison in 2013 | 54.3% (12260) | ^20^ |
| Odds ratio of homelessness in recent vs non-recent incarceration in 2017 | 1.48 (1.06,2.07) | UAM survey data analysis for this paper |
| Odds ratio of OAT in current vs not current homeless | 0.73 (0.63,0.85) | UAM survey data analysis for this paper |
| RNA positivity amongst those testing antibody-positive in 2014 | 72.3% (40856) | From sentinel surveillance^6^ * |

* this data point is used for model calibration for all ODN as an additional estimate of the RNA prevalence amongst people who test antibody positive because it is thought that the UAM may under estimate RNA prevalence in the years before HCV treatment scaled up.

**Supplementary Table 15b** Data points used for calibrating the model for Bristol and Severn, all are UAM data analyses for this paper unless otherwise stated. Sample sizes or 95% CI are in brackets.

| **Data type** | **2004 ^21^** | **2006 ^22^** | **2009 ^22^** | **2011-2013** | **2014-2015** | **2016-2017** | **2018-2019** | **2020-2021^** | **2022 Needs Assessment** |
| --- | --- | --- | --- | --- | --- | --- | --- | --- | --- |
| Proportion in community who have been recently released (within 6 months) |  |  |  |  |  |  | 22.0% (260) |  |  |
| HCV Antibody prevalence | 64.8% (202) | 59.2% (299) | 59.8% (336) | 36.0% (397) | 57.5% (209) | 70.0% (246) | 66.0% (307) | 62.9% (35) | 52.1% (376) |
| RNA prevalence among those testing antibody positive* |  |  |  | 57.0% (135) | 60.0% (171) | 58.0% (163) | 58.0% (196) | 18.2% (22) | 20.1% (184) |
| NSP coverage (used in last year) |  |  |  | 88.0% 363) | 92.0% (286) | 94.1% (247) | 91.0% (289) |  |  |
| OAT coverage currently |  |  |  | 69.0% (383) | 60.0% (305) | 65.0% (254) | 69.0% (300) |  |  |
| Homelessness in last year (used as proxy for currently homeless) | 68.8% (202) | 58.2% (299) | 59.8% (336) | 20.0% (91) | 31.9% (299) | 34.5% (246) | 38.0% (274) |  |  |
| Homelessness ever but not currently |  |  |  | 80.1% (365) | 82.6% (299) | 814.4% (246) | 89.0% (274) |  |  |
| Ever incarcerated but not currently |  |  |  | 67.3% (383) | 67.6% (307) | 67.2% (252) | 72.0% (284) |  |  |
| Population size of current PWID@ |  |  |  |  |  | 6180+/- 50% |  |  |  |
| Odds ratio of HCV Ab+ in ever vs never incarcerated |  |  |  |  |  | 3.33 (2.59-4.29) |  |  |  |
| Odds ratio of HCV Ab+ in ever vs never homeless |  |  |  |  |  | 2.40 (1.75-3.30) |  |  |  |

@ UKHSA ODN treatment tool developed by Ross Harris^23-25^

* An extra RNA prevalence estimate from sentinel surveillance (supplementary table 15a) is used for model calibration because it is thought that the UAM may underestimate the RNA prevalence among those testing antibody positive in the years before HCV treatment scaled up

^2020-2021 UAM data was not used for model fitting.

**Supplementary Table 15c** Data points used for calibrating the model for Greater Manchester, all are UAM data analyses unless otherwise stated. Sample sizes or 95% CI are in brackets.

| **Data type** | **2011-2013** | **2014-2015** | **2016-2017** | **2018-2019** | **2020-2021^** | **2022 Needs Assessment** |
| --- | --- | --- | --- | --- | --- | --- |
| Proportion in community who have been recently released (within 6 months) |  |  |  | 21.0% (114) |  |  |
| HCV Antibody prevalence | 56.0% (203) | 58.0% (128) | 69.0% (148) | 64.0 (134) | 68.0% (25) | 52.3% (153) |
| RNA prevalence among those testing antibody positive* | 64.0% (74) | 52.0% (65) | 55.0% (93) | 48.0% (82) | 29.4% (17) | 27.0% (74) |
| NSP coverage (used in last year) | 89.0% (198) | 86.0% (117) | 90.0% (162) | 84.0% (122) |  |  |
| OAT coverage currently | 78.8% (203) | 84.8% (126) | 83.8% (169) | 88.5% (135) |  |  |
| Homelessness in last year (used as proxy for currently homeless) | 28.0% (18) | 20.0% (125) | 25.9% (160) | 30.3% (124) |  |  |
| Homelessness ever but not currently | 75.0% (197) | 79.0% (125) | 80.8% (160) | 75.1% (124) |  |  |
| Ever incarcerated but not currently | 78.7% (202) | 77.4% (125) | 77.5% (166) | 70.6% (126) |  |  |
| Population size of current PWID@ |  |  | 8900 +/-50% |  |  |  |
| Odds ratio of HCV Ab+ in ever vs never incarcerated |  |  | 2.67  (1.80- 3.95) |  |  |  |
| Odds ratio of HCV Ab+ in ever vs never homeless |  |  | 1.89  (1.27 - 2.81) |  |  |  |

@ UKHSA ODN treatment tool developed by Ross Harris^23-25^

* An extra RNA prevalence estimate from sentinel surveillance (supplementary table 15a) is used for model calibration because it is thought that the UAM may underestimate the RNA prevalence among those testing antibody positive in the years before HCV treatment scaled up

^2020-2021 UAM data was not used for model fitting.

**Supplementary Table 15d** Data points used for calibrating the model for Nottingham, all are UAM data analyses for this paper unless otherwise stated. Sample sizes or 95% CI are in brackets.

| **Data type** | **2011-2013** | **2014-2015** | **2016-2017** | **2018-2019** | **2020-2021^** | **2022 Needs Assessment** |
| --- | --- | --- | --- | --- | --- | --- |
| Proportion in community who have been recently released (within 6 months) |  |  |  | 25.5% (272) |  |  |
| HCV Antibody prevalence | 48.0% (644) | 55.2% (426) | 54.7% (276) | 47.0% (247) | 56.8% (192) | 44.7% (199) |
| RNA prevalence among those testing antibody positive* | 52.0% (283) | 58.0% (225) | 52.0% (139) | 54.0% (102) | 26.0% (104) | 17.7% (85) |
| NSP coverage (used in last year) | 91.1% (615) | 93.9% (403) | 92.8% (296) | 88.9% (280) |  |  |
| OAT coverage currently | 67.0% (633) | 65.9% (423) | 77.2% (317) | 75.1% (300) |  |  |
| Homelessness in last year (used as proxy for currently homeless) | 22.2% (234) | 24.9% (412) | 22.7% (269) | 22.8% (280) |  |  |
| Homelessness ever but not currently | 81.0% (599) | 82.0% (412) | 77.9% (269) | 76.1% (280) |  |  |
| Ever incarcerated but not currently | 78.8% (621) | 75.2% (421) | 74.0% (269) | 75.1% (282) |  |  |
| Population size of current PWID@ |  |  | 8840 +/-50% |  |  |  |
| Odds ratio of HCV Ab+ in ever vs never incarcerated |  |  | 2.29  (1.79–2.93) |  |  |  |
| Odds ratio of HCV Ab+ in ever vs never homeless |  |  | 2.26  (1.73–2.95) |  |  |  |

@ UKHSA ODN treatment tool developed by Ross Harris^23-25^

*An extra RNA prevalence estimate from sentinel surveillance (supplementary table 15a) is used for model calibration because it is thought that the UAM may underestimate the RNA prevalence among those testing antibody positive in the years before HCV treatment scaled up

^2020-2021 UAM data was not used for model fitting.

**Supplementary Table 15e** Data points used for calibrating the model for Northeast and Cumbria, all are UAM data analyses for this paper unless otherwise stated. Sample sizes or 95% CI are in brackets.

| **Data type** | **2011-2013** | **2014-2015** | **2016-2017** | **2018-2019** | **2020-2021^** | **2022 Needs Assessment** |
| --- | --- | --- | --- | --- | --- | --- |
| Proportion in community who have been recently released (within 6 months) |  |  |  | 20.2% (257) |  |  |
| HCV Antibody prevalence | 36.2% (738) | 40.8% (394) | 43.5% (387) | 58.0% (260) | 80.5% (82) | 41.7% (247) |
| RNA prevalence among those testing antibody positive* | 47.0% (227) | 45.0% (35) | 44.0% (140) | 57.0% (143) | 43.1% (65) | 44.1% (84) |
| NSP coverage (used in last year) | 90.0% (686) | 92.0% (368) | 95.1% (478) | 93.7% (280) |  |  |
| OAT coverage currently | 68.4% (720) | 72.4% (393) | 61.0% (507) | 64.6% (289) |  |  |
| Homelessness in last year (used as proxy for currently homeless) | 21.9% (216) | 17.4% (379) | 27.6% (495) | 32.0% (278) |  |  |
| Homelessness ever but not currently | 75.6% (676) | 73.9% (379) | 77.6% (495) | 74.8% (278) |  |  |
| Ever incarcerated but not currently | 73.9% (705) | 71.5% (383) | 71.2% (499) | 66.2% (282) |  |  |
| Population size of current PWID@ |  |  | 13310+/-50% |  |  |  |
| Odds ratio of HCV Ab+ in ever vs never incarcerated |  |  | 2.62 (2.09 – 3.29) |  |  |  |
| Odds ratio of HCV Ab+ in ever vs never homeless |  |  | 1.86 (1.47 – 2.35) |  |  |  |

@ UKHSA ODN treatment tool developed by Ross Harris^23-25^

* An extra RNA prevalence estimate from sentinel surveillance (supplementary table 15a) is used for model calibration because it is thought that the UAM may underestimate the RNA prevalence among those testing antibody positive in the years before HCV treatment scaled up

^2020-2021 UAM data was not used for model fitting.

**Supplementary Table 15f** Percentage ever Incarcerated by duration of injecting in each ODN region - used for calibrating the incarceration sub model (Source UAM data analyses for this paper)

| **Injecting Duration (years)** | **Bristol and Severn** | **Greater Manchester** | **Northeast and Cumbria** | **Nottingham** |
| --- | --- | --- | --- | --- |
| 0-1 | 31.4% (39) | 73.0% (15) | 77.3% (66) | 60.6% (45) |
| 0-4 | 45.1% (214) | 63.6% (74) | 50.0% (334) | 51.5% (211) |
| 5-9 | 55.0% (172) | 65.0% (57) | 67.0% (328) | 67.7% (210) |
| 10-14 | 64.5% (212) | 70.7% (92) | 76.1% (387) | 79.4% (345) |
| 15-19 | 74.4% (216) | 86.7% (107) | 82.2% (349) | 84.0% (311) |
| 20-24 | 84.2% (183) | 81.8% (131) | 80.0% (198) | 88.6% (205) |
| 25+ | 85.0% (591) | 82.0% (122) | 85.1% (131) | 84.4% (172) |

Data from multiple rounds of UAM 2011 to 2019, N in brackets

**Supplementary Table 15g** Number of times incarcerated by duration of injecting - used for calibrating the incarceration sub model (Source UAM data analyses for this paper)

| **Injecting Duration (years)** | **Bristol and Severn** | **Greater Manchester** | **Northeast and Cumbria** | **Nottingham** |
| --- | --- | --- | --- | --- |
| 0-4 | 5.77 | 7.12 | 6.56 | 5.75 |
| 5-9 | 6.20 | 6.58 | 6.37 | 6.43 |
| 10-14 | 7.83 | 6.52 | 7.77 | 7.61 |
| 15-19 | 9.85 | 8.58 | 7.96 | 9.15 |
| 20-24 | 9.76 | 7.78 | 11.38 | 9.72 |
| 25+ | 9.81 | 10.81 | 12.01 | 9.52 |

Data from multiple rounds of UAM 2011 to 2019

**Supplementary Table 15h** Treatment numbers in PWID (Source Treatment Database and Blueteq)

|  | **Bristol and Severn** | **Northeast and Cumbria** | **Greater Manchester** | **Nottingham** |
| --- | --- | --- | --- | --- |
| **Drug Treatment Centres** | |  |  |  |
| 2016 | 8 | 50 | 88 | 86 |
| 2017 | 50 | 125 | 113 | 86 |
| 2018 | 86 | 162 | 152 | 104 |
| 2019 | 129 | 207 | 246 | 148 |
| 2020 | 88 | 228 | 222 | 202 |
| 2021 | 170 | 275 | 188 | 201 |
| 2022 | 80 | 309 | 197 | 195 |
| **Prison lower bound** |  |  |  |  |
| 2016 | 1 | 9 | 32 | 14 |
| 2017 | 10 | 92 | 55 | 23 |
| 2018 | 14 | 159 | 117 | 30 |
| 2019 | 47 | 115 | 198 | 79 |
| 2020 | 50 | 191 | 115 | 74 |
| 2021 | 61 | 143 | 60 | 87 |
| 2022 | 51 | 120 | 38 | 44 |
| **Prison upper bound** |  |  |  |  |
| 2016 | 13 | 25 | 62 | 56 |
| 2017 | 21 | 110 | 96 | 84 |
| 2018 | 21 | 167 | 188 | 99 |
| 2019 | 55 | 123 | 298 | 142 |
| 2020 | 69 | 200 | 156 | 122 |
| 2021 | 80 | 155 | 101 | 135 |
| 2022 | 55 | 123 | 59 | 72 |
| **Other settings** |  |  |  |  |
| 2016 | 33 | 128 | 129 | 18 |
| 2017 | 59 | 143 | 80 | 23 |
| 2018 | 90 | 160 | 57 | 48 |
| 2019 | 88 | 112 | 79 | 51 |
| 2020 | 91 | 129 | 105 | 45 |
| 2021 | 101 | 217 | 72 | 25 |
| 2022 | 144 | 281 | 79 | 20 |

**Priors and posteriors for the model parameters**

Most priors are the same for all settings. Those that are not include the HCV seed prevalence, the proportion of initiates to injecting who have never been incarcerated and the proportion who have never been homeless. The priors for these are estimated using UAM data for each setting.

**Supplementary Table 16**: Priors and Posteriors (ranges are min to max) for baseline model parameters

**Supplementary Table 16a** Parameters for the entry and exit of PWID to the modelled population

|  | **Northeast and Cumbria Posterior range** | **Bristol and Severn Posterior range** | **Nottingham Posterior range** | **Greater Manchester Posterior ranges** | **Prior Distribution** | **Source** |
| --- | --- | --- | --- | --- | --- | --- |
| Drug related mortality rate per year | 0.03 (95%CrI 0.022 - 0.037) | 0.028 (95%CrI 0.022 - 0.035) | 0.033 (95%CrI 0.024 - 0.038) | 0.031 (95%CrI 0.025 - 0.036) | Uniform (0.0216, 0.0394) | All cause mortality when not on OAT from systematic review ^26^ |
| Proportion of new injectors who have never been incarcerated | 0.449 (95%CrI 0.368 - 0.521) | 0.663 (95%CrI 0.596 - 0.733) | 0.48 (95%CrI 0.391 - 0.565) | 0.382 (95%CrI 0.297 - 0.466) | Uniform (0.3, 1) Bristol  Uniform (0.3, 0.6) Northeast  Uniform (0.3,0.6) Nottingham  Uniform (0,0.5) Manchester | UAM data estimates a proportion of recent initiates to injecting (<1yr) have not been incarcerated before |
| Proportion of new injectors who are currently incarcerated (out of those ever incarcerated) | 0.294 (95%CrI 0.023 - 0.814) | 0.239 (95%CrI 0.014 - 0.705) | 0.366 (95%CrI 0.015 - 0.89) | 0.268 (95%CrI 0.037 - 0.751) | Uniform (0, 1) | No data so uninformed prior |
| Maximum number of times new initiates have ever been incarcerated | 8.947 (95%CrI 7.198 - 9.962) | 6.47 (95%CrI 3.889 - 9.255) | 8.553 (95%CrI 6.206 - 9.912) | 8.669 (95%CrI 6.815 - 9.929) | Uniform (1, 10) | UAM data estimates that recent initiates to injecting (<1 yr) who have been incarcerated before have a mean of 6.8 times |
| Injecting duration in years until permanent cessation | 12.193 (95%CrI 10.149 -16.874) | 11.638 (95%CrI 10.120-14.289) | 12.603 (95%CrI 10.127 -17.424) | 12.583 (95%CrI 10.264 - 14.983) | Uniform (10, 20) | Assumption based on UAM data of duration currently IDU |
| Proportion of new injectors who have never been homeless | 0.293 (95%CrI 0.239 - 0.326) | 0.232 (95%CrI 0.171 - 0.318) | 0.339 (95%CrI 0.255 - 0.41) | 0.332 (95%CrI 0.265 - 0.424) | Uniform (0.11,0.41) Bristol  Uniform (0.17,0.41) Northeast  Uniform (0.2,0.5) Nottingham  Uniform (0.24, 0.76) Manchester | UAM data estimates proportion of recent initiates (<1yr) have never been homeless |

**Supplementary Table 16b** Incarceration Parameters

|  | Northeast and Cumbria Posterior range | Bristol and Severn Posterior range | Nottingham Posterior ranges | Greater Manchester Posterior ranges | Prior Distribution | Source |
| --- | --- | --- | --- | --- | --- | --- |
| Incarceration rate per year | 0.070  (95%CrI 0.047-0.092) | 0.076  (95%CrI 0.058-0.094) | 0.079  (95%CrI 0.051-0.099) | 0.058  (95%CrI 0.035 - 0.077) | Uniform (0, 0.1) | Uninformed prior |
| Re-incarceration rate per year | 0.583  (95%CrI 0.442-0.749) | 0.830  (95%CrI 0.622-0.984) | 0.623  (95%CrI 0.435-0.828) | 0.47 (95%CrI 0.328 - 0.612) | Uniform (0, 1) | Uninformed prior |
| Prison duration in years | 0.691  (95%CrI 0.465 - 0.926) | 0.394  (95%CrI 0.270-0.584) | 0.652  (95%CrI 0.403-1.029) | 0.943  (95%CrI 0.711 - 1.061) | Uniform (0.25,1.06) | UAM data (3-12.8 mths) |

**Supplementary Table 16c** Homelessness parameters

|  | **Northeast and Cumbria Posterior range** | **Bristol and Severn Posterior range** | **Nottingham Posterior ranges** | **Greater Manchester Posterior ranges** | **Prior Distribution** | **Source** |
| --- | --- | --- | --- | --- | --- | --- |
| Duration of homelessness in years | 1.223 (95%CrI 0.883-1.91) | 1.34 (95%CrI 0.903-2.06) | 1.446 (95%CrI 0.925-2.175) | 1.483 (95%CrI 0.982-1.916) | Uniform (0.86, 2.49) | Kemp et al. found 77 of 143 PWID not homeless after 8 months^27^ and Craine et al found 33% of PWID no longer homeless after 1 year ^28^. Converted 95% CI of these estimates to rates and inverted to get duration of homelessness. |
| Rate of becoming homeless if never homeless before | 0.025 (95%CrI 0.002 - 0.061) | 0.028 (95%CrI 0.003 - 0.06) | 0.025 (95%CrI 0.002 - 0.071) | 0.019 (95%CrI 0.001 - 0.045) | Uniform  (0 0.1) | Uninformative prior – estimated through calibrating to % ever and currently homeless |
| Rate of becoming homeless if ever homeless | 0.325 (95%CrI 0.15 - 0.577) | 0.334 (95%CrI 0.159 - 0.61) | 0.245 (95%CrI 0.11 - 0.426) | 0.258 (95%CrI 0.117 - 0.48) | Uniform (0.05, 1) | Uninformative prior – estimated through calibrating to % ever and currently homeless |
| Proportion of those released from prison to stable accommodation if never homeless before | 0.917 (95%CrI 0.729 - 0.993) | 0.777 (95%CrI 0.487 - 0.957) | 0.714 (95%CrI 0.301 - 0.973) | 0.797 (95%CrI 0.59 - 0.966) | Uniform (0,1) | Uninformative prior – estimated through calibration to OR of homelessness in recent vs non-recent incarcerated |
| Proportion of those released from prison to stable accommodation if homeless before | 0.556 (95%CrI 0.396 - 0.731) | 0.585 (95%CrI 0.427 - 0.747) | 0.653 (95%CrI 0.515 - 0.795) | 0.596 (95%CrI 0.405 - 0.748) | Uniform (0,1) | Uninformative prior – estimated through calibration to OR of homelessness in recent vs non-recent incarcerated |

**Supplementary Table 16d** Harm reduction parameters for OAT (or drug treatment) and NSP

|  | **Northeast and Cumbria Posterior range** | **Bristol and Severn Posterior range** | **Nottingham Posterior ranges** | **Greater Manchester Posterior ranges** | **Prior Distribution** | **Source** |
| --- | --- | --- | --- | --- | --- | --- |
| Rate of initiation into drug treatment per year | 3.803 (95%CrI 2.776 - 4.867) | 3.665 (95%CrI 2.689 - 4.754) | 4.176 (95%CrI 3.059 - 4.957) | 4.866 (95%CrI 3.537 - 5.866) | Uniform (0, 5) except Manchester Uniform(0, 6) | Uninformative prior– estimated by calibrating to coverage OAT |
| Rate of initiation into using NSP per yr | 4.883 (95%CrI 3.312 - 5.938) | 4.563 (95%CrI 2.812 - 5.871) | 4.813 (95%CrI 2.83 - 5.946) | 4.553 (95%CrI 2.531 - 5.923) | Uniform (0,6) | Uninformative prior – estimated by calibrating to coverage NSP |
| Duration on drug treatment in community or in prison (yrs) | 0.678 (95%CrI 0.612 - 0.726) | 0.695 (95%CrI 0.641 - 0.73) | 0.688 (95%CrI 0.609 - 0.727) | 0.669 (95%CrI 0.619 - 0.716) | Uniform (0.6, 0.733) | 8 months average duration ^29^ |
| Duration on NSP (yrs) | 2.033 (95%CrI 1.895 - 2.224) | 2.064 (95%CrI 1.909 - 2.223) | 2.013 (95%CrI 1.894 - 2.198) | 2.212 (95%CrI 2.015 - 2.295) | Uniform (1.881, 2.299) | 2.09 years average duration ^28^ |
| Duration on drug treatment if homeless (yrs) | 0.502 (95%CrI 0.46 - 0.544) | 0.477 (95%CrI 0.453 - 0.515) | 0.476 (95%CrI 0.451 - 0.519) | 0.514 (95%CrI 0.481 - 0.541) | Uniform (0.45, 0.55) | 6 months average duration, assumption +/- 25% of mean value if not homeless |
| Rate of initiating drug treatment in prison per year | 2.963 (95%CrI 1.679 - 4.661) | 2.167 (95%CrI 0.709 - 3.746) | 2.706 (95%CrI 0.951 - 4.79) | 3.098 (95%CrI 2.068 - 4.699) | Uniform (0,5) | Same distributions as in community, sampled separately |
| Proportion retained on OAT upon incarceration or release | 0.575 (95%CrI 0.414 - 0.816) | 0.741 (95%CrI 0.499 - 0.91) | 0.655 (95%CrI 0.436 - 0.89) | 0.722 (95%CrI 0.46 - 0.878) | Uniform (0,1) | Uninformative prior – estimated through calibration to OAT coverage in prison |
| Proportion who immediately enter NSP upon release | 0.491 (95%CrI 0.142 - 0.854) | 0.593 (95%CrI 0.312 - 0.919) | 0.435 (95%CrI 0.026 - 0.934) | 0.718 (95%CrI 0.138 - 0.972) | Uniform (0,1) | Uninformative prior |
| RR for becoming incarcerated if on OAT | 0.822 (95%CrI 0.743 - 0.881) | 0.791 (95%CrI 0.717 - 0.863) | 0.785 (95%CrI 0.718 - 0.865) | 0.782 (95%CrI 0.734 - 0.875) | Truncated at CI Lognormal  0.79 (0.70,0.89) | ^30^ |
| RR of overdose mortality in first 1-2 weeks after prison release compared with 5-12 weeks in community | 7.449 (95%CrI 4.904 - 9.726) | 8.455 (95%CrI 6.088 - 9.88) | 6.466 (95%CrI 3.899 - 9.474) | 4.932 (95%CrI 3.508 - 6.317) | Uniform (3.2,10) | ^31^ |
| RR of overdose mortality in first 3-4 weeks after prison release compared with 5-12 in community | 1.829 (95%CrI 1.368 - 2.162) | 1.636 (95%CrI 1.354 - 2.002) | 1.913 (95%CrI 1.482 - 2.166) | 1.867 (95%CrI 1.577 - 2.144) | Truncated at CI  Lognormal  1.7 (1.3,2.2) | ^31^ Average of 1-2 week and 3-4 week mortality used for increase in mortality for 4 wks upon release |
| RR of mortality after prison release if on OAT before release | 0.252 (95%CrI 0.149 - 0.378) | 0.256 (95%CrI 0.152 - 0.388) | 0.322 (95%CrI 0.193 - 0.408) | 0.315 (95%CrI 0.192 - 0.405) | Truncated at CI  Lognormal 0.25(0.14,0.45) | ^20^ |
| RR of overdose mortality if on OAT outside of prison | 0.299 (95%CrI 0.219 - 0.355) | 0.296 (95%CrI 0.204 - 0.353) | 0.256 (95%CrI 0.184 - 0.333) | 0.327 (95%CrI 0.264 - 0.358) | Truncated at CI  Lognormal 0.25(0.18,0.36) | ^32^ |
| RR of overdose mortality on entry to OAT | 2.675 (95%CrI 1.462 - 4.015) | 3.408 (95%CrI 2.131 - 4.049) | 2.67 (95%CrI 1.305 - 3.735) | 2.825 (95%CrI 1.87 - 3.703) | Lognormal 1.97(0.94,4.10) | ^32^ |
| RR of overdose mortality on exit OAT | 2.689 (95%CrI 1.903 - 3.616) | 3.054 (95%CrI 2.312 - 3.675) | 2.791 (95%CrI 2.016-3.66) | 3.101 (95%CrI 1.784 - 3.606) | Lognormal 2.38(1.51,3.74) | ^32^ |
| RR of overdose mortality on OAT | 0.319 (95%CrI 0.283 - 0.37) | 0.341 (95%CrI 0.297 - 0.384) | 0.333 (95%CrI 0.291-0.382) | 0.367 (95%CrI 0.323 - 0.389) | Lognormal 0.33(0.28,0.39) | ^33^ |

**Supplementary Table 16e** HCV transmission related parameters and initial infection prevalence

|  | **Northeast and Cumbria Posterior range** | **Bristol and Severn Posterior range** | **Nottingham Posterior ranges** | **Greater Manchester Posterior ranges** | **Prior Distribution** | **Source** |
| --- | --- | --- | --- | --- | --- | --- |
| Infection rate in community PWID not accessing harm reduction, homeless or recently released from prison | 0.602 (95%CrI 0.39 - 0.904) | 0.63 (95%CrI 0.42 - 0.913) | 0.548 (95%CrI 0.331 - 0.817) | 0.674 (95%CrI 0.468 - 0.878) | Uniform (0,1) | Uninformative prior |
| Proportion of primary infections which spontaneously clear | 0.381 (95%CrI 0.293 - 0.488) | 0.45 (95%CrI 0.375 - 0.503) | 0.418 (95%CrI 0.316 - 0.495) | 0.34 (95%CrI 0.287 - 0.453) | Uniform (0.23,0.509) | ^34-36^ |
| Proportion of infections which spontaneously clear in those who previously cleared | 0.816 (95%CrI 0.658 - 0.968) | 0.786 (95%CrI 0.648 - 0.905) | 0.926 (95%CrI 0.81 - 0.996) | 0.897 (95%CrI 0.815 - 0.975) | Uniform (0.6225,1) | ^3^ |
| Relative risk of HCV transmission if on drug treatment (OAT) and not accessing NSP | 0.465 (95%CrI 0.403 - 0.552) | 0.514 (95%CrI 0.427 - 0.612) | 0.53 (95%CrI 0.454 - 0.603) | 0.581 (95%CrI 0.503 - 0.627) | 0.5 (95%CI 0.4, 0.63) | ^37^ |
| Relative risk of HCV transmission if accessing NSP but not on drug treatment (OAT) | 0.602 (95%CrI 0.372 - 0.776) | 0.468 (95%CrI 0.29 - 0.653) | 0.586 (95%CrI 0.297 - 0.747) | 0.398 (95%CrI 0.275 - 0.585) | 0.44 (95%CI 0.2, 0.8) | ^37^ |
| Relative risk of HCV transmission if accessing both NSP and drug treatment (OAT) | 0.246 (95%CrI 0.083 - 0.476) | 0.321 (95%CrI 0.091 - 0.599) | 0.516 (95%CrI 0.28 - 0.78) | 0.385 (95%CrI 0.195 - 0.699) | 0.26 (95%CI 0.07, 0.89) | ^37^ |
| Relative risk of HCV transmission if currently homeless | 1.698 (95%CrI 1.13 - 2.317) | 1.66 (95%CrI 1.093 - 2.163) | 1.624 (95%CrI 1.116 - 2.374) | 1.734 (95%CrI 1.322 - 2.367) | Uniform (1, 2.59) | Subgroup analysis for Western Europe ^38^ |
| Relative risk of HCV transmission if recently released from prison | 1.428 (95%CrI 1.049 - 1.878) | 1.237 (95%CrI 1.016 - 1.645) | 1.691 (95%CrI 1.065 - 2.008) | 1.629 (95%CrI 1.362 - 1.982) | Uniform (1, 2.04) | Increased risk if recently released ^39^ |
| Relative risk of HCV transmission if currently incarcerated | 0.819 (95%CrI 0.065 - 1.934) | 1.6 (95%CrI 0.725 - 2.463) | 0.608 (95%CrI 0.056 - 1.623) | 1.135 (95%CrI 0.352 - 2.237) | Uniform (0,3) | Uninformative prior |
| HCV Ab prevalence for initial conditions (2000) | 0.368 (95%CrI 0.27 - 0.483) | 0.403 (95%CrI 0.308 - 0.547) | 0.409 (95%CrI 0.276 - 0.541) | 0.547 (95%CrI 0.415 - 0.634) | Uniform (0.30, 0.65) Bristol and Manchester  Uniform (0.2, 0.5) Northeast  Uniform (0.2, 0.55)  Nottingham | Based on prevalence data from UAM for 2011 |

**Supplementary Table 17a** Non ODN specific priors for testing and treatment parameters (using data up to end of 2022)

| **Parameter** | **Year** | **Northeast and Cumbria Posteriors** | **Bristol and Severn Posteriors** | **Nottingham Posteriors** | **Greater Manchester Posteriors** | **Prior** | **Source** |
| --- | --- | --- | --- | --- | --- | --- | --- |
| **DRUG TREATMENT CENTRE ESTIMATES (DTC)** | | | | | | | |
| Factor reduction in testing prior to 2016 | 2005-2015 | 0.166 (95%CrI 0.163 - 0.169) | 0.166 (95%CrI 0.164 - 0.169) | 0.165 (95%CrI 0.163 - 0.169) | 0.166 (95%CrI 0.165 - 0.169) | Uniform (0.1626, 0.1694) | For DTC we have 38431 tests over 2005-2014 (10 years^6^) and in 2015-2016 we have 46309 tests (sentinel surveillance data), implying a relative rate of testing in 2005-2014 of (38431/5)/46309=16.6%. Use 95% CI as bounds for the uniform distribution. |
| % linked to treatment from testing in DTC | 2005-2014 | 0.103 (95%CrI 0.096 - 0.11) | 0.104 (95%CrI 0.097 - 0.11) | 0.103 (95%CrI 0.097 - 0.11) | 0.104 (95%CrI 0.099 - 0.111) | Uniform (0.0951, 0.1120) | 10.4% use 95%CI as bounds for uniform distribution ^6^ |
|  | 2015-2016 | 0.543 (95%CrI 0.528 - 0.565) | 0.542 (95%CrI 0.529 - 0.563) | 0.546 (95%CrI 0.53 - 0.567) | 0.554 (95%CrI 0.537 - 0.564) | Uniform (0.5276, 0.5688) | 1229/2242 from linked datasets, use 95%CI as bounds for uniform distribution |
|  | 2017-2018 | 0.586 (95%CrI 0.57 - 0.606) | 0.597 (95%CrI 0.574 - 0.61) | 0.588 (95%CrI 0.568 - 0.604) | 0.588 (95%CrI 0.571 - 0.601) | Uniform (0.5667, 0.6108) | 1128/1916 from linked datasets, use 95%CI as bounds for uniform distribution |
|  | 2019 onwards | 0.753 (95%CrI 0.579 - 0.935) | 0.703 (95%CrI 0.543 - 0.891) | 0.712 (95%CrI 0.513 - 0.921) | 0.763 (95%CrI 0.599 - 0.877) | Uniform (0.5073, 0.9) | 2003/3829 from linked datasets, use 95%CI as lower bounds for uniform distribution, upper bound increased due to possible censoring |
| Rate of initiating treatment following diagnosis in DTC (only among those that are linked to treatment) | 2005-2016 | 0.215 (95%CrI 0.209 - 0.218) | 0.214 (95%CrI 0.208 - 0.218) | 0.212 (95%CrI 0.204 - 0.218) | 0.212 (95%CrI 0.206 - 0.216) | Uniform (0.2036, 0.2188) | Linked dataset gives time to treatment 3 mths 66 people, 3-6 mths 54 people, 6-12 mths 120 people, >12 mths 1000 people. See footnote ~ for creation of this type of prior |
|  | 2017-2018 | 1.08 (95%CrI 0.975 - 1.214) | 1.158 (95%CrI 1.038 - 1.254) | 1.107 (95%CrI 0.994 - 1.212) | 1.131 (95%CrI 1.019 - 1.204) | Uniform (0.9579, 1.2682) | Linked dataset gives time to treatment 3mths 313, people, 3-6 mths 187 people, 6-12 mths 210 people, > 12 mths 442 people. footnote ~ for creation of this type of prior |
|  | 2019 onwards | 1.979 (95%CrI 1.731 - 2.219) | 1.936 (95%CrI 1.718 - 2.254) | 2.066 (95%CrI 1.761 - 2.428) | 2.163 (95%CrI 1.905 - 2.336) | Uniform (1.6960, 2.4503) | Linked dataset gives time to treatment 3mths 939 people, 3-6 mths 400 people, 6-12 mths 335 people, > 12 mths 376 people. See footnote ~ for creation of this type of prior |
| Proportion achieving SVR in DTC | 2005-2014 | 0.52 (95%CrI 0.488 - 0.563) | 0.526 (95%CrI 0.491 - 0.559) | 0.521 (95%CrI 0.493 - 0.561) | 0.516 (95%CrI 0.496 - 0.543) | Uniform (0.4860, 0.5721) | 273/516 ^6^, use 95%CI as uniform distribution bounds |
|  | 2015-2016 | 0.811 (95%CrI 0.793 - 0.83) | 0.819 (95%CrI 0.799 - 0.834) | 0.823 (95%CrI 0.795 - 0.835) | 0.797 (95%CrI 0.791 - 0.821) | Uniform (0.7902, 0.8359) | 909/1118 from linked datasets, use 95% CI as bounds for the uniform distribution |
|  | 2017-2018 | 0.802 (95%CrI 0.784 - 0.824) | 0.799 (95%CrI 0.784 - 0.814) | 0.81 (95%CrI 0.79 - 0.829) | 0.812 (95%CrI 0.79 - 0.823) | Uniform (0.7827, 0.8309) | 831/1030 from linked datasets, use 95% CI as bounds for the uniform distribution |
|  | 2019 onwards | 0.748 (95%CrI 0.731 - 0.762) | 0.733 (95%CrI 0.72 - 0.752) | 0.735 (95%CrI 0.72 - 0.757) | 0.748 (95%CrI 0.728 - 0.758) | Uniform (0.7190, 0.7628) | 1138/1536 from linked datasets, use 95% CI as bounds for the uniform distribution |
| **PRISON ESTIMATES** | | | | | | | |
| Factor reduction in testing in prison prior to 2016 | 2005-2015 | 0.546 (95%CrI 0.538 - 0.552) | 0.548 (95%CrI 0.541 - 0.553) | 0.547 (95%CrI 0.538 - 0.553) | 0.542 (95%CrI 0.538 - 0.551) | Uniform (0.5368, 0.5538) | For prison, we have 36087 tests over 2005-2014 (10 years^6^) and 13236 over 2015-2016 (sentinel surveillance data), implying a relative rate of testing in 2005-2014 of (36087/5)/13236=54.5%. Use 95% CI as bounds for the uniform distribution. |
| % linked to treatment from testing in prison | 2005-2014 | 0.115 (95%CrI 0.103 - 0.121) | 0.104 (95%CrI 0.099 - 0.113) | 0.111 (95%CrI 0.099 - 0.121) | 0.108 (95%CrI 0.103 - 0.117) | Uniform (0.0986, 0.1219) | 306/2776 ^6^, use 95%CI as bounds for uniform distribution |
|  | 2015-2016 | 0.514 (95%CrI 0.493 - 0.541) | 0.52 (95%CrI 0.496 - 0.543) | 0.513 (95%CrI 0.493 - 0.543) | 0.522 (95%CrI 0.495 - 0.537) | Uniform (0.4914, 0.5507) | 569/1092 from linked datasets, use 95%CI as bounds for uniform distribution |
|  | 2017-2018 | 0.576 (95%CrI 0.558 - 0.591) | 0.579 (95%CrI 0.565 - 0.591) | 0.582 (95%CrI 0.561 - 0.594) | 0.581 (95%CrI 0.567 - 0.59) | Uniform (0.5569, 0.5952) | 1477/2564 from linked datasets, use 95%CI as bounds for uniform distribution |
| Rate of initiating treatment following diagnosis in prison | 2005-2016 | 0.284 (95%CrI 0.26 - 0.313) | 0.294 (95%CrI 0.273 - 0.313) | 0.298 (95%CrI 0.27 - 0.314) | 0.265 (95%CrI 0.253 - 0.289) | Uniform (0.2517, 0.3164) | Linked dataset gives time to treatment 3mths 35 people, 3-6 mths 49 people, 6-12 mths 53 people, > 12 mths 437 people. See footnote ~ for creation of this type of prior |
|  | 2017-2018 | 1.15 (95%CrI 1.025 - 1.285) | 1.195 (95%CrI 1.03 - 1.385) | 1.229 (95%CrI 1.035 - 1.439) | 1.145 (95%CrI 1.04 - 1.366) | Uniform (1.0138, 1.4451) | Linked dataset gives time to treatment 3mths 473 people, 3-6 mths 246 people, 6-12 mths 275 people, > 12 mths 566 people. See footnote ~ for creation of this type of prior |
|  | 2019 onwards | 2.716 (95%CrI 2.165 - 3.279) | 2.727 (95%CrI 2.137 - 3.243) | 2.809 (95%CrI 2.012 - 3.361) | 3.046 (95%CrI 2.086 - 3.367) | Uniform (1.9228, 3.3905) | Linked dataset gives time to treatment 3mths 1126 people, 3-6 mths 310 people, 6-12 mths 246 people, >12 mths 288 people. See footnote ~ for creation of this type of prior |
| Proportion achieving SVR in prison | 2005-2014 | 0.414 (95%CrI 0.372 - 0.452) | 0.434 (95%CrI 0.37 - 0.468) | 0.426 (95%CrI 0.369 - 0.467) | 0.422 (95%CrI 0.374 - 0.448) | Uniform (0.3630, 0.4736) | 128/306^6^, use 95%CI as uniform distribution bounds |
|  | 2015-2016 | 0.829 (95%CrI 0.806 - 0.856) | 0.823 (95%CrI 0.803 - 0.847) | 0.822 (95%CrI 0.801 - 0.843) | 0.844 (95%CrI 0.817 - 0.859) | Uniform (0.7991, 0.8637) | 429/516 linked datasets, use confidence intervals as uniform distribution bounds |
|  | 2017-2018 | 0.791 (95%CrI 0.771 - 0.805) | 0.774 (95%CrI 0.763 - 0.791) | 0.787 (95%CrI 0.764 - 0.802) | 0.792 (95%CrI 0.771 - 0.803) | Uniform (0.7621, 0.8063) | 1043/1330 from linked datasets, use 95% CI as bounds for the uniform distribution |
|  | 2019 onwards | 0.739 (95%CrI 0.718 - 0.756) | 0.73 (95%CrI 0.716 - 0.747) | 0.732 (95%CrI 0.716 - 0.759) | 0.745 (95%CrI 0.733 - 0.755) | Uniform (0.7156, 0.7596) | 1130/1532 from linked datasets, use 95% CI as bounds for the uniform distribution |
| **OTHER SETTING ESTIMATES** | | | | | | | |
| % linked to treatment from testing in other settings | 2005-2014 | 0.252 (95%CrI 0.247 - 0.258) | 0.255 (95%CrI 0.251 - 0.258) | 0.253 (95%CrI 0.248 - 0.258) | 0.253 (95%CrI 0.248 - 0.257) | Uniform (0.2467, 0.2583) | 5504/21797 (Simmons et al., 2018), use 95%CI as bounds for uniform distribution |
|  | 2015-2016 | 0.526 (95%CrI 0.516 - 0.534) | 0.52 (95%CrI 0.514 - 0.529) | 0.519 (95%CrI 0.514 - 0.529) | 0.523 (95%CrI 0.518 - 0.533) | Uniform (0.5135, 0.5349) | 4382/8360 linked data, use 95%CI as bounds for uniform distribution |
|  | 2017-2018 | 0.603 (95%CrI 0.593 - 0.612) | 0.596 (95%CrI 0.59 - 0.603) | 0.602 (95%CrI 0.59 - 0.612) | 0.599 (95%CrI 0.591 - 0.608) | Uniform (0.5895,0.6125) | 4191/6973 linked data, use 95%CI as bounds for uniform distribution |
|  | 2019 onwards | 0.551 (95%CrI 0.539 - 0.56) | 0.546 (95%CrI 0.538 - 0.555) | 0.553 (95%CrI 0.539 - 0.561) | 0.547 (95%CrI 0.541 - 0.558) | Uniform (0.5367, 0.5624) | 3176/5779 linked data, use 95%CI as bounds for uniform distribution |
| Rate of initiating treatment following diagnosis in other settings | 2005-2016 | 0.407 (95%CrI 0.4 - 0.415) | 0.409 (95%CrI 0.401 - 0.416) | 0.406 (95%CrI 0.4 - 0.415) | 0.404 (95%CrI 0.4 - 0.414) | Uniform (0.3993, 0.4183) | Linked dataset gives time to treatment 3mths 564 people, 3-6 mths 477 people, 6-12 mths 829 people, > 12 mths 3810 people. See footnote ~ for creation of this type of prior |
|  | 2017-2018 | 1.309 (95%CrI 1.163 - 1.491) | 1.334 (95%CrI 1.179 - 1.527) | 1.273 (95%CrI 1.148 - 1.429) | 1.477 (95%CrI 1.254 - 1.589) | Uniform (1.1366, 1.5957) | Linked dataset gives time to treatment 3mths 2083 people, 3-6 months 1100 people, 6-12 mths 1117 people, greater than 12 mths 2032 people. See footnote ~ for creation of this type of prior |
|  | 2019 onwards | 2.642 (95%CrI 2.202 - 3.005) | 2.675 (95%CrI 2.198 - 2.98) | 2.516 (95%CrI 2.073 - 2.883) | 2.32 (95%CrI 2.079 - 2.948) | Uniform (1.9733, 3.0716) | Linked dataset gives time to treatment 3mths 3594 people, 3-6 mths 1247 people, 6-12 mths 932 people, >12 mths 932 people. See footnote ~ for creation of this type of prior |
| Proportion achieving SVR in other settings | 2005-2014 | 0.498 (95%CrI 0.485 - 0.508) | 0.498 (95%CrI 0.489 - 0.507) | 0.492 (95%CrI 0.483 - 0.506) | 0.501 (95%CrI 0.485 - 0.508) | Uniform (0.4826, 0.5090) | 2729/5504 (Simmons et al., 2018), use 95%CI as uniform distribution bounds |
|  | 2015-2016 | 0.923 (95%CrI 0.917 - 0.931) | 0.92 (95%CrI 0.916 - 0.926) | 0.925 (95%CrI 0.917 - 0.931) | 0.92 (95%CrI 0.917 - 0.926) | Uniform (0.9150, 0.9312) | 3843/4163 linked datasets, use 95%CI as uniform distribution bounds |
|  | 2017-2018 | 0.892 (95%CrI 0.882 - 0.899) | 0.888 (95%CrI 0.881 - 0.896) | 0.891 (95%CrI 0.882 - 0.898) | 0.892 (95%CrI 0.885 - 0.899) | Uniform (0.8804, 0.9001) | 3472/3900 linked datasets use 95%CI as uniform distribution bounds |
|  | 2019 onwards | 0.816 (95%CrI 0.803 - 0.824) | 0.811 (95%CrI 0.799 - 0.823) | 0.803 (95%CrI 0.795 - 0.817) | 0.8 (95%CrI 0.795 - 0.815) | Uniform (0.7946, 0.8245) | 2146/2651 linked datasets, use 95%CI as uniform distribution bounds |

~Calculate the instantaneous rates that corresponds to each set of patients getting treated. For example, for the following data: 66 in 3 months, 54 in 3-6 months, 120 in 6-12 months, 1000 after 12 months. Calculate the instantaneous rates (-log(1- proportion)/time in years) for 66/1240 in 3 months, 120/1240 in 6 months, 240 in 1 year. Use the minimum and maximum values from these three calculations as the bounds for a uniform distribution.

**Supplementary Table 17b** Bristol and Severn specific priors for treatment pathway parameters

| **Parameter** | **Year** | **Posterior** | **Prior** | **Source** |
| --- | --- | --- | --- | --- |
| Diagnosis rate in DTC | 2017 onwards | 1.038 (95%CrI 0.78 - 1.178) | Uniform (0.7, 1.2) | Assumption – estimated in the model calibration to give the treatment numbers for each year in prior table |
| Diagnosis rate in DTC | 2016 | 0.241 (95%CrI 0.068 - 0.44) | Uniform (0.01, 0.5) | Assumption – estimated in the model calibration to give the treatment numbers for each year in prior table |
| Adjustment factor for Bristol ODN for % linked to treatment in DTC | 2015 onwards | 0.998 (95%CrI 0.902 - 1.047) | Uniform (0.8462, 1.0501) | 230/448 - proportion linked to treatment from DTC in Bristol ODN all years (numerator), 4003/7382 - proportion linked to treatment from DTC in all ODNs all years (denominator)  See footnote for creation of this type of prior * |
| Adjustment factor for Bristol ODN for proportion achieving SVR in DTC | 2015 onwards | 1.181 (95%CrI 1.078 - 1.212) | Uniform (1.0562, 1.2141) | 148/167 – overall SVR for DTC in Bristol ODN all years (numerator), 2645/3385 - overall SVR for DTC in all ODN all years - denominator. See footnote for creation of this type of prior * |
| Diagnosis rate in prison | 2017 onwards | 0.759 (95%CrI 0.516 - 1.391) | Uniform (0.5, 2) | Assumption to take into account increased opt out testing on reception to prison and high intensity test and treat events |
| Diagnosis rate in prison | 2016 | 0.258 (95%CrI 0.075 - 0.465) | Uniform (0.01, 0.5) | Assumption – estimated in the model calibration to give the treatment numbers for each year in prior table |
| % linked to treatment from testing in prison | 2019 onwards | 0.681 (95%CrI 0.548 - 0.879) | Uniform (0.5, 0.95) | Assumption to take into account high intensity test and treat increasing linkage to care as well as increasing upper bound due to possible censoring of data |
| Adjustment factor for Bristol ODN for % linked to treatment from testing in prison | 2015 onwards | 0.853 (95%CrI 0.791 - 0.902) | Uniform (0.7184, 0.9065) | 214/466 - proportion linked to treatment from prison in Bristol ODN all years (numerator), 3422/6045 - proportion linked to treatment from prison in all ODNs all years (denominator). See footnote for creation of this type of prior* |
| Adjustment factor for Bristol ODN for proportion achieving SVR in prison | 2015 onwards | 1.022 (95%CrI 0.977 - 1.104) | Uniform (0.9739, 1.1594) | 143/174– overall SVR for prison in Bristol ODN all years (numerator), 2295/2973 - overall SVR for prison in all ODN all years (denominator). See footnote for creation of this type of prior * |
| Diagnosis rate in other settings | 2017 onwards | 0.221 (95%CrI 0.066 - 0.372) | Uniform (0.01, 0.5) | Assumption – estimated in the model calibration to give the treatment numbers for each year in prior table |
| Diagnosis rate in other settings | 2016 | 0.169 (95%CrI 0.031 - 0.385) | Uniform (0.01, 0.5) | Assumption – estimated in the model calibration to give the treatment numbers for each year in prior table |
| Diagnosis rate in other settings | 2005-2015 | 0.209 (95%CrI 0.127 - 0.248) | Uniform (0.01, 0.25) | Assumption, restricted to 0.25 after checking posteriors on intermediate ABC calibrations |
| Adjustment factor for Bristol ODN for % linked to treatment in other settings | 2015 onwards | 1.02 (95%CrI 0.978 - 1.052) | Uniform (0.9716, 1.0720) | 954/1593 - proportion linked to treatment from other settings in Bristol ODN all years (numerator), numerator Bristol ODN data, 11339/19364 - proportion linked to treatment from other settings in all ODNs all years (denominator). See footnote for creation of this type of prior* |
| Adjustment factor for Bristol ODN for proportion achieving SVR in other settings | 2015 onwards | 1.046 (95%CrI 1.025 - 1.063) | Uniform (1.0092, 1.0648) | 749/818 – overall SVR for other settings in Bristol ODN all years (numerator), 9154/10363 - overall SVR for other settings in all ODN all years - denominator. See footnote for creation of this type of prior * |

*The numerator and denominator proportions were sampled from uniform distributions (bounds were confidence intervals for the proportions) and the fraction calculated 1000 times. The minimum and maximum values of this fraction were then used as the bounds for the prior uniform distribution.

**Supplementary Table 17c** Greater Manchester specific priors for treatment pathway parameters

| **Parameter** | **Year** | **Posterior** | **Prior** | **Source** |
| --- | --- | --- | --- | --- |
| Diagnosis rate in DTC | 2017 onwards | 0.412 (95%CrI 0.172 - 0.663) | Uniform (0.1, 1.0) | Assumption – estimated in the model calibration to give the treatment numbers for each year |
| Diagnosis rate in DTC | 2016 | 0.143 (95%CrI 0.019 - 0.418) | Uniform (0.01, 0.5) | Assumption – estimated in the model calibration to give the treatment numbers for each year |
| Adjustment factor for Greater Manchester ODN for % linked to treatment in DTC | 2015 onwards | 1.143 (95%CrI 1.099 - 1.194) | Uniform (1.05, 1.21) | 565/921 - proportion linked to treatment from DTC in Manchester ODN all years (numerator), 4003/7382 - proportion linked to treatment from DTC in all ODNs all years (denominator). See footnote for creation of this type of prior* |
| Adjustment factor for Greater Manchester ODN SVR in DTC | 2015 onwards | 0.969 (95%CrI 0.937 - 1.009) | Uniform (0.88, 1.01) | 344/465 – overall SVR for DTC in Manchester ODN all years (numerator), 2645/3385 - overall SVR for DTC in all ODN all years - denominator. See footnote for creation of this type of prior * |
| Diagnosis rate in prison | 2017 onwards | 1.218 (95%CrI 0.871 - 1.713) | Uniform (0.5, 2) | Assumption to take into account increased opt out testing on reception to prison and high intensity test and treat events |
|  | 2016 | 0.102 (95%CrI 0.013 - 0.357) | Uniform (0.01, 0.5) | Assumption - – estimated in the model calibration to give the treatment numbers for each year |
| % linked to treatment from testing in prison | 2019 onwards | 0.847 (95%CrI 0.702 - 0.945) | Uniform (0.5, 0.95) | Assumption to take into account high intensity test and treat increasing linkage to care as well as increasing upper bound due to possible censoring of data |
| Adjustment factor for Greater Manchester ODN for % linked to treatment from testing in prison | 2015 onwards | 1.329 (95%CrI 1.251 - 1.402) | Uniform (1.22, 1.42) | 289/399 - proportion linked to treatment from prison in Manchester ODN all years (numerator), 3422/6045 - proportion linked to treatment from prison in all ODNs all years (denominator). See footnote for creation of this type of prior* |
| Adjustment factor for Greater Manchester ODN for proportion achieving SVR in prison | 2015 onwards | 0.975 (95%CrI 0.922 - 1.044) | Uniform (0.90, 1.07) | 195/256 – overall SVR for prison in Bristol ODN all years (numerator), 2295/2973 - overall SVR for prison in all ODN all years (denominator). See footnote for creation of this type of prior * |
| Diagnosis rate in other settings | 2017 onwards | 0.034 (95%CrI 0.011 - 0.067) | Uniform (0.01, 0.2) | Assumption, restricted to 0.2 after checking posteriors on intermediate ABC calibrations |
| Diagnosis rate in other settings | 2016 | 0.11 (95%CrI 0.018 - 0.248) | Uniform (0.01, 0.5) | Assumption – estimated in the model calibration to give the treatment numbers for each year in prior table |
| Diagnosis rate in other settings | 2005-2015 | 0.181 (95%CrI 0.063 - 0.37) | Uniform (0.01, 0.5) | Uninformative prior |
| Adjustment factor for Greater Manchester ODN for % linked to treatment in other settings | 2015 onwards | 1.072 (95%CrI 1.023 - 1.097) | Uniform (1.00, 1.11) | 733/1182 - proportion linked to treatment from other settings in Manchester ODN all years (numerator), 11339/19364 - proportion linked to treatment from other settings in all ODNs all years (denominator). See footnote for creation of this type of prior* |
| Adjustment factor for Greater Manchester ODN for proportion achieving SVR in other settings | 2015 onwards | 1.06 (95%CrI 1.024 - 1.076) | Uniform (1.02, 1.08) | 634/685 – overall SVR for other settings in Manchester ODN all years (numerator), 9154/10363 - overall SVR for other settings in all ODN all years - denominator. See footnote for creation of this type of prior * |

*The numerator and denominator proportions were sampled from uniform distributions (bounds were confidence intervals for the proportions) and the fraction calculated 1000 times. The minimum and maximum values of this fraction were then used as the bounds for the prior uniform distribution.

**Supplementary Table 17d** Northeast and Cumbria specific priors for treatment pathway parameters

| **Parameter** | **Year** | **Posterior** | **Prior** | **Source** |
| --- | --- | --- | --- | --- |
| Diagnosis rate in DTC | 2017 onwards | 0.318 (95%CrI 0.145 - 0.476) | Uniform (0.01, 0.6) | Assumption – estimated in the model calibration to give the treatment numbers for each year |
| Diagnosis rate in DTC | 2016 | 0.27 (95%CrI 0.077 - 0.46) | Uniform (0.01, 0.5) | Assumption – estimated in the model calibration to give the treatment numbers for each year |
| Adjustment factor for Northeast and Cumbria ODN for % linked to treatment | 2015 onwards | 0.73 (95%CrI 0.681 - 0.767) | Uniform (0.65, 0.77) | 446/1162 - proportion linked to treatment from DTC in Northeast ODN all years (numerator), 4003/7382 - proportion linked to treatment from DTC in all ODNs all years (denominator). See footnote for creation of this type of prior* |
| Adjustment factor for Northeast and Cumbria ODN for proportion achieving SVR in DTC | 2015 onwards | 0.945 (95%CrI 0.898 - 0.993) | Uniform (0.87, 1.00) | 332/454 – overall SVR for DTC in Northeast ODN all years (numerator), 2645/3385 - overall SVR for DTC in all ODN all years - denominator. See footnote for creation of this type of prior * |
| Diagnosis rate in prison | 2017 onwards | 0.789 (95%CrI 0.518 - 1.469) | Uniform (0.5, 2) | Assumption to take into account increased opt out testing on reception to prison and high intensity test and treat events |
|  | 2016 | 0.328 (95%CrI 0.128 - 0.488) | Uniform (0.01, 0.5) | Assumption– estimated in the model calibration to give the treatment numbers for each year |
| % linked to treatment from testing in prison | 2019 onwards | 0.688 (95%CrI 0.522 - 0.872) | Uniform (0.5, 0.95) | Assumption to take into account high intensity test and treat increasing linkage to care as well as increasing upper bound due to possible censoring of data |
| Adjustment factor for Northeast and Cumbria ODN for % linked to treatment from testing in prison | 2015 onwards | 0.854 (95%CrI 0.812 - 0.901) | Uniform (0.81, 0.93) | 722/1471 - proportion linked to treatment from prison in Northeast ODN all years (numerator), 3422/6045 - proportion linked to treatment from prison in all ODNs all years (denominator). See footnote for creation of this type of prior* |
| Adjustment factor for Northeast and Cumbria ODN for proportion achieving SVR in prison | 2015 onwards | 1.005 (95%CrI 0.97 - 1.048) | Uniform (0.97, 1.08) | 543/687 – overall SVR for prison in Bristol ODN all years (numerator), 2295/2973 - overall SVR for prison in all ODN all years (denominator). See footnote for creation of this type of prior * |
| Diagnosis rate in other settings | 2017 onwards | 0.084 (95%CrI 0.015 - 0.206) | Uniform (0.01, 0.5) | Assumption – estimated in the model calibration to give the treatment numbers for each year in prior table |
| Diagnosis rate in other settings | 2016 | 0.341 (95%CrI 0.112 - 0.486) | Uniform (0.01, 0.5) | Assumption – estimated in the model calibration to give the treatment numbers for each year in prior table |
| Diagnosis rate in other settings | 2005-2015 | 0.309 (95%CrI 0.095 - 0.476) | Uniform (0.01, 0.5) | Uninformative prior |
| Adjustment factor for Northeast and Cumbria ODN for % linked to treatment in other settings | 2015 onwards | 0.628 (95%CrI 0.591 - 0.682) | Uniform (0.59, 0.69) | 453/1273 - proportion linked to treatment from other settings in Northeast ODN all years (numerator), 11339/19364 - proportion linked to treatment from other settings in all ODNs all years (denominator). See footnote for creation of this type of prior |
| Adjustment factor for Northeast and Cumbria ODN for proportion achieving SVR in other settings | 2015 onwards | 0.857 (95%CrI 0.812 - 0.895) | Uniform (0.80, 0.90) | 333/442 – overall SVR for other settings in Northeast ODN all years (numerator), 9154/10363 - overall SVR for other settings in all ODN all years - denominator. See footnote for creation of this type of prior * |

*The numerator and denominator proportions were sampled from uniform distributions (bounds were confidence intervals for the proportions) and the fraction calculated 1000 times. The minimum and maximum values of this fraction were then used as the bounds for the prior uniform distribution.

**Supplementary Table 17e** Nottingham specific priors for treatment pathway parameters

| Parameter | Year | Posterior | Prior | Source |
| --- | --- | --- | --- | --- |
| Diagnosis rate in DTC | 2017 onwards | 0.688 (95%CrI 0.339 - 1.083) | Uniform (0.2,1.5) | Assumption – estimated in the model calibration to give the treatment numbers for each year |
| Diagnosis rate in DTC | 2016 | 0.157 (95%CrI 0.022 - 0.335) | Uniform (0.01, 0.5) | Assumption – estimated in the model calibration to give the treatment numbers for each year |
| Adjustment factor for Nottingham ODN for % linked to treatment in DTC | 2015 onwards | 0.918 (95%CrI 0.85 - 1.05) | Uniform (0.85, 1.06) | 201/390 - proportion linked to treatment from DTC in Nottingham ODN all years (numerator), 4003/7382 - proportion linked to treatment from DTC in all ODNs all years (denominator). See footnote for creation of this type of prior |
| Adjustment factor for Nottingham ODN for proportion achieving SVR in DTC | 2015 onwards | 1.039 (95%CrI 0.938 - 1.1) | Uniform (0.92, 1.11) | 136/172 – overall SVR for DTC in Nottingham ODN all years (numerator), 2645/3385 - overall SVR for DTC in all ODN all years - denominator. See footnote for creation of this type of prior * |
| Diagnosis rate in prison | 2017 onwards | 0.81 (95%CrI 0.246 - 1.695) | Uniform (0.2, 2) | Assumption– estimated in the model calibration to give the treatment numbers for each year |
|  | 2016 | 0.4 (95%CrI 0.069 - 0.496) | Uniform (0.01, 0.5) |  |
| % linked to treatment from testing in prison | 2019 onwards | 0.811 (95%CrI 0.541 - 0.941) | Uniform (0.5, 0.95) | Assumption to take into account high intensity test and treat increasing linkage to care as well as increasing upper bound due to possible censoring of data |
| Adjustment factor for Nottingham ODN for % linked to treatment from testing in prison | 2015 onwards | 1.205 (95%CrI 1.091 - 1.297) | Uniform (1.07, 1.32) | 159/236 - proportion linked to treatment from prison in Nottingham ODN all years (numerator), 3422/6045 - proportion linked to treatment from prison in all ODNs all years (denominator). See footnote for creation of this type of prior* |
| Adjustment factor for Nottingham ODN for proportion achieving SVR in prison | 2015 onwards | 0.941 (95%CrI 0.887 - 1.049) | Uniform (0.88, 1.10) | 107/140 numerator – overall SVR for prison in Bristol ODN all years (numerator), 2295/2973 - overall SVR for prison in all ODN all years (denominator). See footnote for creation of this type of prior * |
| Diagnosis rate in other settings | 2017 onwards | 0.044 (95%CrI 0.011 - 0.08) | Uniform (0.01, 0.1) | Assumption, restricted to 0.1 after checking posteriors on intermediate ABC calibrations |
|  | 2016 | 0.042 (95%CrI 0.013 - 0.075) | Uniform (0.01, 0.1) | Assumption, restricted to 0.1 after checking posteriors on intermediate ABC calibrations |
|  | 2005-2015 | 0.045 (95%CrI 0.016 - 0.077) | Uniform (0.01, 0.1) | Uninformative prior, restricted to 0.1 after checking posteriors on intermediate ABC calibrations |
| Adjustment factor for Nottingham ODN for % linked to treatment in other settings | 2015 onwards | 1.134 (95%CrI 1.085 - 1.168) | Uniform (1.06, 1.18) | 763/1227 - proportion linked to treatment from other settings in Northeast ODN all years (numerator), 11339/19364 - proportion linked to treatment from other settings in all ODNs all years (denominator). See footnote for creation of this type of prior* |
| Adjustment factor for Nottingham ODN for proportion achieving SVR in other settings | 2015 onwards | 0.978 (95%CrI 0.96 - 1.019) | Uniform (0.96, 1.02) | 630/720– overall SVR for other settings in Northeast ODN all years (numerator), 9154/10363 - overall SVR for other settings in all ODN all years - denominator. See footnote for creation of this type of prior * |

*The numerator and denominator proportions were sampled from uniform distributions (bounds were confidence intervals for the proportions) and the fraction calculated 1000 times. The minimum and maximum values of this fraction were then used as the bounds for the prior uniform distribution.

**Supplementary Table 18**: Additional model parameter distributions for ex-injectors and disease progression

| **Parameter** | **Distribution** | **Source** |
| --- | --- | --- |
| Age at initiation of injecting | Uniform (18-25) | Assumption |
| Life expectancy | Uniform (65-75) | Assumption |
| Reduction in life-expectancy if homeless | Uniform (16-22) | ^40^ |
| Yearly progression rate from F0 to F1 | 0.0529-0.2095 sampled from normal distribution | PWID specific instantaneous rates from Smith et al ^41^ |
| Yearly progression rate from F1 to F2 | 0.0216-0.1013 sampled from normal distribution |  |
| Yearly progression rate from F2 to F3 | 0.0450-0.1145 sampled from normal distribution |  |
| Yearly progression rate from F3 to compensated cirrhosis | 0.0513-0.1838 sampled from normal distribution |  |
| Yearly progression rate from compensated cirrhosis to decompensated cirrhosis | 0.0166-0.0921 | Instantaneous rates calculated from sampled beta distributions of transition probabilities in Shepherd et al ^9^ |
| Yearly progression rate from compensated cirrhosis or decompensated cirrhosis to hepatocellular carcinoma | 0.0003-0.0684 |  |
| Yearly progression rate from decompensated cirrhosis or HCC to liver transplant | 0.0062-0.0962 |  |
| Yearly progression rate from liver transplant to post liver transplant | 1.0423-2.4412 |  |
| Decompensated cirrhosis related death rate per year | 0.1063-0.1842 |  |
| Hepatocellular carcinoma related death rate per year | 0.3904-0.7697 |  |
| Liver transplant related death rate per year | 0.0911-0.4348 |  |
| Post liver transplant related death rate per year | 0.0280-0.1016 |  |
| Relative risk for progression rate from compensated to decompensated cirrhosis following SVR | 0.07 (95%CI 0.03,0.2) | Sampled from transformed lognormal distribution in ^42^ |
| Relative risk for progression rate from compensated cirrhosis to HCC following SVR | 0.23 (95%CI 0.16,0.35) | Sampled from transformed lognormal distribution ^43^ |

Note: the same sampled set of the parameters in this table were used for all four ODNs

**Supplementary** **Table 19: Costs and Utility parameter values**

| **Annual Costs** | **Mean Value £** | **Distribution** | **Source** |
| --- | --- | --- | --- |
| Uninfected | 0 | Constant |  |
| F0 and F1 Mild HCV | 208 | Gamma (0.659,289)*PPI | ^10^  ^44^ |
| F2 and F3 Moderate HCV | 1,064 | Gamma (0.485,2038)*PPI |  |
| Compensated Cirrhosis | 1,669 | Gamma (0.211,7452)*PPI |  |
| Decompensated cirrhosis | 13,531 | Gamma (0.901,13974)*PPI |  |
| Hepatocellular Carcinoma | 12,771 | Gamma (0.926,12251)*PPI |  |
| Liver transplant | 40,047 | Gamma (89.75,304.5)*PPI | ^9^ |
| Post-transplant | 2,040 | Gamma (15.22,91.1)*PPI |  |
| Hospital costs year of transplant | 13,932 | Gamma (13.78,686.4)*PPI |  |
| **QALY Weights** |  |  |  |
| *Uninfected* |  |  |  |
| Ex-injector | 0.85 | Uniform (0.8,0.9) | Assumption |
| PWID | 0.73 | Uniform (0.68, 0.78) | ^8^ |
| Homeless Ex-injector | 0.76 | Uniform (0.7,0.81) | ^11^ |
| *Mild HCV* (F0 and F1) |  |  |  |
| Without Treatment | 0.77 | Beta (521.2375,155.6943) | ^9^ |
| SVR | 0.82 | Beta (65.8678,14.4588) |  |
| *Moderate HCV (F2 and F3)* |  |  |  |
| Without Treatment | 0.66 | Beta (168.2461, 86.6723) |  |
| SVR | 0.72 | Beta (58.0608,22.592) |  |
| *Compensated Cirrhosis* |  |  |  |
| Without Treatment | 0.55 | Beta (47.1021, 38.5381) |  |
| SVR | 0.61 | Beta (58.0608,37.1124) |  |
| Decompensated cirrhosis | 0.45 | Beta (123.75, 151.25) |  |
| Hepatocellular Carcinoma | 0.45 | Beta (123.75, 151.25) |  |
| Liver transplant | 0.45 | Beta (123.75, 151.25) |  |
| Post-transplant | 0.67 | Beta (59.2548, 29.1852) |  |

PPI = 1.5083 from NHS pay and prices index from 2003 to 2018 and from NHS Cost inflation index pay and prices for 2018 onwards

## Supplementary Modelling Results

### Supplementary Calibration figures

**Supplementary Figure 2:** Improvement in different error measures incorporated in the approximate Bayesian calibration routine. Error measures are on the log scale because each have a different magnitude.

**
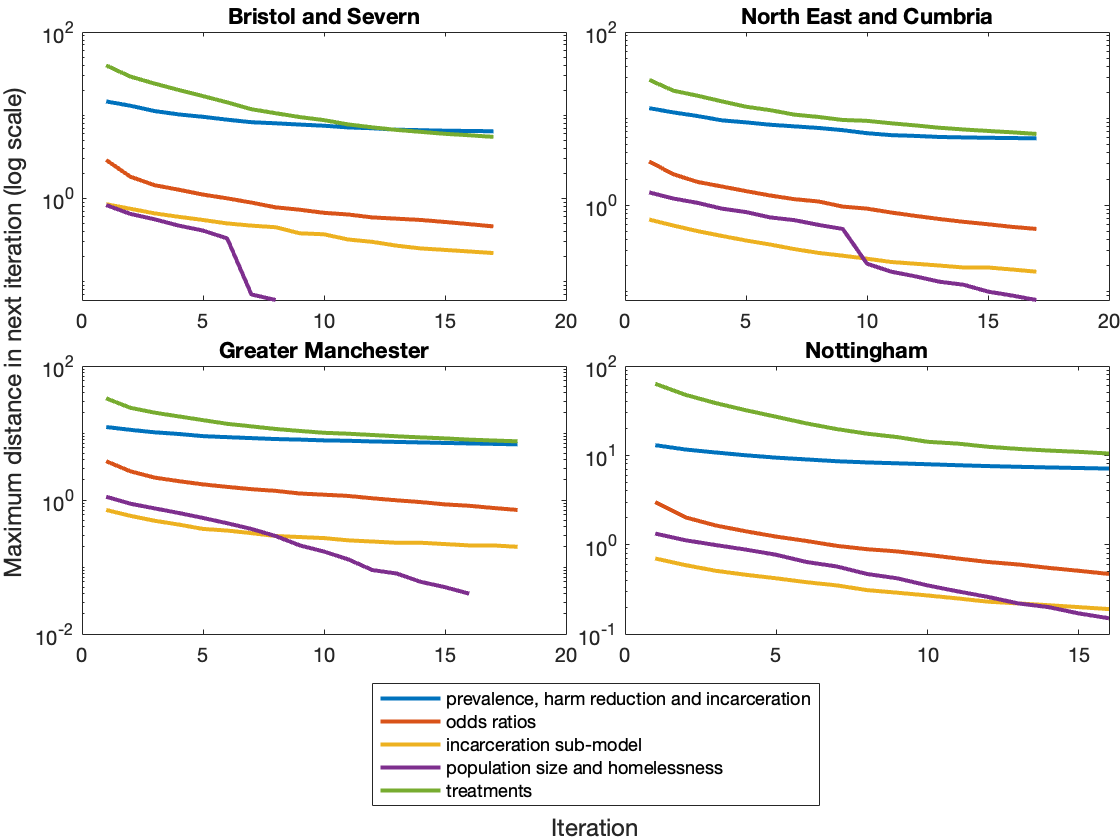
**

**Supplementary Figure 3:** Prior and posterior distributions for parameters that have non-precise priors.

Bristol


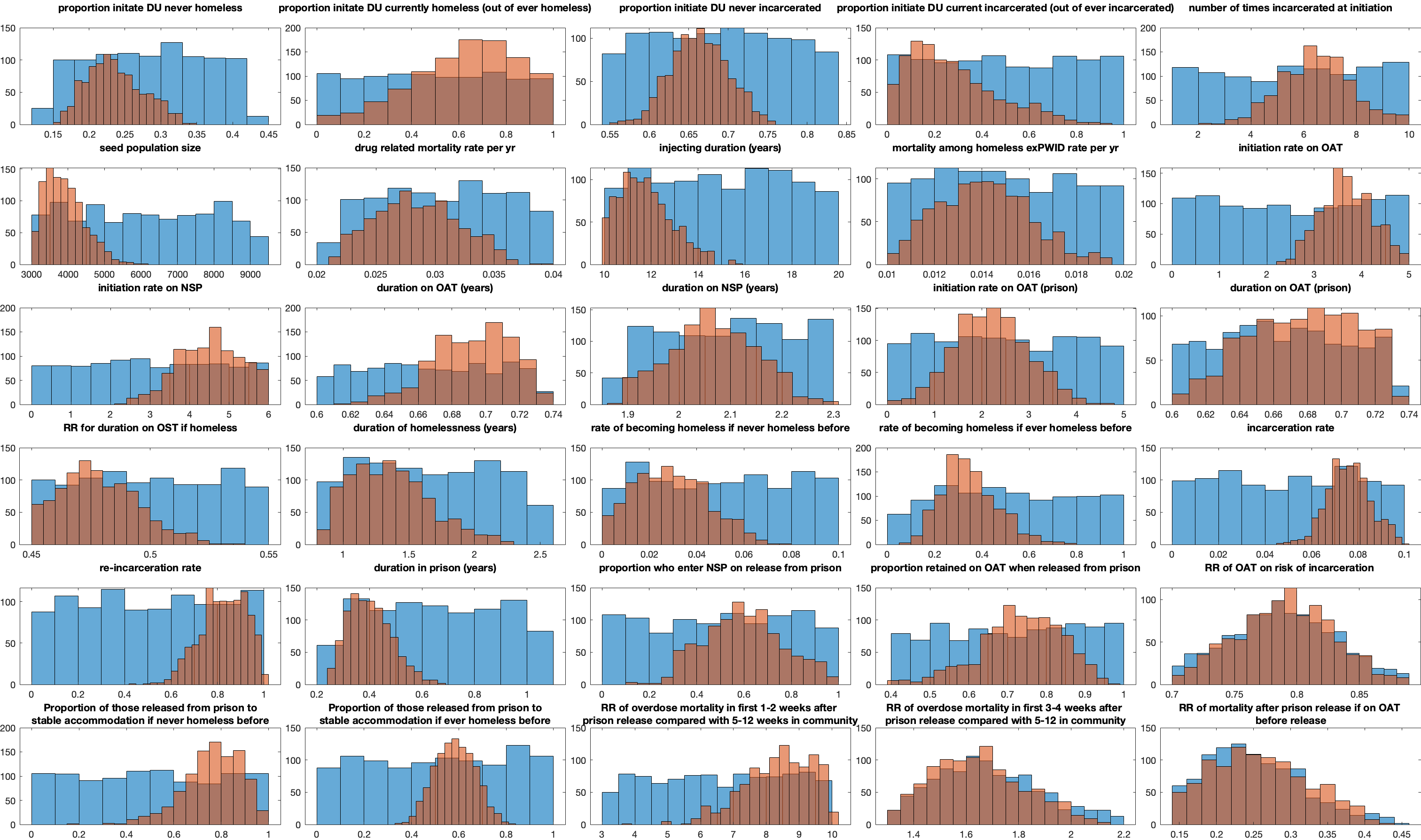


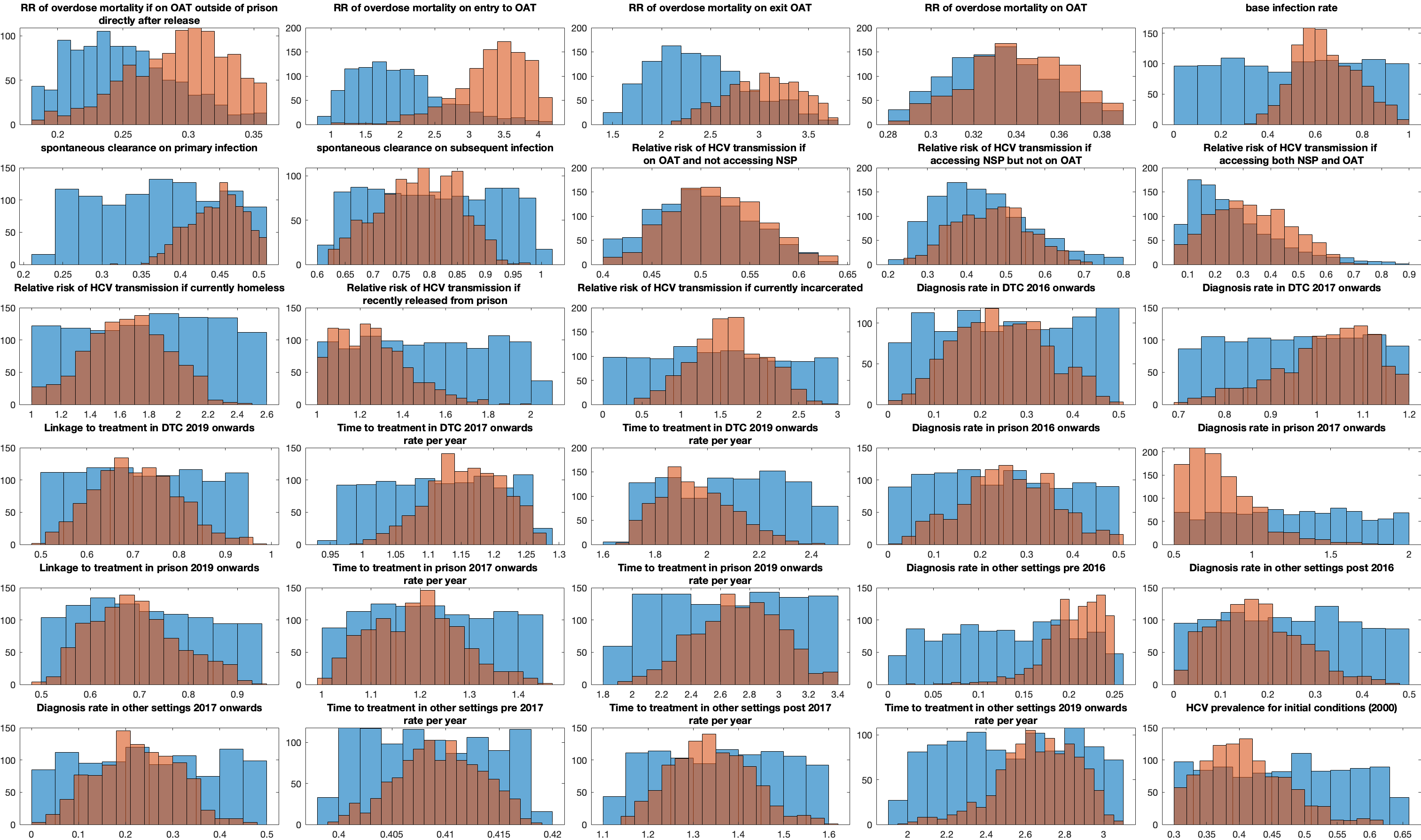


Northeast and Cumbria


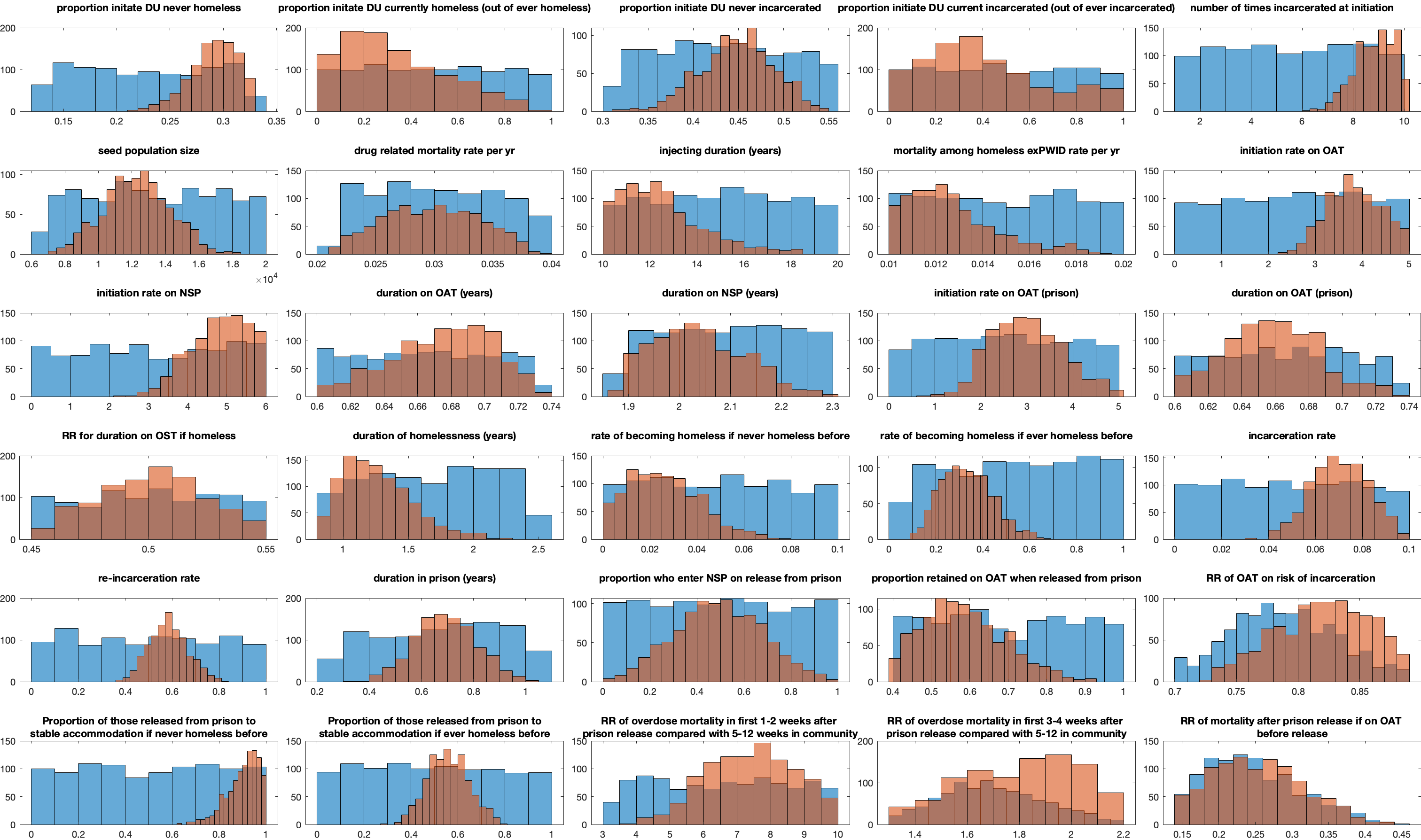


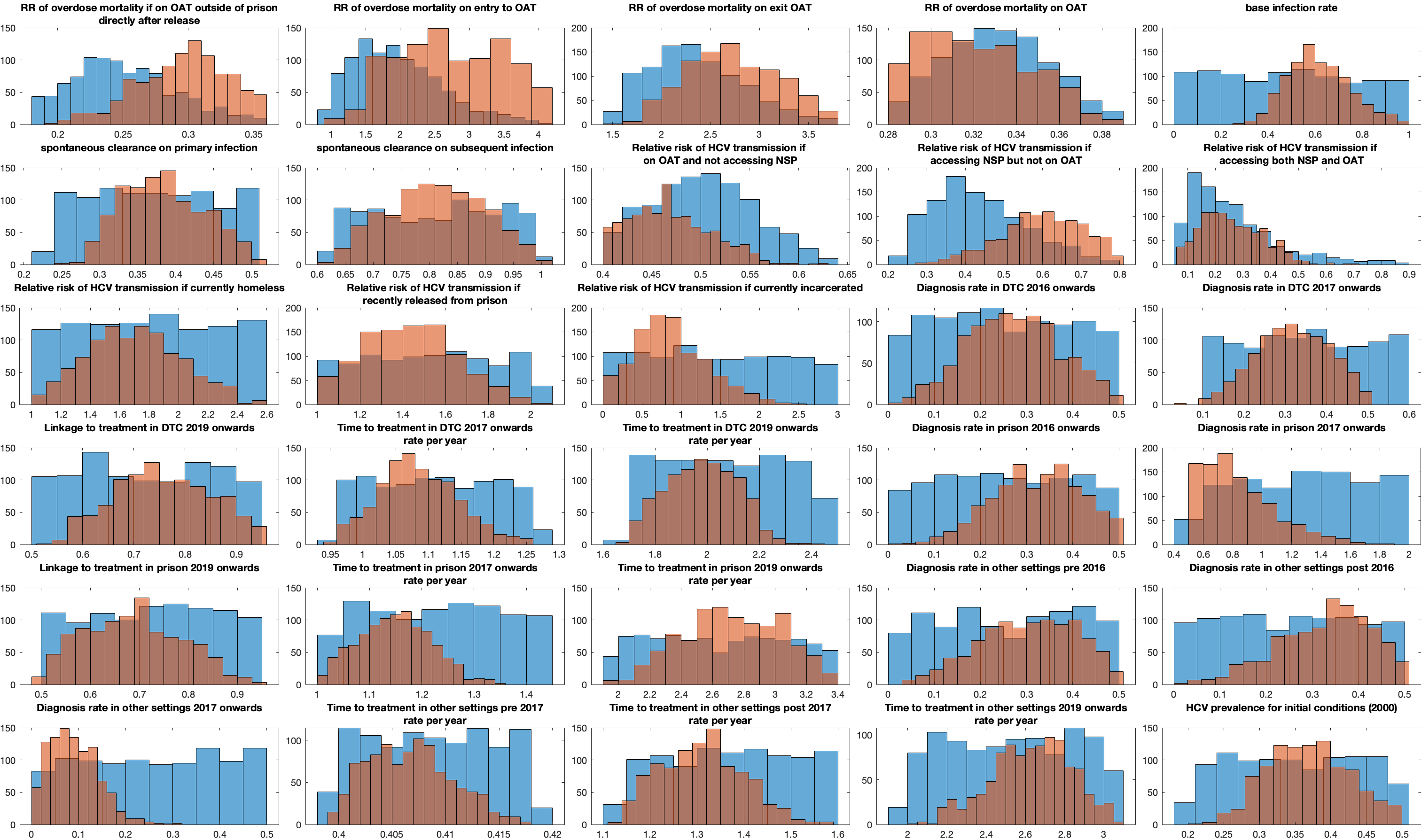


Greater Manchester


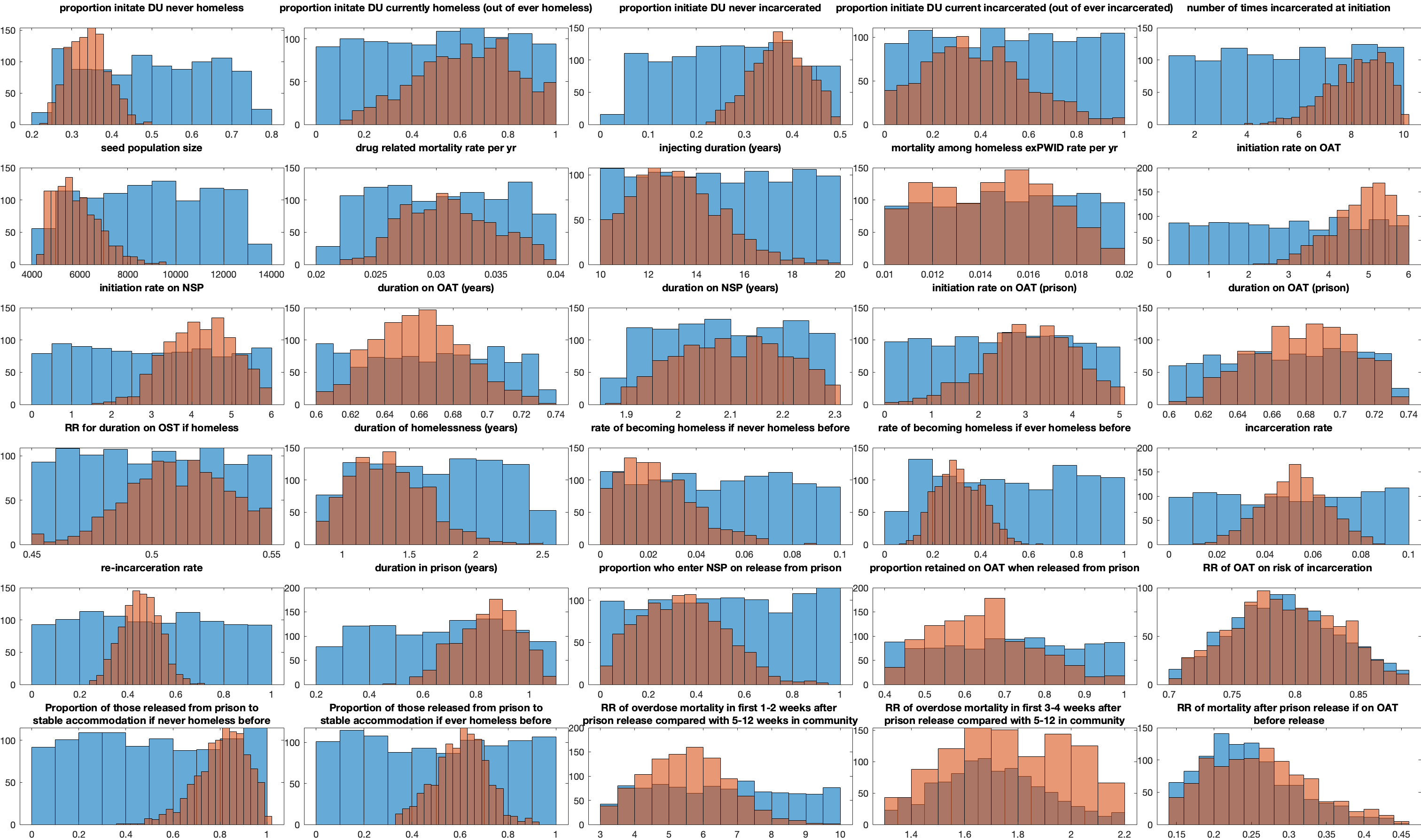


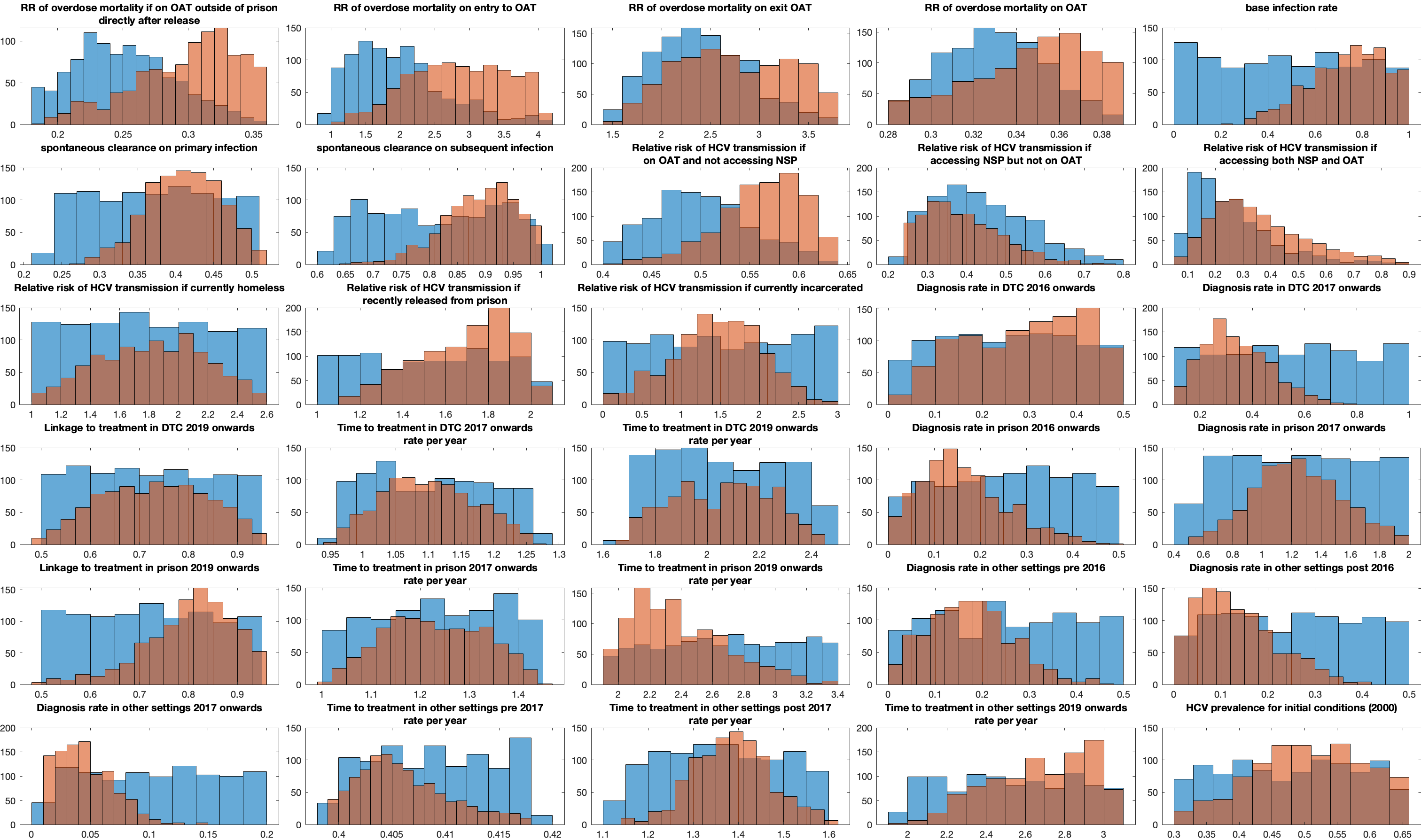


Nottingham


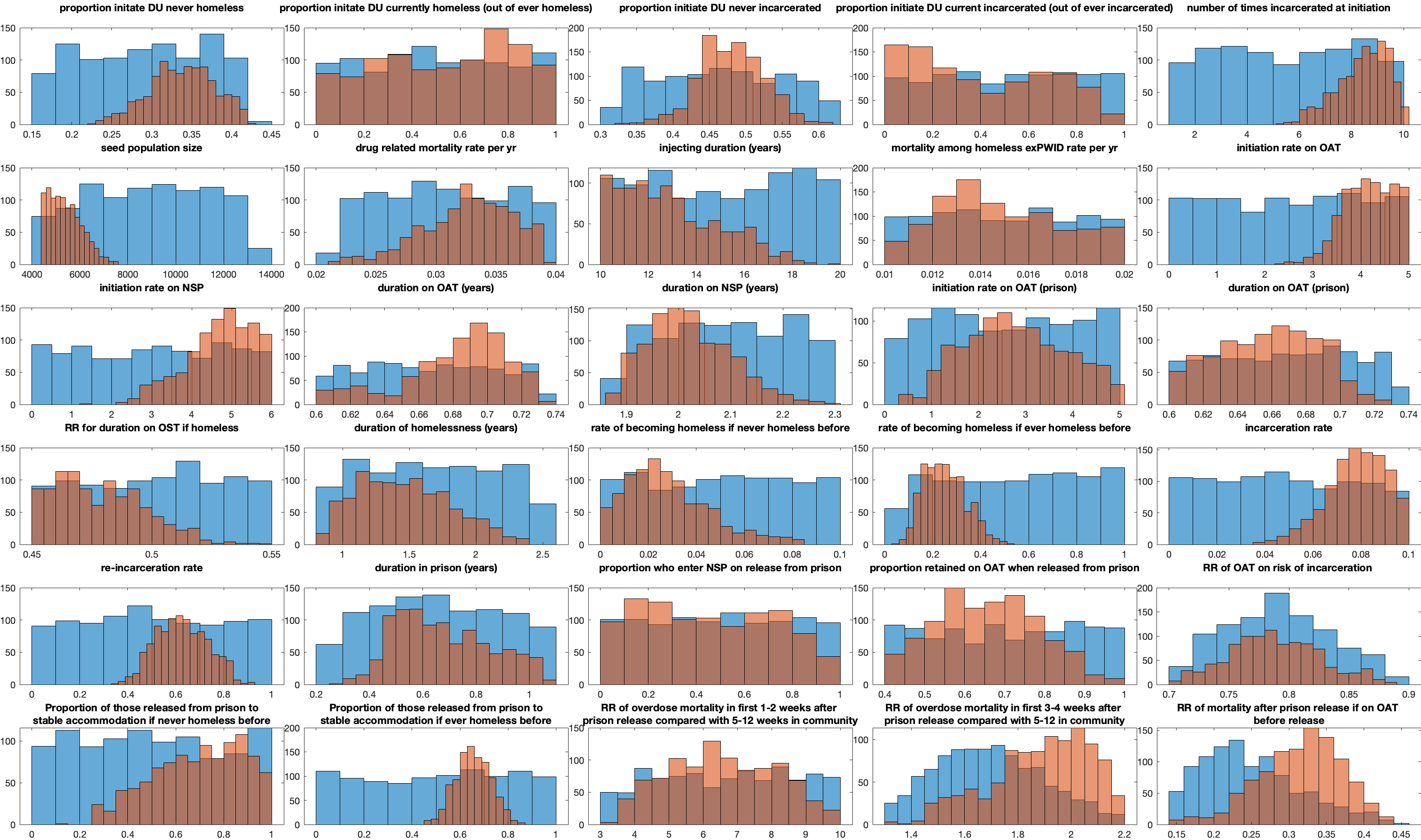


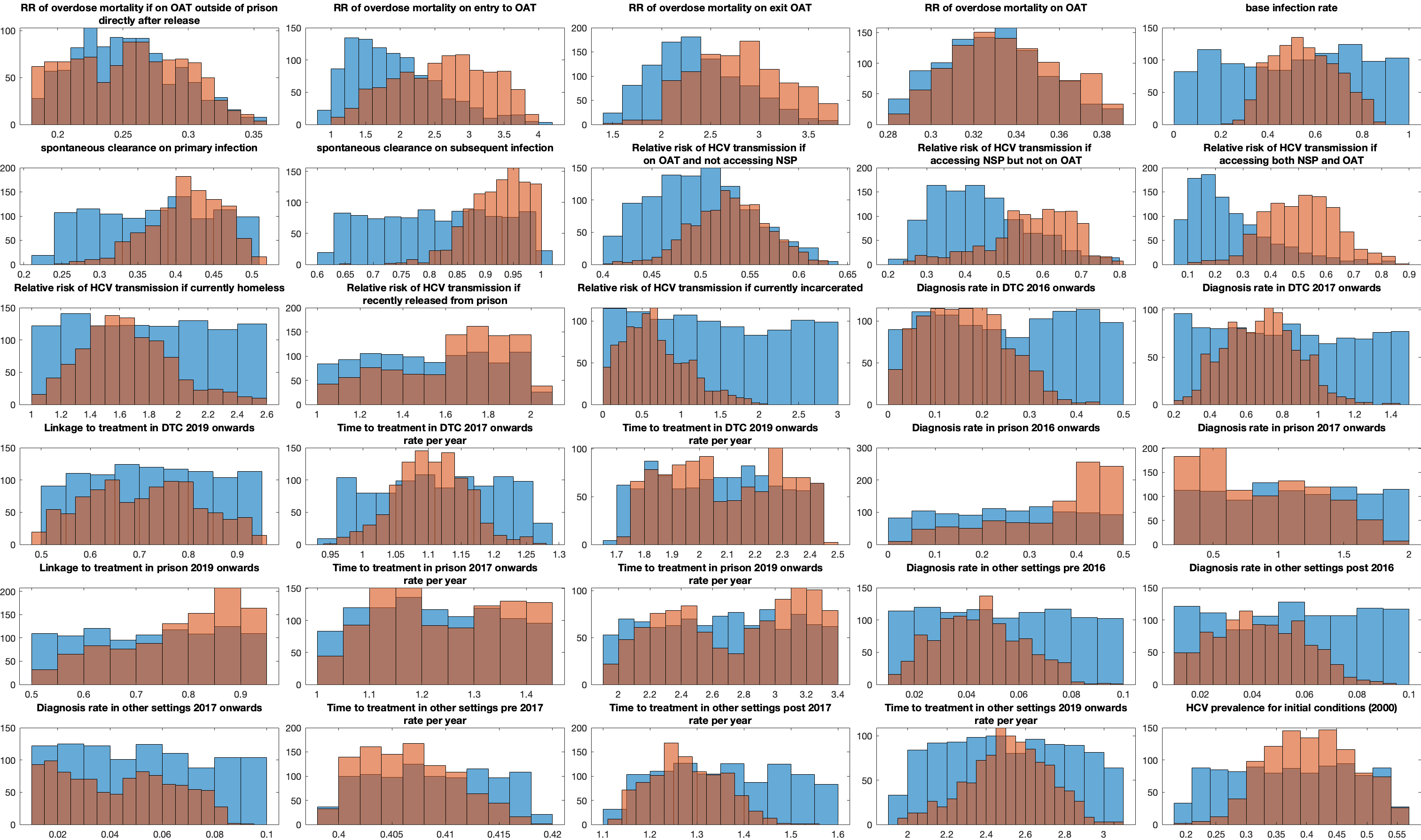


**Supplementary figure 4**: A comparison of the model projections with different data estimates used in the model calibration

Bristol


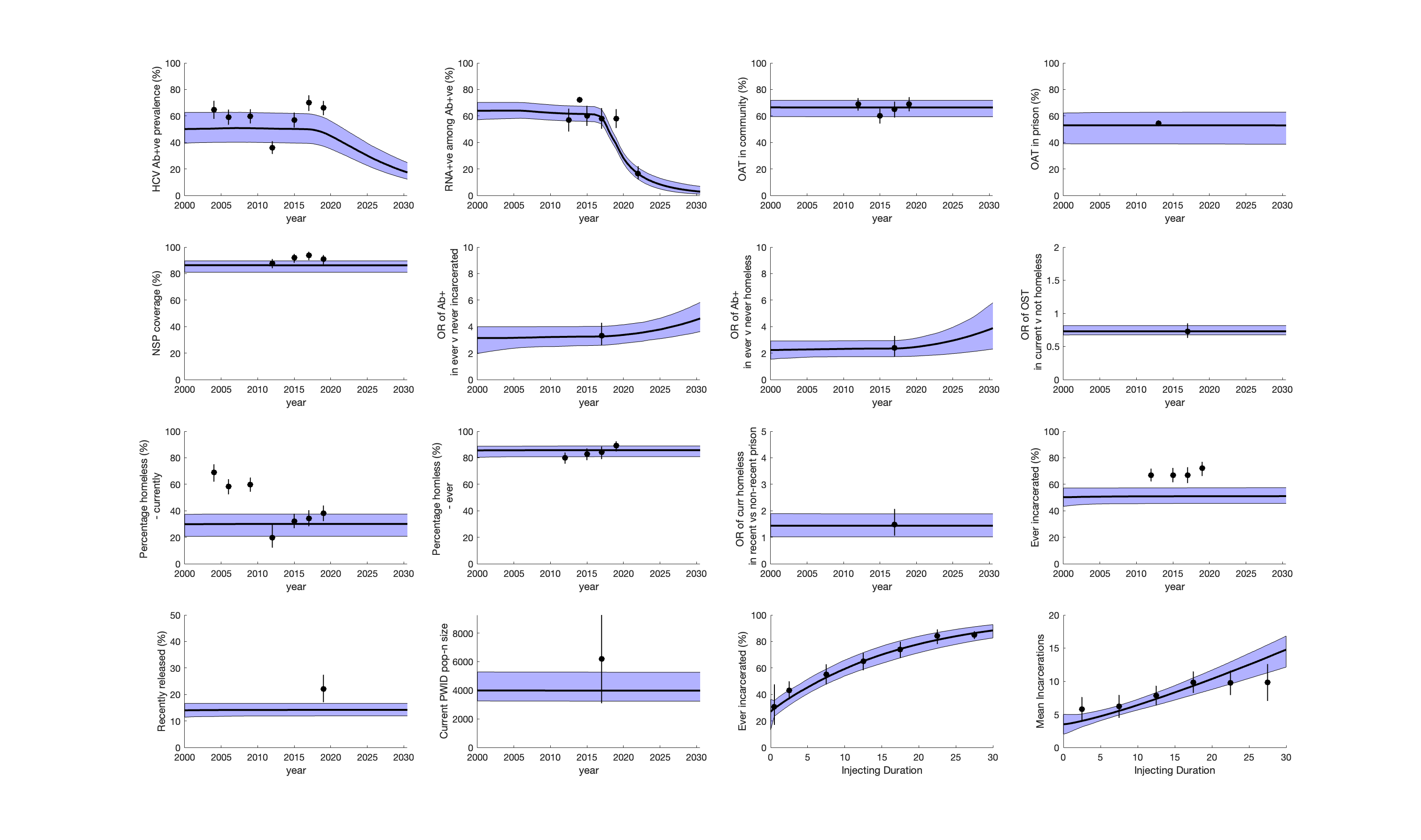


Northeast and Cumbria


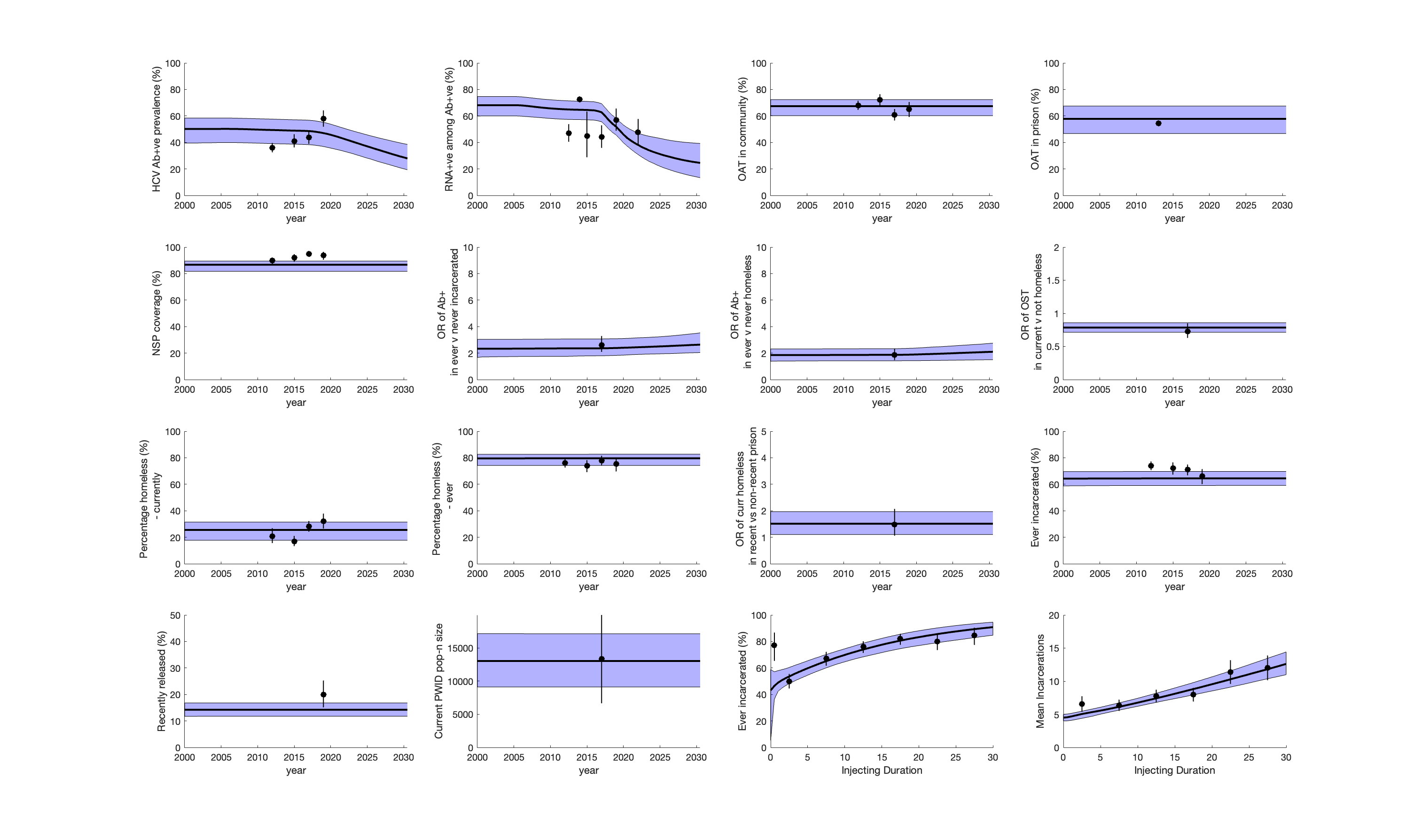


Greater Manchester


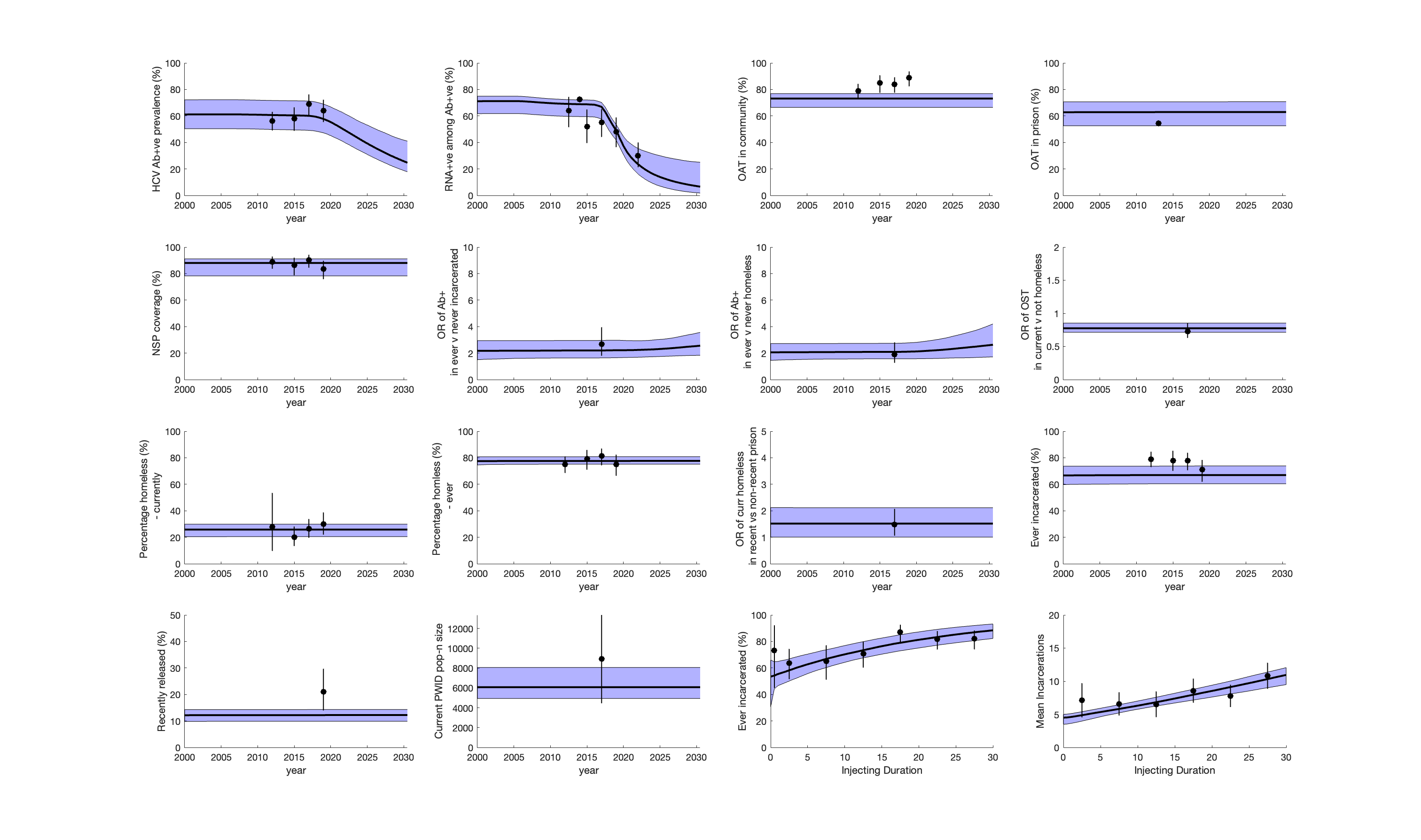


Nottingham


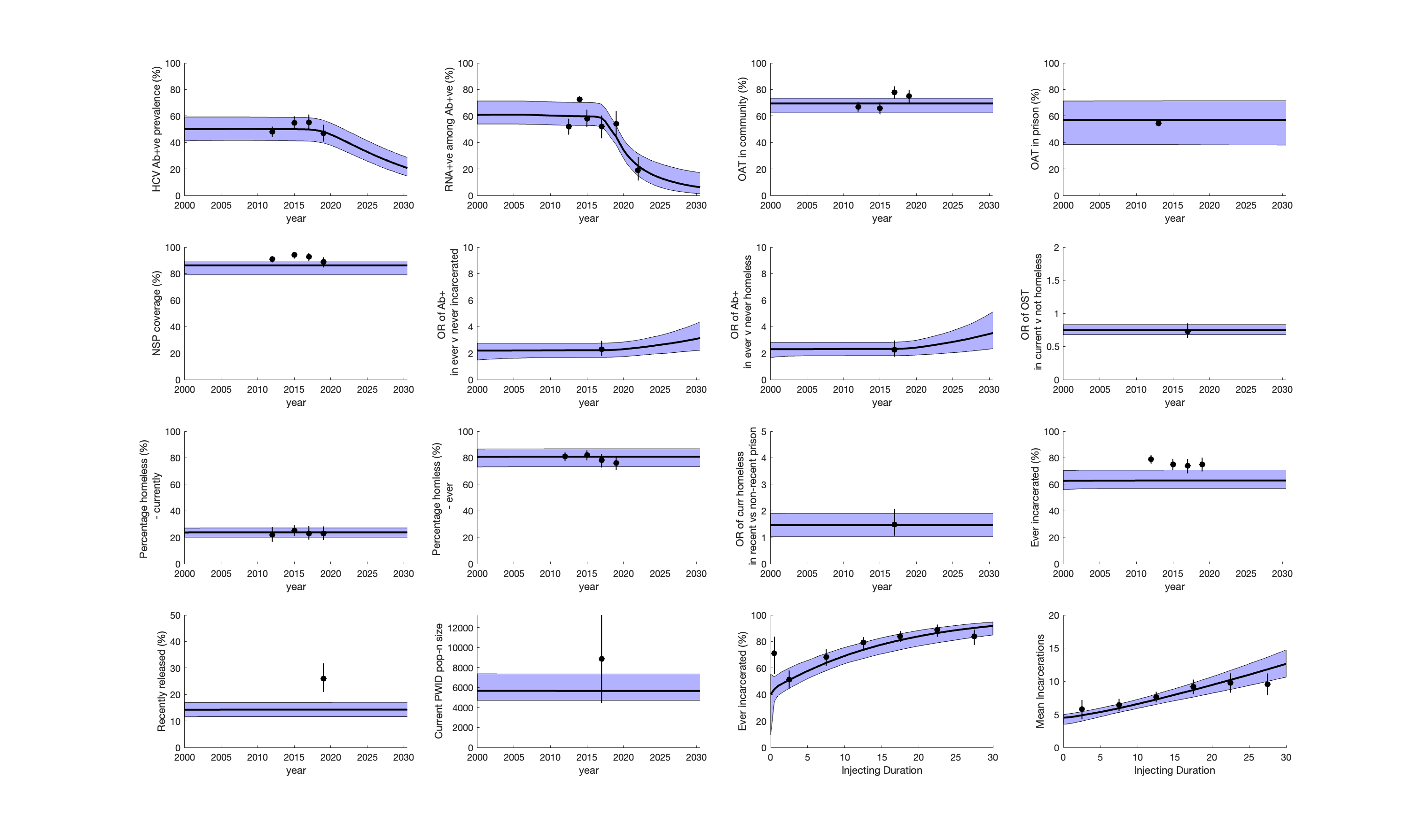


**Supplementary Figure 5**: Comparison of model projections with cumulative treatment numbers for different testing settings in each ODN region

Bristol and Severn North East and Cumbria


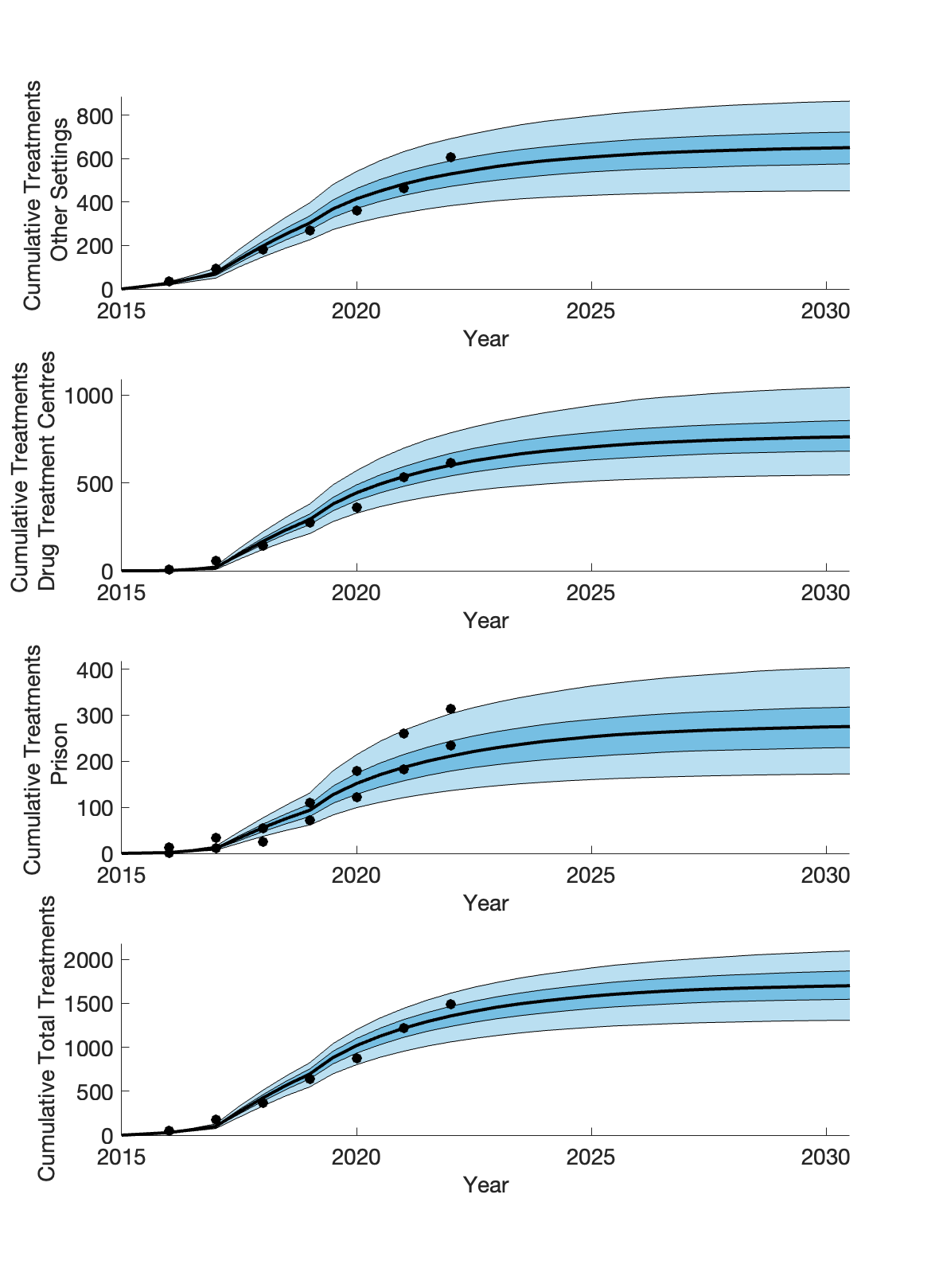

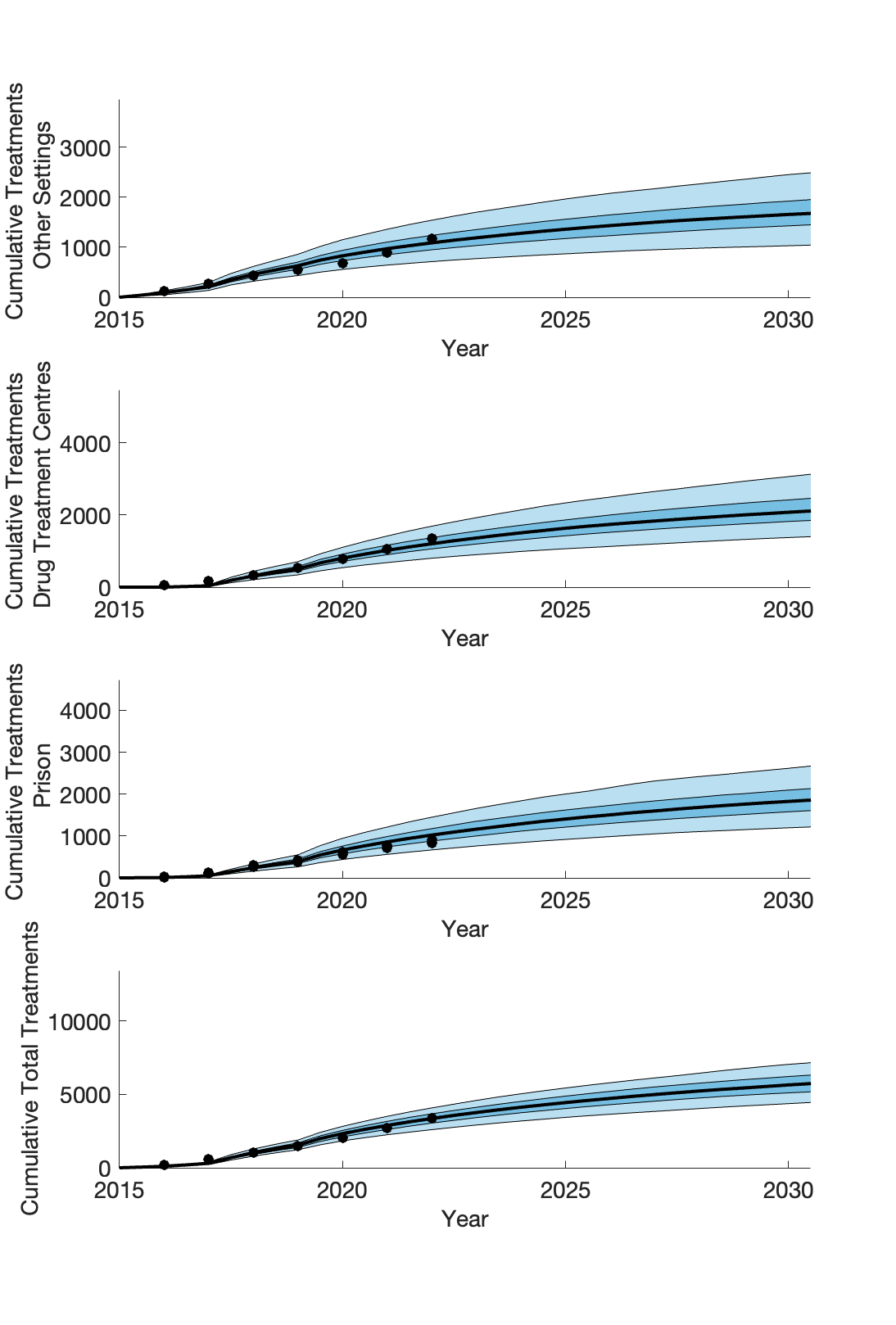


Nottingham Greater Manchester


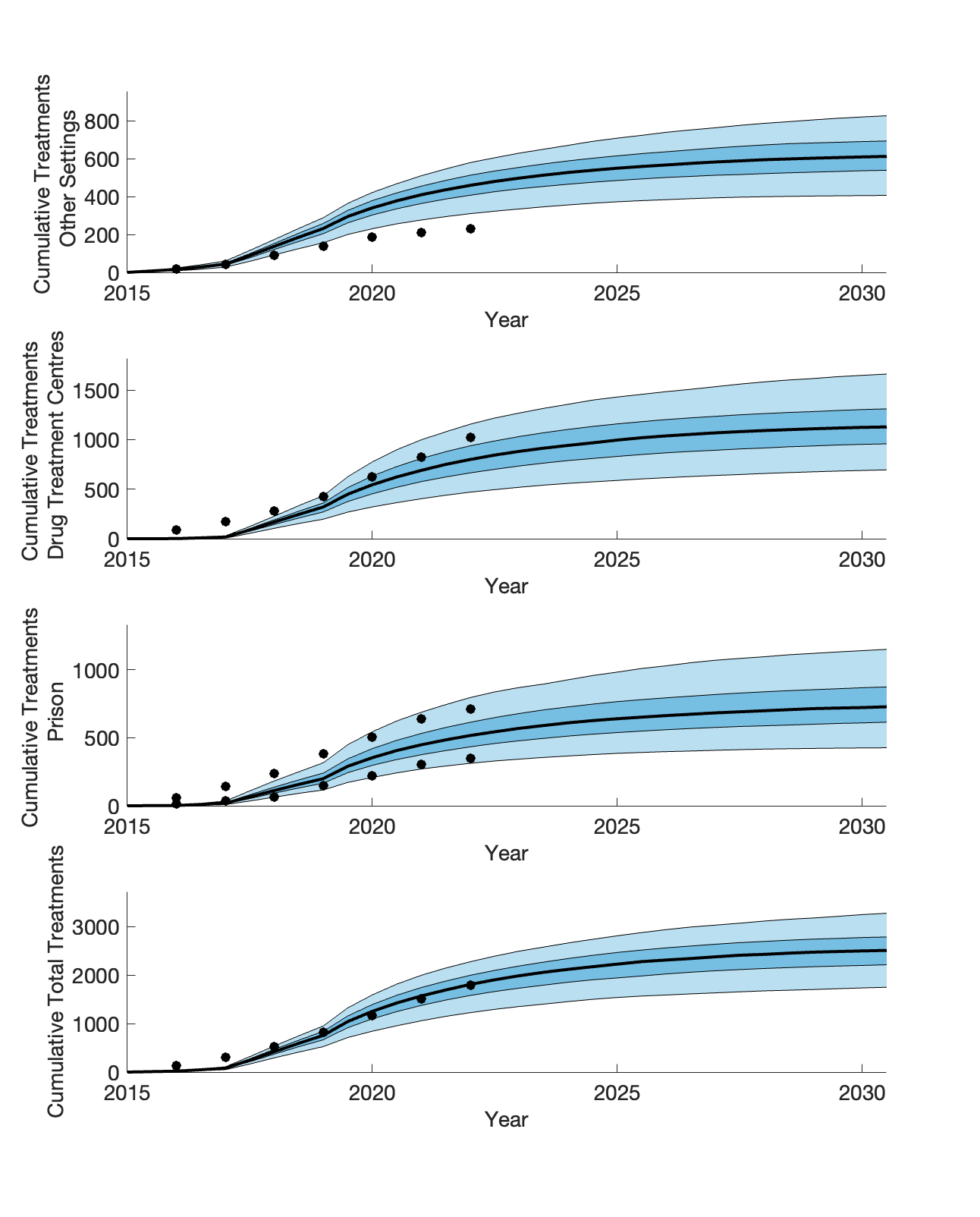

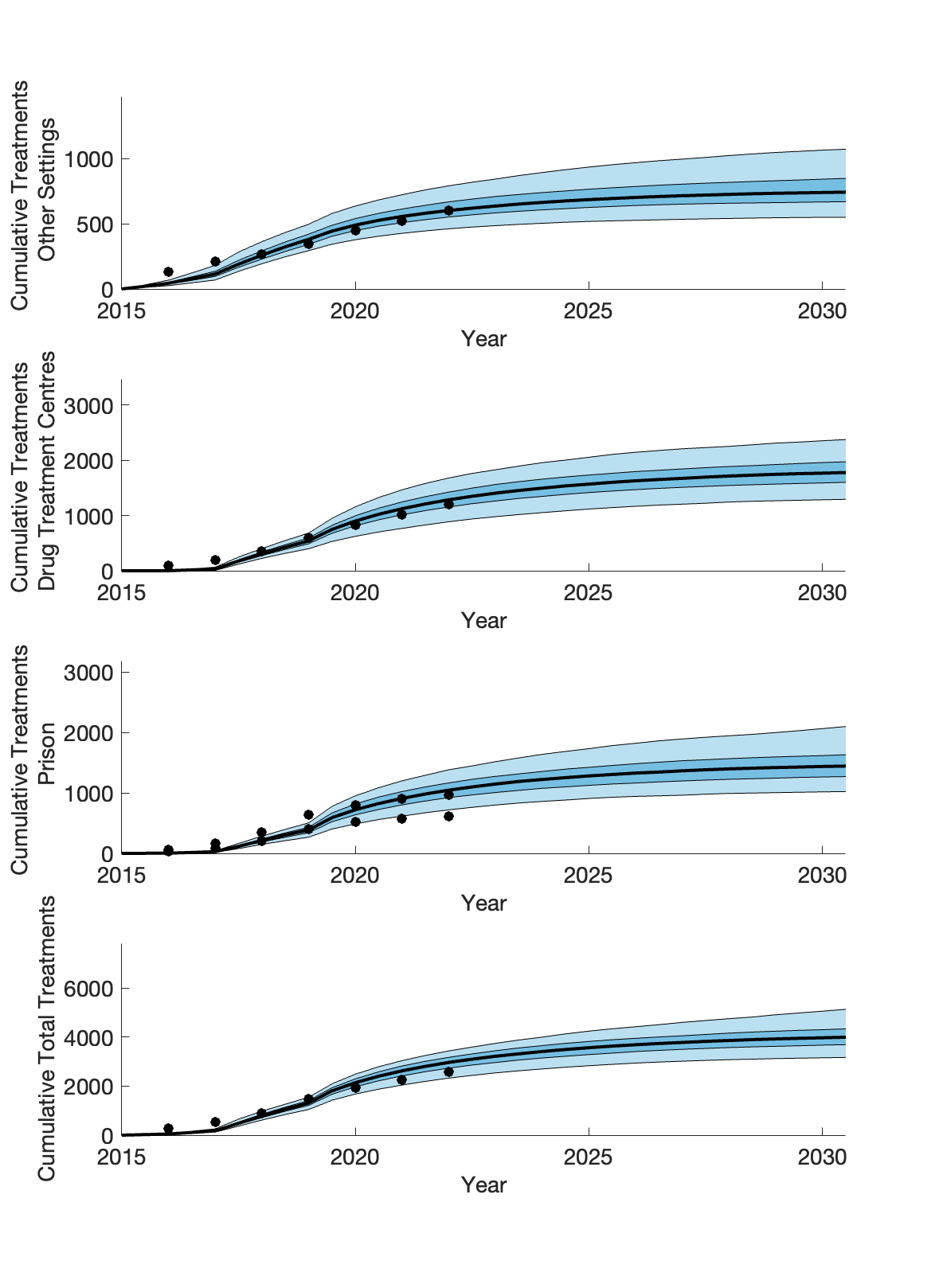


**Supplementary Figure 6**: Uncertainty analysis across the model fits for all four regions showing the association between the model projected RNA prevalence among PWID testing antibody positive in 2023 and the probability that incidence will be less than 2 per 100 person years in 2030 assuming testing and treatment pathway remains as it currently is.

### Ancova Analysis

**Supplementary Table 20**: ANCOVA results for impact of baseline scenario on HIV incidence in 2030. Table gives the percentage of sum of squares contributed by different parameters, shown in magnitude order for Bristol and Severn. Table only shows those parameters that contribute more than 3% to the overall sum of squares.

|  | **Bristol and Severn** | **Northeast and Cumbria** | **Greater Manchester** | **Nottingham** |
| --- | --- | --- | --- | --- |
| Transmission rate | 25.9 | 20.2 | 16.6 | 24.1 |
| RR of transmission when in prison | 13.1 | 8.4 | 3.5 | 5.2 |
| RR of transmission when on OAT and NSP | 12.4 | 12.8 | 10.7 | 19.3 |
| Linkage to treatment from 2019 in DTC | 7.2 |  | 3.4 | 3.9 |
| Drug treatment diagnosis rate 2017 | 5.2 | 9.4 | 37.8 | 18.5 |
| Diagnosis rate in other settings from 2017 | 5.2 | 4.4 |  |  |
| Initiation rate onto OAT | 5 | 3.6 |  |  |
| RR of transmission when homeless | 4 | 7.7 | 4.6 | 4.8 |
| Spontaneous clearance rate from primary infection | 3.8 | 5.3 | 4.7 | 6 |
| RR of transmission when on NSP | 3.5 | 5.6 |  | 3.7 |
| Diagnosis rate in prison from 2017 |  | 7.1 | 6.6 |  |

### Cost breakdown

**Supplementary Table 21**: Breakdown of mean costs by setting and activity

|  | **Counterfactual** | **Status quo** | **Incremental Costs** |
| --- | --- | --- | --- |
| **Bristol and Severn** |  |  |  |
| HCV Care | £63,997,408 | £51,970,855 | -£12,026,553 |
| Testing DTC | £74,535 | £4,957,751 | £4,883,216 |
| Testing Prison | £78,306 | £774,411 | £696,105 |
| Testing Other | £975,565 | £1,429,923 | £454,359 |
| Treatment DTC | £185,014 | £7,058,338 | £6,873,324 |
| Treatment Prison | £1,607,625 | £3,679,496 | £2,071,871 |
| Treatment Other | £20,637,979 | £24,249,044 | £3,611,064 |
| SVR DTC | £9,411 | £136,071 | £126,660 |
| SVR Prison | £7,782 | £210,631 | £202,849 |
| SVR Other | £42,292 | £393,721 | £351,429 |
| Total | £87,615,918 | £94,860,242 | £7,244,324 |
| **Northeast and Cumbria** | |  |  |
| HCV Care | £218,913,149 | £198,184,746 | -£20,728,402 |
| Testing DTC | £231,403 | £4,942,068 | £4,710,666 |
| Testing Prison | £286,724 | £1,308,780 | £1,022,056 |
| Testing Other | £7,463,933 | £3,243,846 | -£4,220,087 |
| Treatment DTC | £560,001 | £21,880,878 | £21,320,878 |
| Treatment Prison | £3,655,407 | £20,099,322 | £16,443,915 |
| Treatment Other | £40,587,522 | £30,566,680 | -£10,020,842 |
| SVR DTC | £25,984 | £349,917 | £323,933 |
| SVR Prison | £27,057 | £1,257,434 | £1,230,377 |
| SVR Other | £65,556 | £556,355 | £490,800 |
| Total | £271,816,735 | £282,390,028 | £10,573,292 |
| **Greater Manchester** |  |  |  |
| HCV Care | £131,179,372 | £112,296,753 | -£18,882,618 |
| Testing DTC | £65,630 | £3,278,001 | £3,212,371 |
| Testing Prison | £84,452 | £1,414,841 | £1,330,389 |
| Testing Other | £1,092,330 | £1,963,715 | £871,385 |
| Treatment DTC | £245,548 | £16,946,098 | £16,700,551 |
| Treatment Prison | £3,033,633 | £15,676,908 | £12,643,274 |
| Treatment Other | £51,368,454 | £38,044,535 | -£13,323,920 |
| SVR DTC | £15,603 | £241,223 | £225,620 |
| SVR Prison | £11,945 | £1,885,062 | £1,873,117 |
| SVR Other | £102,060 | £714,856 | £612,795 |
| Total | £187,199,028 | £192,461,993 | £5,262,965 |
| **Nottingham** |  |  |  |
| HCV Care | £74,069,510 | £60,039,976 | -£14,029,533 |
| Testing DTC | £66,475 | £4,667,949 | £4,601,474 |
| Testing Prison | £409,283 | £1,551,133 | £1,141,849 |
| Testing Other | £344,729 | £3,131,159 | £2,786,431 |
| Treatment DTC | £284,232 | £10,971,065 | £10,686,832 |
| Treatment Prison | £4,197,038 | £10,414,831 | £6,217,793 |
| Treatment Other | £15,523,498 | £25,038,723 | £9,515,225 |
| SVR DTC | £4,781 | £160,547 | £155,766 |
| SVR Prison | £8,060 | £1,112,651 | £1,104,591 |
| SVR Other | £28,654 | £428,796 | £400,143 |
| Total | £94,936,261 | £117,516,832 | £22,580,571 |

### Cost Effectiveness Sensitivity analysis

**Supplementary Table 22** Cost-effectiveness sensitivity analysis. Bracketed numbers are negative.

|  | **Counterfactual cost** | **Baseline cost** | **Incremental cost** | **Counterfactual QALY** | **Baseline QALY** | **Incremental QALY** | **Mean ICER** | **% runs CE** | **% runs cost-saving** |
| --- | --- | --- | --- | --- | --- | --- | --- | --- | --- |
| **Bristol and Severn** | |  |  |  |  |  |  |  |  |
| Baseline then counterfactual from 2031 | 87,615,918 | 91,469,502 | 3,853,584 | 261,973 | 271,815 | 9,842 | 392 | 100% | 25% |
| Minimum utility | 87,615,918 | 94,860,242 | 7,244,324 | 278,258 | 285,181 | 6,923 | 1,046 | 100% | 16% |
| Discount rate zero | 181,341,472 | 158,658,598 | (22,682,873) | 570,174 | 600,444 | 30,270 | (749) | 100% | 80% |
| Discount rate 5% | 68,658,949 | 80,431,427 | 11,772,478 | 201,310 | 208,420 | 7,110 | 1,656 | 100% | 7% |
| DAA costs £3000 | 72,202,425 | 71,532,622 | (669,803) | 261,973 | 272,574 | 10,601 | (63) | 100% | 39% |
| Time horizon 25years | 63,572,499 | 80,671,603 | 17,099,104 | 180,404 | 185,223 | 4,819 | 3,549 | 100% | 2% |
| **Northeast and Cumbria** | |  |  |  |  |  |  |  |  |
| Baseline then counterfactual from 2031 | 271,816,735 | 281,280,086 | 9,463,350 | 852,203 | 866,520 | 14,317 | 661 | 100% | 25% |
| Minimum utility | 271,816,735 | 282,390,028 | 10,573,292 | 896,755 | 907,185 | 10,431 | 1,014 | 100% | 29% |
| Discount rate zero | 562,862,808 | 510,427,044 | (52,435,764) | 1,831,718 | 1,889,094 | 57,376 | (914) | 100% | 84% |
| Discount rate 5% | 212,725,995 | 231,876,345 | 19,150,349 | 658,138 | 670,224 | 12,086 | 1,584 | 100% | 7% |
| DAA costs £3000 | 241,023,826 | 233,842,230 | (7,181,596) | 852,203 | 870,867 | 18,664 | (385) | 100% | 59% |
| Time horizon 25years | 197,103,765 | 229,313,678 | 32,209,913 | 594,746 | 601,844 | 7,098 | 4,538 | 100% | 0% |
| **Greater Manchester** | |  |  |  |  |  |  |  |  |
| Baseline then counterfactual from 2031 | 187,199,028 | 193,152,569 | 5,953,541 | 391,044 | 405,375 | 14,331 | 415 | 100% | 20% |
| Minimum utility | 187,199,028 | 192,461,993 | 5,262,965 | 418,802 | 428,373 | 9,571 | 550 | 100% | 27% |
| Discount rate zero | 384,617,446 | 322,127,675 | (62,489,771) | 846,737 | 895,531 | 48,794 | (1,281) | 100% | 100% |
| Discount rate 5% | 147,274,796 | 163,223,020 | 15,948,224 | 301,131 | 312,588 | 11,457 | 1,392 | 100% | 5% |
| DAA costs £3000 | 149,654,147 | 145,246,366 | (4,407,781) | 391,044 | 408,115 | 17,071 | (258) | 100% | 45% |
| Time horizon 25years | 136,754,496 | 163,274,415 | 26,519,919 | 270,751 | 278,510 | 7,758 | 3,418 | 100% | 1% |
| **Nottingham** |  |  |  |  |  |  |  |  |  |
| Baseline then counterfactual from 2031 | 94,936,261 | 111,404,734 | 16,468,473 | 351,173 | 363,415 | 12,242 | 1,345 | 100% | 8% |
| Minimum utility | 94,936,261 | 117,516,832 | 22,580,571 | 370,906 | 380,312 | 9,405 | 2,401 | 100% | 5% |
| Discount rate zero | 198,156,107 | 197,569,868 | (586,239) | 756,420 | 797,985 | 41,565 | (14) | 100% | 42% |
| Discount rate 5% | 74,166,649 | 99,267,855 | 25,101,206 | 270,960 | 280,201 | 9,241 | 2,716 | 100% | 3% |
| DAA costs £3000 | 81,169,197 | 86,502,326 | 5,333,129 | 351,173 | 365,204 | 14,031 | 380 | 100% | 22% |
| Time horizon 25years | 68,320,499 | 100,009,044 | 31,688,545 | 244,529 | 250,375 | 5,846 | 5,421 | 100% | 0% |

### Comparison to other ODN regions

**Supplementary Table 23**: Comparison of the posterior ranges for key parameters in our four ODN regions and point estimates for these parameters in other ODN regions. Blue shading denotes >10% deviation from the combined posterior range for our four ODN and red shading denotes <10% deviation from this range. See notes after table.

| ODN regions | Population size PWID 2017** | Treatment number PWID**& | % of baseline infections treated by end 2022 | Ab prevalence before scale-up (2011-2015) | RNA positivity among AB+ before scale-up (2011-2015) | % ever incarcerated | % OAT coverage |
| --- | --- | --- | --- | --- | --- | --- | --- |
| Northeast and Cumbria^ | 13025  (9122-17136) | 3758  (2905-4583) | 92%  (76.2-108.1%) | 48.9%  (38.7-57.1) | 64.5%  (57.1-71.0) | 64.4%  (59.0-69.7) | 67.4%  (60.2-72.4) |
| Nottingham^ | 5658  (4715-7358) | 1987  (1352-2495) | 112.9%  (96.8- 124.7%) | 50.0%  (41.2-58.8) | 59.7%  (52.8-70.1) | 62.8%  (56.8-70.7) | 69.4%  (62.3-73.4) |
| Greater Manchester^ | 6068  (4936-8058) | 3218  (2540-3744) | 124.8%  (108.3-135.0%) | 60.4%  (49.4- 71.4) | 68.8%  (59.4-72.1) | 66.9%  (60.4-73.8) | 73.2%  (66.5-76.8) |
| Bristol and Severn^ | 3975  (3239-5272) | 1457  (1134-1738) | 113.1%  (106.5-120.1%) | 50.1%  (39.5-61.9) | 61.4%  (55.9-67.3) | 50.9%  (45.5-57.3) | 66.4%  (59.4-71.8) |
|  |  |  |  |  |  |  |  |
| Eastern Hepatitis Network | 8720 | 1642 | 110% | 44.8% (654) | 54.4% (235) | 68.6% (1276) | 78.7% (1299) |
| West London | 3390 | 695 | 51% | 59.3% (266) | 68.1% (135) | 64.2% (417) | 79.0% (428) |
| North Central London | 6250 | 548 | 22% | 63.6% (231) | 63.7% (102) | 69.8% (596) | 84.0% (606) |
| Barts | 2670 | 590 | 55% | 59.5% (497) | 67.6% (259) | 71.8% (592) | 78.9% (620) |
| South Thames Hepatitis Network | 4660 | 1519 | 111% | 61.5% (239) | 47.6% (124) | 72.8% (644) | 81.4% (656) |
| Surrey Hepatitis Services* | 2590 | 716 |  | 54.5% (22) | 58.3% (12) | 70.3% (37) | 86.4% (37) |
| Sussex Hepatology Network | 3570 | 967 | 69% | 65.7% (595) | 59.5% (376) | 65.8% (798) | 77.4% (809) |
| Oxford University Hospitals Trust | 2370 | 1105 | 195% | 43.3% (340) | 55.2% (132) | 61.2% (589) | 73.7% (598) |
| Wessex Hep C ODN | 7490 | 1371 | 69% | 53.0% (283) | 50.4% (137) | 66.0% (1049) | 72.7% (1079) |
| Southwest Peninsula | 5390 | 869 | 103% | 40.2% (291) | 38.9% (108) | 60.0% (384) | 72.3% (390) |
| Kent Network via Kings | 4960 | 808 | 38% | 57.9% (126) | 74.1% (58) | 72.5% (251) | 80.0% (255) |
| Cheshire and Merseyside | 4910 | 1879 | 95% | 72.9% (422) | 55.4% (285) | 73.1% (655) | 68.1% (683) |
| South Yorkshire | 5800 | 1890 | 107% | 54.0% (252) | 56.2% (128) | 69.4% (504) | 68.4% (526) |
| Humberside and North Yorkshire | 6890 | 1132 | 46% | 50.0% (94) | 70.7% (41) | 74.3% (285) | 81.0% (295) |
| West Yorkshire | 6840 | 2365 | 96% | 57.1 (854) | 63.3% (433) | 75.3% (1225) | 74.7% (1268) |
| Lancashire and South Cumbria | 8470 | 1053 | 29% | 62.0% (326) | 70.2% (188) | 73.2% (610) | 62.0% (640) |
| Leicester* | 3060 | 495 |  | 49.4% (87) | 61.9% (42) | 71.3% (122) | 73.0% (122) |
| Birmingham | 14570 | 3901 | 112% | 38.4% (962) | 62.0% (345) | 67.2% (1785) | 73.7% (1793) |

^posterior values from model fits; *2016-19 data; **not compared across ODN regions but used to calculate the proportion of baseline chronic infected PWID that have been treated; & Data estimates include all treatments in DTC and prison and among PWID in other settings, whereas for our modelled ODNs it is posterior range which has more uncertainty than data range due to assuming uncertainty in number resulting from under-reporting in treatment database, imputation and possible mis-reporting of injecting drug use status

**Notes**: Supplementary Table 23 compares our ODN regions to other regions in England in terms of the antibody and RNA prevalence among PWID before HCV treatment scaled up (i.e. 2011-2015), the average coverage of OAT, the proportion ever incarcerated, and estimated proportion of the baseline number of chronically infected PWID that have been treated over 2016-2022 (used as a proxy for treatment coverage). The table includes the posterior estimates for these parameters for our four modelled ODNs and the data estimates for the other ODNs that we did not model. This comparison shows that the models for our four ODN broadly capture most of the variation in these parameters across the other ODN, with at most 2/18 ODN being >10% outside the overall posterior parameter range from our model calibration for the four ODN (highlighted blue in the table). The only exception is the estimated proportion of chronically infected PWID that have been treated, with the estimates from our four ODNs (overall range 76-135%) being significantly higher than 6 of 18 ODN, with these 6 ODN estimating that 22-55% of baseline chronically infected PWID have been treated over 2016-2022. However, this is likely to reflect an under-reporting of injecting drug use status in these ODN regions as suggested by the RNA prevalence among antibody positive PWID (2020-2021), which for 4 of these 6 ODN is within the range of the data estimates for our modelled ODN regions (18-43% in our regions, not shown in table). This suggests a similar impact of treatment on the HCV epidemics among PWID in these ODN regions and so a similar level of treatment among PWID. The main exception is Humberside and North Yorkshire which has a very low estimated treatment coverage (46%) and high RNA prevalence in 2020/2021 (50%).

## **References**

1. Martin NK, Hickman M, Miners A, Hutchinson SJ, Taylor A, Vickerman P. Cost-effectiveness of HCV case-finding for people who inject drugs via dried blood spot testing in specialist addiction services and prisons. *BMJ open.* 2013;3(8).

2. Stone J, Martin NK, Hickman M, et al. Modelling the impact of incarceration and prison-based hepatitis C virus (HCV) treatment on HCV transmission among people who inject drugs in Scotland. *Addiction.* Jul 2017;112(7):1302-1314.

3. Vickerman P, Grebely J, Dore GJ, et al. The More You Look, the More You Find: Effects of Hepatitis C Virus Testing Interval on Reinfection Incidence and Clearance and Implications for Future Vaccine Study Design. *J. Infect. Dis.* Mar 28 2012;205(9):1342-1350.

4. Grebely J, Prins M, Hellard M, et al. Hepatitis C virus clearance, reinfection, and persistence, with insights from studies of injecting drug users: towards a vaccine. *Lancet Inf. Dis.* May 2012;12(5):408-414.

5. Public Health England. Annual report from the sentinel surveillance study of blood borne virus testing in England: data for January to December 2017 *Health Protection Report.* 2018;12.

6. Simmons R, Ireland G, Irving W, et al. Establishing the cascade of care for hepatitis C in England-benchmarking to monitor impact of direct acting antivirals. *J Viral Hepat.* Dec 14 2018;25:482-490.

7. Ward Z, Campbell L, Surey J, et al. The cost-effectiveness of an HCV outreach intervention for at-risk populations in London, UK. *J Antimicrob Chemother.* Nov 1 2019;74(Suppl 5):v5-v16.

8. McDonald SA, Hutchinson SJ, Palmateer NE, et al. Decrease in health-related quality of life associated with awareness of hepatitis C virus infection among people who inject drugs in Scotland. *J Hepatol.* Mar 2013;58(3):460-466.

9. Shepherd J, Jones J, Hartwell D, Davidson P, Price A, Waugh N. Interferon alpha (pegylated and non-pegylated) and ribavirin for the treatment of mild chronic hepatitis C: a systematic review and economic evaluation. *Health Technol Assess.* Mar 2007;11(11):1-205, iii.

10. Wright M, Grieve R, Roberts J, Main J, Thomas HC, Investigators UKMHCT. Health benefits of antiviral therapy for mild chronic hepatitis C: randomised controlled trial and economic evaluation. *Health Technol Assess.* Jul 2006;10(21):1-113, iii.

11. Lewer D, Aldridge RW, Menezes D, et al. Health-related quality of life and prevalence of six chronic diseases in homeless and housed people: a cross-sectional study in London and Birmingham, England. *BMJ open.* Apr 24 2019;9(4):e025192.

12. Wittenberg E, Bray JW, Gebremariam A, Aden B, Nosyk B, Schackman BR. Joint Utility Estimators in Substance Use Disorders. *Value Health.* Mar 2017;20(3):458-465.

13. National Institute of Health and Care Excellence (NICE). *Guide to the methods of technology appraisal 2013 (PMG9) (*[*https://www.nice.org.uk/process/pmg9/resources/guide-to-the-methods-of-technology-appraisal-2013-pdf-2007975843781)*2013](https://www.nice.org.uk/process/pmg9/resources/guide-to-the-methods-of-technology-appraisal-2013-pdf-2007975843781)2013).

14. Roberts K, Macleod J, Metcalfe C, et al. Cost effectiveness of an intervention to increase uptake of hepatitis C virus testing and treatment (HepCATT): cluster randomised controlled trial in primary care. *BMJ.* Feb 26 2020;368:m322.

15. Ward Z, Reynolds R, Campbell L, et al. Cost-effectiveness of the HepCATT intervention in specialist drug clinics to improve case-finding and engagement with HCV treatment for people who inject drugs in England. *Addiction.* Aug 2020;115(8):1509-1521.

16. Buchanan R, Cooper K, Grellier L, Khakoo SI, Parkes J. The testing of people with any risk factor for hepatitis C in community pharmacies is cost-effective. *J Viral Hepat.* Jan 2020;27(1):36-44.

17. O'Sullivan M, Jones AM, Gage H, et al. ITTREAT (Integrated Community Test - Stage - TREAT) Hepatitis C service for people who use drugs: Real-world outcomes. *Liver Int.* May 2020;40(5):1021-1031.

18. Mohamed Z, Scott N, Al-Kurdi D, et al. Cost-effectiveness of strategies to improve HCV screening, linkage-to-care and treatment in remand prison settings in England. *Liver Int.* Dec 2020;40(12):2950-2960.

19. Toni T, Welch D, Strelkowa N, Ipsen A, Stumpf MP. Approximate Bayesian computation scheme for parameter inference and model selection in dynamical systems. *J R Soc Interface.* Feb 6 2009;6(31):187-202.

20. Marsden J, Stillwell G, Jones H, et al. Does exposure to opioid substitution treatment in prison reduce the risk of death after release? A national prospective observational study in England. *Addiction.* Aug 2017;112(8):1408-1418.

21. Hickman M, Hope V, Brady T, et al. Hepatitis C virus (HCV) prevalence, and injecting risk behaviour in multiple sites in England in 2004. *J. Viral. Hepat.* Sep 2007;14(9):645-652.

22. Mills HL, Colijn C, Vickerman P, Leslie D, Hope V, Hickman M. Respondent driven sampling and community structure in a population of injecting drug users, Bristol, UK. *Drug and alcohol dependence.* Jun 22 2012;126(3):324-332.

23. Harris RJ, Harris HE, Mandal S, et al. Monitoring the hepatitis C epidemic in England and evaluating intervention scale-up using routinely collected data. *J Viral Hepat.* May 2019;26(5):541-551.

24. UK Health Security Agency. Hepatitis C: Operational Delivery Network (ODN) profile tool (<https://www.gov.uk/government/publications/hepatitis-c-commissioning-template-for-estimating-disease-prevalence>). 2018.

25. UKHSA. *Opiate and crack cocaine use: prevalence estimates (*[*https://www.gov.uk/government/publications/opiate-and-crack-cocaine-use-prevalence-estimates)*2023](https://www.gov.uk/government/publications/opiate-and-crack-cocaine-use-prevalence-estimates)2023).

26. Santo T, Jr., Clark B, Hickman M, et al. Association of Opioid Agonist Treatment With All-Cause Mortality and Specific Causes of Death Among People With Opioid Dependence: A Systematic Review and Meta-analysis. *JAMA Psychiatry.* Sep 1 2021;78(9):979-993.

27. Kemp PA, Neale J, Robertson M. Homelessness among problem drug users: prevalence, risk factors and trigger events. *Health Soc Care Community.* Jul 2006;14(4):319-328.

28. Craine N, Hickman M, Parry J, et al. Incidence of hepatitis C in drug injectors: the role of homelessness, opiate substitution treatment, equipment sharing, and community size. *Epidemiol. Infect.* 2009;137(9):1255-1265.

29. Cornish R, Macleod J, Strang J, Vickerman P, Hickman M. Risk of death during and after opiate substitution treatment in primary care: prospective observational study in UK General Practice Research Database. *BMJ.* 2010;341:c5475.

30. Larney S, Toson B, Burns L, Dolan K. Effect of prison‐based opioid substitution treatment and post‐release retention in treatment on risk of re‐incarceration. *Addiction.* 2012;107(2):372-380.

31. Merrall EL, Kariminia A, Binswanger IA, et al. Meta-analysis of drug-related deaths soon after release from prison. *Addiction.* Sep 2010;105(9):1545-1554.

32. Sordo L, Barrio G, Bravo MJ, et al. Mortality risk during and after opioid substitution treatment: systematic review and meta-analysis of cohort studies. *BMJ.* Apr 26 2017;357:j1550.

33. Degenhardt L, Grebely J, Stone J, et al. Global patterns of opioid use and dependence: harms to populations, interventions, and future action. *Lancet.* Oct 26 2019;394(10208):1560-1579.

34. Aisyah DN, Shallcross L, Hully AJ, O'Brien A, Hayward A. Assessing hepatitis C spontaneous clearance and understanding associated factors—a systematic review and meta‐analysis. *Journal of viral hepatitis.* 2018;25(6):680-698.

35. Micallef JM, Kaldor J, Dore GJ. Spontaneous viral clearance following acute hepatitis C infection: a systematic review of longitudinal studies. *J. Viral Hepat.* 2006;13:34-41.

36. Smith DJ, Jordan A, Frank M, Hagan H. Spontaneous viral clearance of hepatitis C virus (HCV) infection among people who inject drugs (PWID) and HIV-positive men who have sex with men (HIV+ MSM): a systematic review and meta-analysis. *BMC Infect Dis.* 2016;16(471):1-13.

37. Platt L, Minozzi S, Reed J, et al. Needle syringe programmes and opioid substitution therapy for preventing hepatitis C transmission in people who inject drugs. *Cochrane Database Syst Rev.* Sep 18 2017;9:CD012021.

38. Arum C, Fraser H, Artenie AA, et al. Homelessness, unstable housing, and risk of HIV and hepatitis C virus acquisition among people who inject drugs: a systematic review and meta-analysis. *The Lancet Public Health.* 2021;6(5):e309-e323.

39. Stone J, Fraser H, Lim AG, et al. Incarceration history and risk of HIV and hepatitis C virus acquisition among people who inject drugs: a systematic review and meta-analysis. *The Lancet. Infectious diseases.* Dec 2018;18(12):1397-1409.

40. Nielsen SF, Hjorthoj CR, Erlangsen A, Nordentoft M. Psychiatric disorders and mortality among people in homeless shelters in Denmark: a nationwide register-based cohort study. *Lancet.* Jun 25 2011;377(9784):2205-2214.

41. Smith DJ, Combellick J, Jordan AE, Hagan H. Hepatitis C virus (HCV) disease progression in people who inject drugs (PWID): A systematic review and meta-analysis. *Int J Drug Policy.* Oct 2015;26(10):911-921.

42. van der Meer AJ, Veldt BJ, Feld JJ, et al. Association between sustained virological response and all-cause mortality among patients with chronic hepatitis C and advanced hepatic fibrosis. *JAMA.* Dec 26 2012;308(24):2584-2593.

43. Morgan RL, Baack B, Smith BD, Yartel A, Pitasi M, Falck-Ytter Y. Eradication of hepatitis C virus infection and the development of hepatocellular carcinoma: a meta-analysis of observational studies. *Ann Intern Med.* Mar 5 2013;158(5 Pt 1):329-337.

44. Wright M, Grieve R, Roberts J, Main J, Thomas HC, Investigators UKMHCT. Health benefits of antiviral therapy for mild chronic hepatitis C: randomised controlled trial and economic evaluation. *Health technology assessment (Winchester, England).* 2006;10:1-113, iii.
